# Stereochemistry of Benzylic Carbon Substitution Coupled with Ring Modification of 2-Nitrobenzyl Groups as Key Determinants for Fast-Cleaving Reversible Terminators**

**DOI:** 10.1002/anie.201106516

**Published:** 2012-01-09

**Authors:** Brian P Stupi, Hong Li, Jinchun Wang, Weidong Wu, Sidney E Morris, Vladislav A Litosh, Jesse Muniz, Megan N Hersh, Michael L Metzker

**Affiliations:** LaserGen, Inc. HoustonTX 77054 (USA); Human Genome Sequencing Center (USA)One Baylor Plaza, N1409, Houston, TX 77030 (USA); Department of Molecular & Human Genetics, Baylor College of MedicineOne Baylor Plaza, N1409, Houston, TX 77030 (USA); + Department of Medicinal Chemistry and Pharmacognosy, University of IllinoisChicago, IL 60612 (USA)

**Keywords:** cleavage reactions, nucleotides, photochemistry, reversible terminators

Next-generation sequencing (NGS) technologies have facilitated important biomedical discoveries, yet high error rates and slow cycle times warrant further improvements in the chemistry.1a Such technologies that employ the cyclic reversible termination (CRT) method1a,b typically utilize 3′-O-blocked reversible terminators.2a–c Recently, we described a novel 3′-OH-unblocked reversible terminator based on 2-nitrobenzyl-modified 5-hydroxymethyl-2′-deoxyuridine (HOMedU) 5′-triphosphate.^3^ Our study revealed that the proximity of the 2-nitrobenzyl group to the nucleobase and the size of the alkyl group attached to its α-methylene carbon are important structural features that confer the unique properties of single-base termination, efficient incorporation, and high nucleotide selectivity (i.e., high fidelity) to these 3′-OH-unblocked nucleotides.^3^ These properties have the potential to improve accuracy and read-lengths in the CRT method. As HOMedU is a naturally found hypermodified nucleoside,4a we set out to identify other such examples. 5-Hydroxymethyl-2′-deoxycytidine (HOMedC) is found naturally in the genomes of T-even bacteriophages4a,b and mammals.^5^ Pyrrolopyrimidine (7-deazapurine) is also found naturally in nucleoside antibiotics^6^ and tRNAs.^7^ Thus, various analogues of 2-nitrobenzyl-modified 7-deaza-7-hydroxymethyl-2′-deoxyadenosine (*C*^7^-HOMedA),^8^ HOMedC, 7-deaza-7-hydroxymethyl-2′-deoxyguanosine (*C*^7^-HOMedG),^9^ and HOMedU were synthesized with the goal of developing a complete set of reversible terminators (Figure 1).

**Figure 1:**
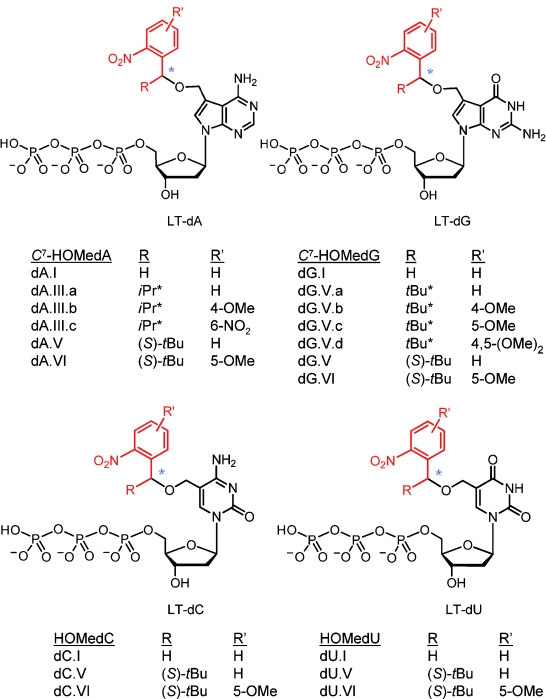
Structures of 2-nitrobenzyl-modified HOMedNTP analogues, called Lightning Terminators (LT). R=H, isopropyl, or *tert*-butyl; R′=H, 4-OMe, 5-OMe, 4,5-di-OMe, or 6-NO_2_; see keys for specific examples. “*” denotes two different diastereomers (*R* and *S*). Red chemical structures denote terminating functional groups that are cleaved upon exposure to UV light. Syntheses of all nucleotides are described in the Supporting Information, except for those of dU.I and dU.V, which have been previously reported.^3^

Ideally, these terminators should exhibit fast nucleotide-incorporation kinetics, single-base termination, high nucleotide selectivity, and rapid terminating group cleavage. For the latter, the degree to which the rate of photochemical cleavage is altered depends on numerous factors including substitution of the benzylic carbon,10a–c attachment of functional group(s) to the benzyl ring,10b–d and nature of the leaving group,10a as well as pH,10a,d,e solvent,10c,f,g and light intensity.10e,g One property, however, that has not been studied is stereochemistry, whereby substitution of 2-nitrobenzyl’s benzylic or α-carbon results in a chiral center. For the case of nucleotide synthesis, coupling of a racemic α-substituted 2-nitrobenzyl alcohol would result in two diastereomers, which differ only by the absolute configuration (*R* or *S*) at the benzylic carbon (“*” in Figure 1). Here, we describe our efforts toward improving the photochemical-cleavage properties by examining various ring-substituted, stereospecific α-isopropyl- and α-*tert*-butyl-2-nitrobenzyl-modified reversible terminators.

Unlike our work with α-substituted HOMedU analogues,^3^ we identified chromatographic conditions to separate *C*^7^-HOMedA analogues into single diastereomeric nucleotides, with the first eluting isomer denoted as ds1 and the second as ds2. To evaluate photochemical-cleavage effects, three 2-nitrobenzyl-modified *C*^7^-HOMedA analogues were synthesized and separated into single diastereomers, dA.III.a (α-isopropyl), dA.III.b (α-isopropyl-4-OMe), and dA.III.c (α-isopropyl-6-NO_2_), along with the parent dA.I (see the Supporting Information). These *C*^7^-HOMedATP analogues were applied in incorporation assays followed by photochemical-cleavage experiments in sodium azide solution (Table 1). In all cases, the ds2 isomers of dA.III.a, dA.III.b, and dA.III.c showed faster photochemical cleavage rates (i.e., lower DT_50_ values) by factors of 2.0, 6.4, and 1.2, respectively, compared to their ds1 counterparts. Interestingly, the ds1 isomers exhibited similar (dA.III.c) or higher (dA.III.a and dA.III.b) DT_50_ values than the parent dA.I. These data provide evidence that the stereochemistry of the α-substituted isopropyl group is an important determinant, and coupled with a 4-OMe substitution, analogue dA.III.b ds2 produced the lowest DT_50_ value for the *C*^7^-HOMedA set.

**Table 1:** Rates of photochemical cleavage for *C*^7^-HOMedA analogues.[a]

*C*^7^-HOMedA Analogue	DT_50_ in 1 mm NaN_3_	
	No DTT	50 mm DTT
dA.I	3.6±0.1	3.5±0.1
dA.III.a ds1	4.5±0.2	4.4±0.2
dA.III.a ds2	2.2±0.1	2.1±0.1
dA.III.b ds1	7.0±0.3	6.1±0.4
dA.III.b ds2	1.1±0.1	1.0±0.1
dA.III.c ds1	3.4±0.2	3.0±0.2
dA.III.c ds2	2.8±0.2	2.5±0.1

[a] A DT_50_ value is defined as the point in time at which 50 % of the 2-nitrobenzyl groups have been photochemically cleaved from the extended primer/template complex. Lower DT_50_ values indicate faster photochemical cleavage rates. DTT=dithiothreitol.

Our previous work demonstrated that the α-*tert*-butyl analogue dU.V exhibited excellent CRT properties, such as single-base termination and high nucleotide selectivity.^3^ This allowed us to further examine the stereospecific effect using a different α-substitution group coupled with various OMe ring substitutions by synthesizing four α-*tert*-butyl *C*^7^-HOMedG analogues, dG.V.a—dG.V.d, along with the parent dG.I (Figure 1). Consistent with α-isopropyl-*C*^7^-HOMedATP analogues, photochemical-cleavage experiments of the ds2 isomers dG.V.a—dG.V.d showed faster rates by factors of 3.1, 4.5, 4.4, and 3.0, respectively, compared with their ds1 counterparts (Table 2). The photochemical-cleavage rates of both 5-OMe ds1 and ds2 isomers were 1.4 times faster than those of their respective 4-OMe isomers, demonstrating that ring position influences the cleavage rate. The bis-substituted 4,5-di-OMe ds1 isomer showed faster rates than the monosubstituted 4-OMe (2.0-fold) and 5-OMe (1.5-fold) ds1 isomers. Conversely, both 5-OMe and 4,5-di-OMe ds2 isomers exhibited the identical DT_50_ value of just 0.8 s. We note that in the absence of an α substituent, Hasan et al. reported a rate increase of only 1.2-fold for a 5-OMe-2-nitrobenzyl analogue over its corresponding parent.10b Comparison of ds1 and ds2 isomers of dG.V.c with dG.V.a revealed higher rate increases of 3.6-fold and 4.4-fold, respectively, suggesting that the stereospecific *tert*-butyl group enhances the effect of the 5-OMe group. With four-color CRT applications, this combination provides good flexibility for the utility of the ring system, as a linker can be attached to the 4-position to create dye-labeled analogues.1a

**Table 2:** Rates of photochemical cleavage for *C*^7^-HOMedG analogues.

*C*^7^-HOMedG Analogue	DT_50_ in 1 mm NaN_3_	
	No DTT	50 mm DTT
dG.I	9.2±0.3	8.1±0.2
dG.V.a ds1	11.0±0.4	10.7±0.2
dG.V.a ds2	3.6±0.3	3.5±0.3
dG.V.b ds1	4.9±0.3	4.6±0.3
dG.V.b ds2	1.1±0.1	1.3±0.2
dG.V.c ds1	3.5±0.3	3.0±0.1
dG.V.c ds2	0.8±0.1	0.8±0.1
dG.V.d ds1	2.4±0.1	2.3±0.2
dG.V.d ds2	0.8±0.1	0.8±0.1

To determine the stereochemistry of these α-*tert*-butyl *C*^*7*^-HOMedG analogues, the (1*S*)-camphanate of (*R*/*S*)-1-(5-methoxy-2-nitrophenyl)-2,2-dimethyl-1-propanol was resolved into its enantiopure *S* alcohol by fractional crystallization^11^ (Figure S1 in the Supporting Information). This *S* alcohol and (*S*)-α-*tert*-butyl-2-nitrobenzyl alcohol^3^ were each coupled to *C*^7^-HOMedG (Figure 1). RP-HPLC analysis of their corresponding triphosphates revealed that both ds2 isomers of dG.V.a and dG.V.c have peak retention times identical to those of dG.V and dG.VI, respectively, thus indicating that both ds2 isomers have the same *S* configuration at the α-carbon. By inference, the corresponding ds1 isomers of dG.V.a and dG.V.c have been assigned the *R* configuration.

These *S* alcohols were then coupled to the remaining nucleosides to examine the effect of the nucleotide leaving group on the rate of photochemical cleavage. For example, photochemical-cleavage experiments revealed that DT_50_ values for the parent 2-nitrobenzyl analogues ranged from 2.0 s for dC.I to 9.2 s for dG.I, suggesting that the leaving group can influence the rate of photochemical cleavage (Figure 2). Substitution of the benzylic carbon with a *tert*-butyl group in the stereospecific *S* configuration, denoted simply as (*S*)-α-*tert*-butyl, resulted in increased cleavage rates by factors of 1.5–3.1, and the additional substitution with a 5-OMe group further increased rates by factors of 3.0–11.5 compared with the parent analogues. The greatest rate improvement was observed in the set of *C*^7^-HOMedG analogues, for which DT_50_ values were reduced from 9.2 to 0.8 s (Figure 2, blue bars). The complete set of (*S*)-α-*tert*-butyl-5-OMe reversible terminators showed a more narrow range of DT_50_ values from 0.6 to 0.8 s. These data suggest that the combined effects of the (*S*)-α-*tert*-butyl and 5-OMe groups play an important role in diminishing the variation in cleavage rates observed with particular nucleotide leaving groups, which has the practical application of providing normalized and faster cleavage conditions for the CRT cycle.

**Figure 2:**
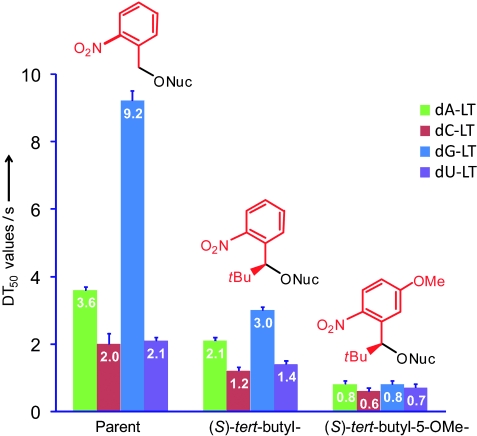
Rates for the photochemical cleavage of the partent 2-nitrobenzyl group as well as for the (*S*)-α-*tert*-butyl-, and (*S*)-α-*tert*-butyl-5-OMe-modified 2-nitrobenzyl groups on *C*^*7*^-HOMedA, HOMedC, *C*^7^-HOMedG, and HOMedU nucleosides. Nuc=nucleotide.

Following brief exposure to UV light, transient products were observed from incorporation assays for (*S*)-α-*tert*-butyl-5-OMe-*C*^7^-HOMedA, -HOMedC, and -HOMedU (Figure 3 A), but not for -*C*^7^-HOMedG. As the only difference was the just-incorporated nucleotide, we hypothesize that the faster cleaving (*S*)-α-*tert*-butyl-5-OMe-2-nitrobenzyl group produces a more reactive 2-nitrosoketone by-product, which attacks the 3′-terminal nucleotide of the growing primer strand. To investigate conditions for quenching the nitroso intermediate, a number of amino and thiol agents were tested (Figure S2 in the Supporting Information). Of these, only dithiothreitol (DTT)^12^ eliminated the transient product (Figure 3 B). To test rate effects, photochemical-cleavage experiments were repeated for all compounds in the presence of DTT, of which DT_50_ values for several parent and ds1 isomers were reduced (Tables 1 and 2, and Table S3). Corrie and colleagues proposed that DTT attacks the nitroso group by nucleophilic addition,^13^ thereby, providing in our case protection against such undesired reactions.

**Figure 3:**
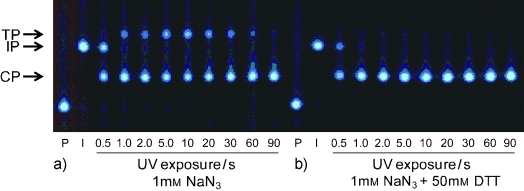
Elimination of transient product (TP) with DTT. Fluorescent gel images of the photochemical cleavage of dU.VI incorporated by Therminator polymerase in the presence of a) 1 mm NaN_3_ and b) 1 mm NaN_3_, 50 mm DTT recorded at different times. Lanes: “P” (primer) contains Therminator bound to oligoTemplate-4 hybridized with BODIPY-FL-labeled primer-1 in 1x ThermoPol buffer,^3^ “I” (incorporation) contains that found in lane “P” plus 100 nm dU.VI, and time-point lanes contain that found in lane “I” plus exposure times to 0.70 W cm^−2^ 365 nm light. “IP” denotes incorporated product and “CP” denotes cleaved product.

We have demonstrated that the stereospecific *S* configuration of an α-*tert*-butyl group and the ring modification of a 5-OMe group are major determinants for creating a complete set of fast-cleaving reversible terminators with normalized rates. We believe this stereospecific effect, however, is not limited to just an α-*tert*-butyl group. We have shown several examples of α-isopropyl ds2 isomers, presumed to have *S* configuration, which also have faster rates for photochemical cleavage than their ds1 isomers. In the presence of DTT, the reactive nitrosoketone by-product can be eliminated effectively during photochemical cleavage, creating appropriate conditions to maintain the biological integrity of the CRT reaction. We note that these 3′-OH-unblocked (*S*)-α-*tert*-butyl-5-OMe-2-nitrobenzyl-modified nucleotides also exhibit single-base termination, fast incorporation kinetics, and high nucleotide selectivity (unpublished results). Thus, this work not only expands the repertoire of 2-nitrobenzyl modifications that yield faster-cleaving protecting groups, but when coupled with reversible terminators, yield faster cycle times for NGS instrument systems.

## Supplementary material

Detailed facts of importance to specialist readers are published as ”Supporting Information”. Such documents are peer-reviewed, but not copy-edited or typeset. They are made available as submitted by the authors.
