# Supplementary material for: Stereochemistry of Benzylic Carbon Substitution Coupled with Ring Modification of 2-Nitrobenzyl Groups as Key Determinants for Fast-Cleaving Reversible Terminators
Source: Angew Chem Int Ed Engl. 2012 Jan 9;51(7):1724–7. doi: 10.1002/anie.201106516 (PMC3326374; doi:10.1002/anie.201106516)
Supplement: Supplementary file 1 [file anie0051-1724-SD1.pdf]

Angewandte Chemie
Eine Zeitschrift der Gesellschaft Deutscher Chemiker

Supporting Information

© Wiley-VCH 2012

69451 Weinheim, Germany

Stereochemistry of Benzylic Carbon Substitution Coupled with Ring Modification of 2-Nitrobenzyl Groups as Key Determinants for Fast-Cleaving Reversible Terminators**

*Brian P. Stupi, Hong Li, Jinchun Wang, Weidong Wu, Sidney E. Morris, Vladislav A. Litosh, Jesse Muniz, Megan N. Hersh, and Michael L. Metzker**

anie_201106516_sm_miscellaneous_information.pdf

Table of Contents

I. Complete authors' list of selected references 3

II. Reagents and materials 3

III. Spectroscopic and analytical instrumentation 3

IV. Synthesis of α-substituted 2-nitrobenzyl alcohols 4

V. Synthesis of 7-HOMe-7-deaza-2'-deoxyadenosine triphosphate analogs 12

VI. Synthesis of 7-HOMe-7-deaza-2'-deoxyguanosine triphosphate analogs 26

VII. Synthesis of 5-HOMe-2'-deoxyuridine triphosphate analog 46

VIII. Synthesis of 5-HOMe-2'-deoxycytidine triphosphate analogs 48

IX. UV photocleavage studies 57

X. References 62

I. Complete authors' list of selected references

Complete references containing more than ten authors are provided below.

Reference 2a. J. Ju, D. H. Kim, L. Bi, Q. Meng, X. Bai, Z. Li, X. Li, M. S. Marma, S. Shi, J. Wu, J. R. Edwards, A. Romu, N. J. Turro, *Proc. Natl. Acad. Sci. USA* **2006**, *103*, 19635-19640.

Reference 2b. J. Guo, N. Xu, Z. Li, S. Zhang, J. Wu, D. H. Kim, M. Sano Marma, Q. Meng, H. Cao, X. Li, S. Shi, L. Yu, S. Kalachikov, J. J. Russo, N. J. Turro, J. Ju, *Proc. Natl Acad. Sci. USA* **2008**, *105*, 9145-9150.

Reference 2c. D. R. Bentley, S. Balasubramanian, H. P. Swerdlow, G. P. Smith, J. Milton, C. G. Brown, K. P. Hall, D. J. Evers, C. L. Barnes, H. R. Bignell, J. M. Boutell, J. Bryant, R. J. Carter, R. K. Cheetham, A. J. Cox, D. J. Ellis, M. R. Flatbush, N. A. Gormley, S. J. Humphray, L. J. Irving, M. S. Karbelashvili, S. M. Kirk, H. Li, X. Liu, K. S. Maisinger, L. J. Murray, B. Obradovic, T. Ost, M. L. Parkinson, M. R. Pratt, I. M. J. Rasolonjatovo, M. T. Reed, R. Rigatti, C. Rodighiero, M. T. Ross, A. Sabot, S. V. Sankar, A. Scally, G. P. Schroth, M. E. Smith, V. P. Smith, A. Spiridou, P. E. Torrance, S. S. Tzonev, E. H. Vermaas, K. Walter, X. Wu, L. Zhang, M. D. Alam, C. Anastasi, I. C. Aniebo, D. M. D. Bailey, I. R. Bancarz, S. Banerjee, S. G. Barbour, P. A. Baybayan, V. A. Benoit, K. F. Benson, C. Bevis, P. J. Black, A. Boodhun, J. S. Brennan, J. A. Bridgham, R. C. Brown, A. A. Brown, D. H. Buermann, A. A. Bundu, J. C. Burrows, N. P. Carter, N. Castillo, M. C. E. Catenazzi, S. Chang, R. N. Cooley, N. R. Crake, O. O. Dada, K. D. Diakoumakos, B. Dominguez-Fernandez, D. J. Earnshaw, U. C. Egbujor, D. W. Elmore, S. S. Etchin, M. R. Ewan, M. Fedurco, L. J. Fraser, K. V. F. Fajardo, W. S. Furey, D. George, K. J. Gietzen, C. P. Goddard, G. S. Golda, P. A. Granieri, D. E. Green, D. L. Gustafson, N. F. Hansen, K. Harnish, C. D. Haudenschild, N. I. Heyer, M. M. Hims, J. T. Ho, A. M. Horgan, et al., *Nature* **2008**, *456*, 53-59.

II. Reagents and materials

All reagents were purchased from commercial sources and used as received, unless otherwise noted.

III. Spectroscopic and analytical instrumentation

^{1}H NMR, ^{13}C NMR, and ^{31}P NMR spectra were recorded on a Bruker DPX 400 spectrometer as previously described.[1] Mass spectra analyses were provided by the Mass Spectrometry Laboratory at the MD Anderson Cancer Center (Houston, TX) and the Core Mass Spectrometry Facility at Rice University (Houston, TX). X-ray crystallography was performed by the X-ray Diffraction Laboratory at Texas A&M University (College Station, TX). UV/Vis measurements were taken using a Beckman DU-800 spectrophotometer. Anion exchange chromatography was performed using a Q Sepharose FF column (2.5 × 20 cm) with a linear gradient of 75% triethylammonium bicarbonate (TEAB, 0.1 M) in 25% acetonitrile to 75% TEAB (1.5 M) in 25%

acetonitrile over 240 min at a flow rate of 4.5 mL per min. Reverse-phase high performance liquid chromatography (RP-HPLC) was performed using a Beckman System Gold equipped with a 128 solvent module and 166 UV detector or 168 photodiode array UV/Vis detector. RP-HPLC for nucleosides and nucleotide analogs was performed using a 4.6 mm × 250 mm Aquapore OD-300 C_{18} column, with buffer A containing 100 mM triethylammonium acetate (TEAA), pH 7.0, and buffer B containing 100 mM TEAA, pH 7.0, 70% acetonitrile (v/v).

IV. Synthesis of α-substituted 2-nitrobenzyl alcohols

(R/S)-1-(2-Nitrophenyl)-2-methyl-1-propanol

Synthesis of (*R*/*S*)-1-(2-nitrophenyl)-2-methyl-1-propanol was previously reported.[2]

(R/S)-1-(2-Nitrophenyl)-2,2-dimethyl-1-propanol

Synthesis of (*R*/*S*)-1-(2-nitrophenyl)-2,2-dimethyl-1-propanol was previously reported.[2]

(R/S)-1-(2,6-Dinitrophenyl)-2-methyl-1-propanol

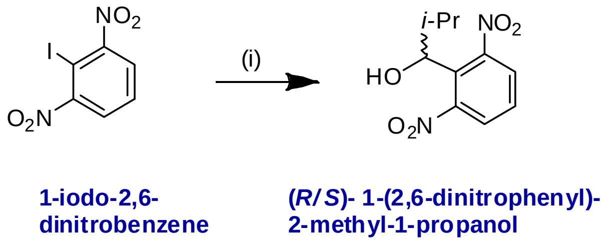

Scheme S1. Synthesis of (*R*/*S*)-1-(2,6-dinitrophenyl)-2-methyl-1-propanol. *Reagents and conditions*: (*i*) PhMgBr, THF, minus 50°C; *i*-PrCHO, minus 50°C to room temperature, 30%.

To a solution of 1-iodo-2,6-dinitrobenzene[3] (1.55 g, 5.27 mmol) in anhydrous THF (18 mL) at minus 50°C under a nitrogen atmosphere, phenylmagnesium bromide (2 M in THF, 3.2 mL, 6.4 mmol) was added dropwise at a rate such that the temperature would not exceed minus 45°C. Upon completion of the addition, the mixture was stirred at minus 50°C for five min, followed by addition of isobutyraldehyde (0.96 mL, 11 mmol). The mixture was gradually warmed up to room temperature, quenched with saturated NH_4Cl solution (10 mL), and then diluted with water (50 mL). The mixture was extracted with CH_2Cl_2 (100 mL) three times. The combined organic phase was washed with brine (50 mL), dried over Na_2SO_4, concentrated *in*

vacuo, and the residue was purified by silica gel column chromatography to yield (*R*/*S*)-1-(2,6-dinitrophenyl)-2-methyl-1-propanol (0.375 g, 30%) as a yellow oil.

^{1}H NMR (400 MHz, $CDCl_3$): δ 7.82 (d, 2 H, *J* = 8.0 Hz, Ph-H), 7.59 (t, 1 H, *J* = 8.0 Hz, Ph-H), 4.83 (dd, 1 H, *J* = 9.2 and 7.6 Hz, Ph-CH), 2.87 (d, 1 H, *J* = 7.6 Hz, OH), 2.19 (m, 1 H, CH), 1.12 (d, 3 H, *J* = 6.4 Hz, CH_3), 0.76 (d, 3 H, *J* = 6.8 Hz, CH_3).

(R/S)-1-(4-Methoxy-2-nitrophenyl)-2-methyl-1-propanol

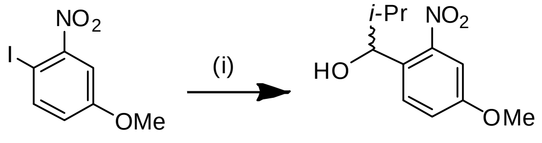

4-iodo-3-nitroanisole

(*R*/*S*)- 1-(4-methoxy-2-nitrophenyl)-2-methyl-1-propanol

Scheme S2. Synthesis of (*R*/*S*)-1-(4-methoxy-2-nitrophenyl)-2-methyl-1-propanol. *Reagents and conditions*: (*i*) PhMgCl, THF, minus 40°C; *i*-PrCHO, minus 40°C to room temperature, 67%.

To a solution of 4-iodo-3-nitroanisole (2.79 g, 10.0 mmol) in anhydrous THF (20 mL) at minus 40°C under a nitrogen atmosphere, phenylmagnesium chloride (2 M in THF, 6.0 mL, 12 mmol) was added dropwise at a rate such that the temperature would not exceed minus 35°C. Upon completion of the addition, the mixture was stirred at minus 40°C for five min, followed by addition of isobutyraldehyde (1.8 mL, 20 mmol). The mixture was gradually warmed to room temperature, quenched with saturated NH_4Cl solution (5.0 mL), diluted with CH_2Cl_2 (100 mL) and washed with water (100 mL). The organic phase was separated, and the aqueous phase was extracted with CH_2Cl_2 (50 mL) three times. The combined organic phase was washed with brine (40 mL), dried over Na_2SO_4, concentrated *in vacuo*, and the residue was purified by silica gel column chromatography to yield (*R*/*S*)-1-(4-methoxy-2-nitrophenyl)-2-methyl-1-propanol (1.5 g, 67%) as a light yellow oil.

^{1}H NMR (400 MHz, $CDCl_3$): δ 7.61 (d, 1 H, *J* = 8.8 Hz, Ph-H), 7.34 (d, 1 H, *J* = 2.8 Hz, Ph-H), 7.15 (dd, 1 H, *J* = 8.8 and 2.8 Hz, Ph-H), 4.92 (dd, 1 H, *J* = 5.6 and 3.2 Hz, Ph-CH), 2.46 (br s, 1 H, OH), 2.00 (m, 1 H, CH), 0.97 (d, 3 H, *J* = 6.4 Hz, CH_3), 0.86 (d, 3 H, *J* = 6.8 Hz, CH_3).

(R/S)-1-(4-Methoxy-2-nitrophenyl)-2,2-dimethyl-1-propanol

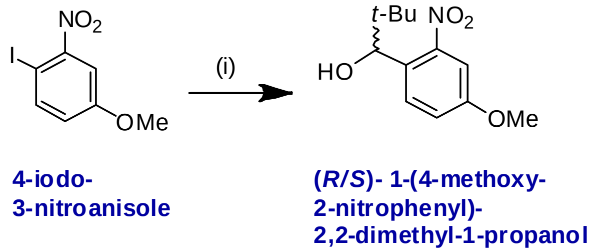

Scheme S3. Synthesis of (*R*/*S*)-1-(4-methoxy-2-nitrophenyl)-2,2-dimethyl-1-propanol. *Reagents and conditions*: (*i*) PhMgCl, THF, minus 40°C; $(CH_3)_3$CCHO, minus 40°C to room temperature, 74%.

To a solution of 4-iodo-3-nitroanisole (2.38 g, 8.50 mmol) in anhydrous THF (10 mL) at minus 40°C under a nitrogen atmosphere, phenylmagnesium chloride (2 M in THF, 4.7 mL, 9.4 mmol) was added dropwise at a rate such that the temperature would not exceed minus 35°C. Upon completion of the addition, the mixture was stirred at minus 40°C for one hour, followed by addition of trimethylacetaldehyde (1.13 mL, 10.2 mmol). The mixture was stirred at minus 40°C for two hours and then at room temperature for another one hour. The reaction was quenched with brine (100 mL), and the mixture was extracted with CH_2Cl_2 (40 mL) three times. The combined organic phase was dried over Na_2SO_4 and concentrated *in vacuo*, and the residue was purified by silica gel column chromatography to yield racemic (*R*/*S*)-1-(4-methoxy-2-nitrophenyl)-2,2-dimethyl-1-propanol (1.52 g, 74%).

^{1}H NMR (400 MHz, $CDCl_3$): δ 7.67 (d, 1 H, *J* = 9.2 Hz, Ph-H), 7.22 (d, 1 H, *J* = 2.4 Hz, Ph-H), 7.12 (dd, 1 H, *J* = 8.8 and 2.8 Hz, Ph-H), 5.27 (d, 1 H, *J* = 4.0 Hz, Ph-CH), 3.86 (s, 3 H, OCH_3), 2.01 (d, 1 H, *J* = 4.0 Hz, OH), 0.86 (s, 9 H, $C(CH_3)_3$).

(R/S)-1-(5-Methoxy-2-nitrophenyl)-2,2-dimethyl-1-propanol and (S)-1-(5-methoxy-2-nitrophenyl)-2,2-dimethyl-1-propanol

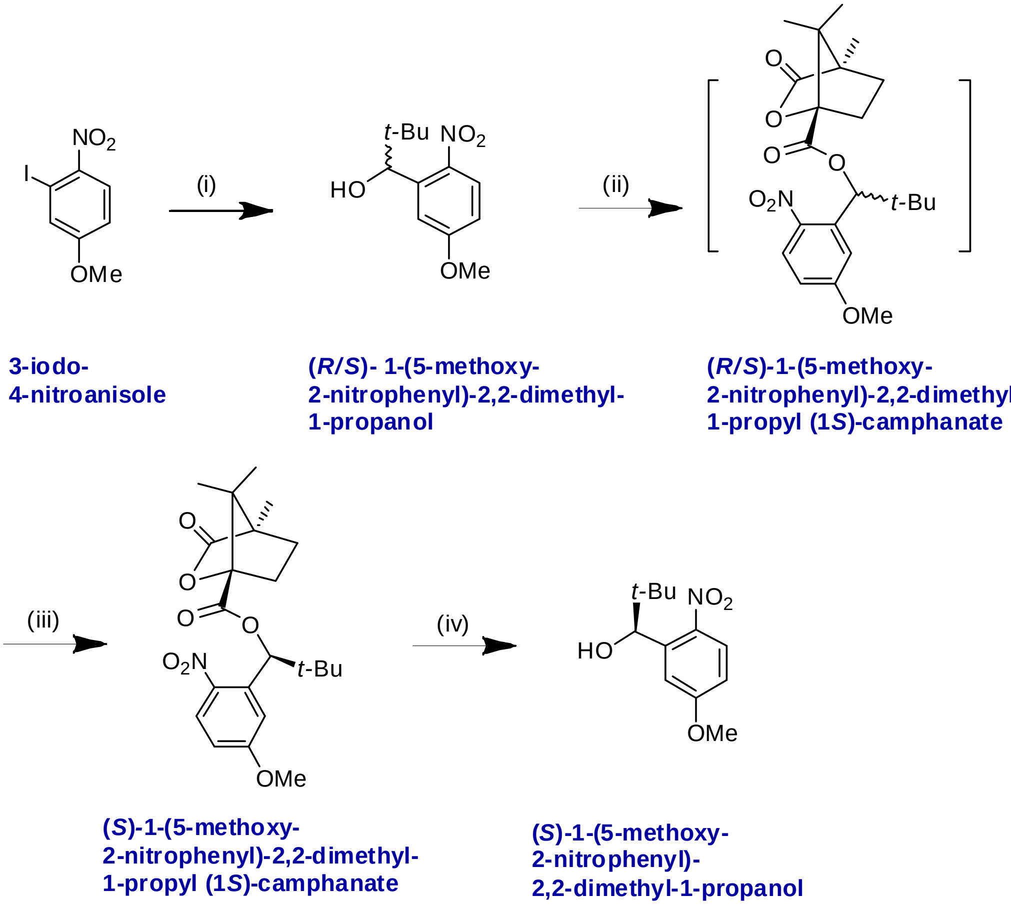

Scheme S4. Synthesis of (*R*/*S*)-1-(5-methoxy-2-nitrophenyl)-2,2-dimethyl-1-propanol and (*S*)-1-(5-methoxy-2-nitrophenyl)-2,2-dimethyl-1-propanol. *Reagents and conditions*: (*i*) PhMgCl, anhydrous THF, minus 40°C; $(CH_3)_3CCHO$, minus 40°C to room temperature, 88%; (*ii*) (1*S*)-camphanic acid chloride, DMAP, CH_2Cl_2, room temperature; (*iii*) fractional crystallization from ethyl acetate/hexane, 43%; (*iv*) K_2CO_3, MeOH, reflux, 99%.

To a solution of 3-iodo-4-nitroanisole (2.79 g, 10.0 mmol) in anhydrous THF (10 mL) at minus 40°C under a nitrogen atmosphere, phenylmagnesium chloride (2 M in THF, 4.2 mL, 8.3 mmol) was added dropwise at a rate such that the temperature would not exceed minus 35°C. Upon completion of the addition, the mixture was stirred at minus 40°C for two hours, followed by addition of trimethylacetaldehyde (1.1 mL, 10 mmol). The mixture was stirred at minus 40°C for two hours and then at room temperature for another one hour. The reaction was then quenched with brine (100 mL), and the mixture was extracted with CH_2Cl_2 (40 mL) three times. The combined organic phase was dried over Na_2SO_4, concentrated *in vacuo*, and the residue

was purified by silica gel column chromatography to yield racemic (*R*/*S*)-1-(5-methoxy-2-nitrophenyl)-2,2-dimethyl-1-propanol (1.76 g, 88%).

^{1}H NMR (400 MHz, $CDCl_3$): δ 7.89 (d, 1 H, *J* = 9.2 Hz, Ph-H), 7.27 (d, 1 H, *J* = 2.8 Hz, Ph-H), 6.84 (dd, 1 H, *J* = 8.8 and 2.8 Hz, Ph-H), 5.62 (d, 1 H, *J* = 4.0 Hz, PhCH), 3.89 (s, 3 H, OCH_3), 2.08 (d, 1 H, *J* = 4.0 Hz, OH), 0.89 (s, 9 H, $C(CH_3)_3$).

To a solution of racemic (*R*/*S*)-1-(5-methoxy-2-nitrophenyl)-2,2-dimethyl-1-propanol (1.75 g, 7.3 mmol) and DMAP (2.92 g, 23.9 mmol) in anhydrous CH_2Cl_2 (10 mL), (1*S*)-camphanic chloride[4] (2.6 g, 12 mmol) was added, and the mixture was stirred overnight at room temperature under a nitrogen atmosphere. The reaction mixture was diluted with CH_2Cl_2 (50 mL) and washed with saturated $NaHCO_3$ solution (50 mL). The organic phase was dried over Na_2SO_4, concentrated *in vacuo*, and the residue was purified by silica gel column chromatography to yield (*R*/*S*)-1-(5-methoxy-2-nitrophenyl)-2,2-dimethyl-1-propyl (1*S*)-camphanate (2.5 g, 85%, 1:1 mixture of diastereomers). The camphanate was dissolved in ethyl acetate (30 mL) followed by slow addition of hexane (120 mL) with stirring. Needle crystals formed gradually from the solution over a two-hour period. The crystals were collected by filtration to yield pure single diastereomer (*S*)-1-(5-methoxy-2-nitrophenyl)-2,2-dimethyl-1-propyl (1*S*)-camphanate. The filtrate was concentrated *in vacuo*, and the crystallization process was repeated twice to provide additional (*S*)-1-(5-methoxy-2-nitrophenyl)-2,2-dimethyl-1-propyl (1*S*)-camphanate (total 1.08 g, 43%).

^{1}H NMR (400 MHz, $CDCl_3$): δ 8.04 (d, 1 H, *J* = 9.2 Hz, Ph-H), 7.27 (d, 1 H, *J* = 2.8 Hz, Ph-H), 6.88 (dd, 1 H, *J* = 2.8 and 8.8 Hz, Ph-H), 6.81 (3, 1 H, Ph-CH), 3.87 (s, 3 H, OCH_3), 2.36 (m, 1 H, CH), 1.92 (m, 2 H, CH_2), 1.66 (m, 1 H, CH), 1.12 (s, 3 H, CH_3), 1.06 (s, 3 H, CH_3), 1.02 (s, 3 H, CH_3), 0.95 (s, 9 H, $C(CH_3)_3$).

Method for obtaining X-ray crystallography data: Crystallographic measurements were made on a crystal of (*S*)-1-(5-methoxy-2-nitrophenyl)-2,2-dimethyl-1-propyl (1S)-camphanate with dimensions of 0.50 mm x 0.05 mm x 0.05 mm as previously described[2], see Figure S1.

Data collection: CuKα radiation, λ = 1.54178 Å, T = 110 ± 2°K, $2\vartheta_{max}$ = 120.0°, 32,513 reflections collected, 2,913 unique (R_{int} = 0.0517). Final *GooF* = 1.091, *R1* = 0.0681, *wR2* = 0.1695, *R* indices based on 2,913 reflections with I>2sigma(I) (refinement on F^2), 290 parameters, 43 restraints. Lp and absorption corrections applied, μ = 0.819 mm^{-1}. Absolute structure parameter: 0.05 ± 0.09.

X-Ray crystallography data: $C_{22}H_{29}NO_7$, M = 419.46. Orthorhombic, a = 6.29, b = 15.00, c = 22.27 Å (α, β, γ = 90°), V = 2,099.29 Å^3, space group $P2_12_12_1$, Z = 4, D_c = 1.327 g/cm^{-3}, F(000) = 896.

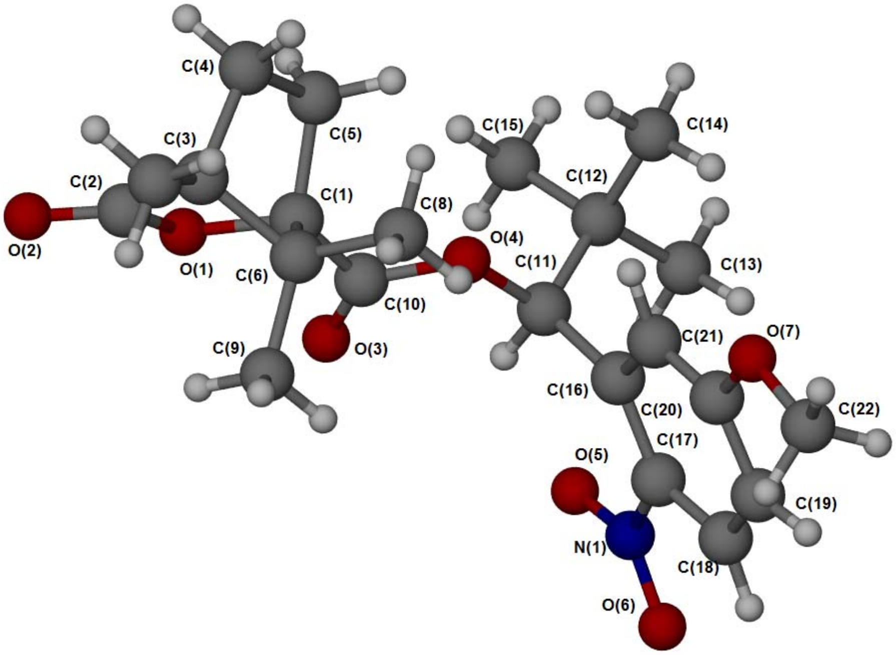

Figure S1. X-ray crystal structure of (*S*)-1-(5-methoxy-2-nitrophenyl)-2,2-dimethyl-1-propyl (1*S*)-camphanate.

A mixture of (*S*)-1-(5-methoxy-2-nitrophenyl)-2,2-dimethyl-1-propyl (1*S*)-camphanate (590 mg, 1.4 mmol) and K_2CO_3 (389 mg, 2.8 mmol) in methanol (MeOH, 25 mL) was heated to reflux for one hour, then cooled down, concentrated *in vacuo*, and diluted with CH_2Cl_2 (50 mL). The organic phase was washed with brine (50 mL), dried over Na_2SO_4, concentrated *in vacuo*, and the residue was purified by silica gel column chromatography to yield enantiopure (*S*)-1-(5-methoxy-2-nitrophenyl)-2,2-dimethyl-1-propanol (333 mg, 99%). ^{1}H NMR was identical with that of the racemic alcohol.

(R/S)-1-(4,5-Dimethoxy-2-nitrophenyl)-2,2-dimethyl-1-propanol

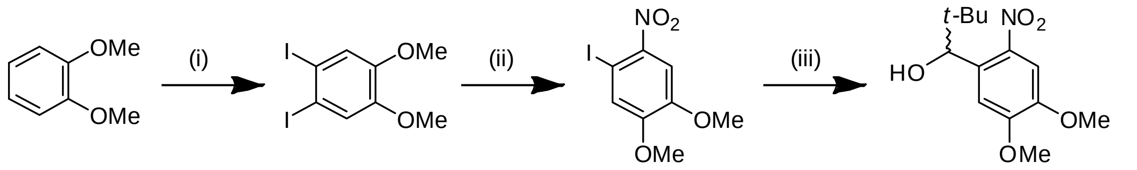

1,2-dimethoxy-benzene | **4,5-diiodo-1,2-dimethoxy-benzene** | **4-iodo-5-nitro-1,2-dimethoxy-benzene** | **(*R*/*S*)- 1-(4,5-dimethoxy-2-nitrophenyl)-2,2-dimethyl-1-propanol**

Scheme S5. Synthesis of (*R*/*S*)-1-(4,5-dimethoxy-2-nitrophenyl)-2,2-dimethyl-1-propanol. *Reagents and conditions:* (*i*) ICl, CH_3COOH, 100°C, 62%; (*ii*) HNO_3, CH_3COOH, room temperature, 75%; (*iii*) PhMgCl, THF, minus 40°C; $(CH_3)_3CCHO$, minus 40°C to room temperature, 20%.

1,2-Dimethoxybenzene (5.0 g, 36 mmol) was dissolved in acetic acid (10 mL), and the solution was cooled in an ice-water bath followed by dropwise addition of iodine chloride (8.7 g, 54 mmol). After 10 min, the reaction mixture was heated to 100°C for two hours and then cooled to room temperature. Needle crystals that precipitated from solution were filtered and washed with acetic acid (5.0 mL) three times. The crystals were dried overnight under high vacuum to yield 4,5-diiodo-1,2-dimethoxybenzene (8.8 g, 62%).

^{1}H NMR (400 MHz, $CDCl_3$): δ 7.28 (s, 2 H, Ph-H), 3.85 (s, 6 H, OCH_3).

4,5-Diiodo-1,2-dimethoxybenzene (8.8 g, 23 mmol) was added into acetic acid (300 mL), and the mixture was heated to 100°C to dissolve the solid. The clear mixture was then cooled to room temperature followed by dropwise addition of nitric acid (68-70%, 120 mL). The reaction mixture was stirred at room temperature overnight and then poured into ice-water (200 mL). The mixture was extracted by CH_2Cl_2 (100 mL) three times. The combined organic phase was washed with saturated $NaHCO_3$ solution (200 mL), brine (100 mL), and dried over Na_2SO_4, concentrated *in vacuo*, and the residue was purified by silica gel column chromatography to yield 4-iodo-5-nitro-1,2-dimethoxybenzene (5.25 g, 75%).

^{1}H NMR (400 MHz, $CDCl_3$): δ 7.60 (s, 1 H, Ph-H), 7.38 (s, 1 H, Ph-H), 3.98 (s, 3 H, OCH_3), 3.92 (s, 3 H, OCH_3).

To a solution of 4-iodo-5-nitro-1,2-dimethoxybenzene (4.6 g, 15 mmol) in anhydrous THF (10 mL) at minus 40°C under a nitrogen atmosphere, phenylmagnesium chloride (2 M in THF, 7.5 mL, 15 mmol) was added dropwise at a rate such that the temperature would not exceed

minus 35°C. Upon completion of the addition, the mixture was stirred at minus 40°C for two hours, followed by addition of trimethyl acetaldehyde (2.0 mL, 18 mmol). The mixture was stirred at minus 40°C for two hours and then at room temperature for another one hour. The reaction was quenched with brine (100 mL), and the mixture was extracted with CH_2Cl_2 (40 mL) three times. The combined organic phase was dried over Na_2SO_4 and concentrated *in vacuo*, and the residue was purified by silica gel column chromatography to yield racemic (*R*/*S*)-1-(4,5-dimethoxy-2-nitrophenyl)-2,2-dimethyl-1-propanol (0.8 g, 20%).

^{1}H NMR (400 MHz, $CDCl_3$): δ 7.41 (1, 1 H, Ph-H), 7.21 (s, 1 H, Ph-H), 5.60 (s, 1 H, PhCH), 3.95 (s, 3 H, OCH_3), 3.92 (s, 3 H, OCH_3), 0.90 (s, 9 H, $(CH_3)_3$).

V. Synthesis of 7-HOMe-7-deaza-2’-deoxyadenosine triphosphate analogs

7-(2-nitrobenzyloxy)methyl-7-deaza-2’-deoxyadenosine-5’-triphosphate

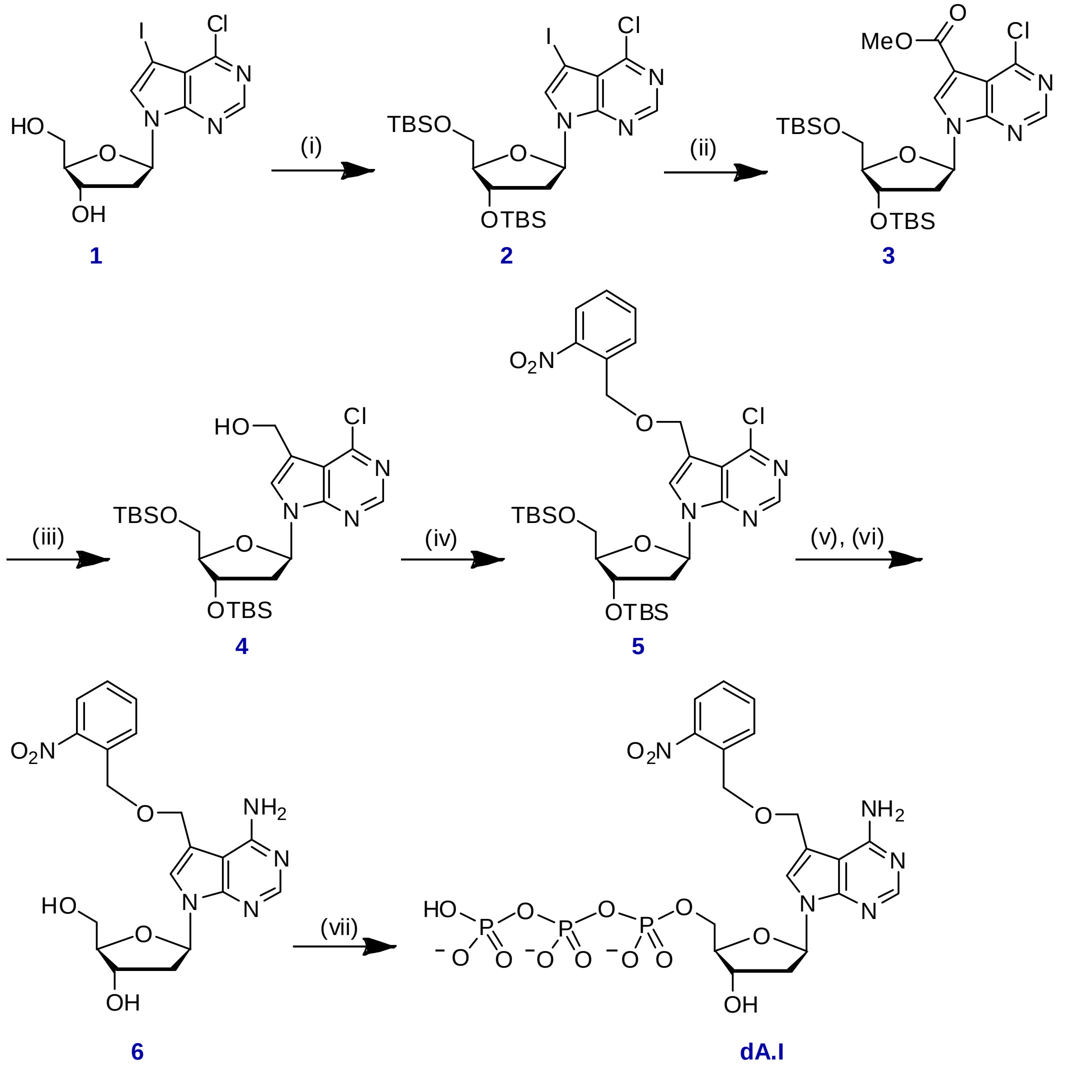

Scheme S6. Synthesis of 7-(2-nitrobenzyloxy)methyl-7-deaza-2’-deoxyadenosine-5’-triphosphate. *Reagents and conditions*: (*i*) TBSCl, imidiazole, DMF, room temperature, 87%; (*ii*) CO, $PdCl_2[PhCN]_2$, MeOH/1,4-dioxane, 50°C, 98%; (*iii*) $LiBH_4$, MeOH, THF, reflux, 45%; (*iv*) 2-nitrobenzyl bromide, *n*-Bu_4NBr, CH_2Cl_2/aq. NaOH, room temperature, 50%; (*v*) *n*-Bu_4NF, THF, 0°C to room temperature; (*vi*) NH_3, 1,4-dioxane/MeOH, 100°C, 91% for two steps; (*vii*) $POCl_3$, $(MeO)_3PO$, minus 40°C; $(n\text{-}Bu_3NH)_2H_2P_2O_7$, *n*-$Bu_3N$, DMF; 1 M $HNEt_3HCO_3$.

Compound **1**[5] (0.79 g, 2.0 mmol) was evaporated from anhydrous pyridine (2.0 mL) three times and dissolved in anhydrous DMF (4.0 mL). *tert*-Butyldimethylsilyl chloride (0.90 g, 6.0 mmol) and imidazole (0.82 g, 12 mmol) were added, and the mixture was stirred at room temperature for 16 hours. The reaction was concentrated *in vacuo* and purified by silica gel

chromatography to yield 9-[β-D-3',5'-*O*-bis-(*tert*-butyldimethylsilyl)-2'-deoxyribofuranosyl]-6-chloro-7-iodo-7-deazapurine **2** (1.08 g, 87%) as a white foam.

^1H NMR (400 MHz, $CDCl_3$): δ 8.61 (s, 1 H, H-2), 7.81 (s, 1 H, H-8), 6.74 (t, 1 H, *J* = 6.4 Hz, H-1'), 4.56 (m, 1 H, H-4'), 4.01 (m, 1 H, H-3'), 3.87 (dd, 1 H, H-5'a), 3.79 (dd, 1 H, H-5'b), 2.39 (m, 2 H, H-2'a and H-2'b), 0.96 (s, 9 H, $(CH_3)_3CSi$), 0.91 (s, 9 H, $(CH_3)_3CSi$), 0.18 (2s, 6 H, $(CH_3)_2Si$), 0.15 (s, 6 H, $(CH_3)_2Si$).

To a solution of compound **2** (1.55 g, 2.48 mmol) in anhydrous 1,4-dioxane (42 mL) and anhydrous MeOH (42 mL), triethylamine (0.87 mL) was added. After stirring for 10 min under a CO atmosphere, bis(benzonitrile)dichloropalladium(II) (0.05 g, 0.13 mmol) was added, and the reaction was stirred at 50°C for 48 hours under a CO atmosphere. The mixture was then concentrated *in vacuo*, and the residue was purified by silica gel chromatography to yield 9-[β-D-3',5'-*O*-bis-(*tert*-butyldimethylsilyl)-2'-deoxyribofuranosyl]-6-chloro-7-methoxycarbonyl-7-deazapurine **3** (1.36 g, 98%) as a viscous oil.

^1H NMR (400 MHz, $CDCl_3$): δ 8.69 (s, 1 H, H-2), 8.31 (s, 1 H, H-8), 6.77 (t, 1 H, *J* = 6.8 Hz, H-1'), 4.58 (m, 1 H, H-4'), 4.06 (m, 1 H, H-3'), 3.90 (s, 3 H, CH_3O), 3.87 (dd, 1 H, H-5'a), 3.81 (dd, 1 H, H-5'b), 2.42 (m, 2 H, H-2'a and H-2'b), 0.93 (s, 18 H, $(CH_3)_3CSi$), 0.13 (s, 6 H, $(CH_3)_2Si$), 0.12 (s, 6 H, $(CH_3)_2Si$).

To a solution of compound **3** (0.28 g, 0.50 mmol) in anhydrous THF (4.0 mL), lithium borohydride (44 mg, 2.0 mmol) was added, followed by MeOH (0.1 mL). The reaction mixture was stirred at room temperature for 10 min and then heated to reflux for 45 min. Upon cooling to room temperature, the mixture was diluted with CH_2Cl_2 (20 mL) and water (2.0 mL). The organic layer was separated, washed with brine (5.0 mL) two times, dried over Na_2SO_4, and concentrated *in vacuo*. The residue was purified by silica gel chromatography to yield 9-[β-D-3',5'-*O*-bis-(*tert*-butyldimethylsilyl)-2'-deoxyribofuranosyl]-6-chloro-7-hydroxymethyl-7-deazapurine **4** (0.12 g, 45%) as a white foam.

^1H NMR (400 MHz, $CDCl_3$): δ 8.62 (s, 1 H, H-8), 7.61 (s, 1 H, H-2), 6.75 (dd, 1 H, *J* = 6.0 and 7.2 Hz, H-1'), 4.96 (AB d, 1 H, *J* = 11.6 Hz, 7-CH_2a), 4.91 (AB d, 1 H, *J* = 11.6 Hz, 7-CH_2b), 4.57 (m, 1 H, H-4'), 4.00 (m, 1 H, H-3'), 3.80 (m, 2 H, H-5'a and H-5'b), 2.44 (m, 1 H, H-2'a), 2.04 (m, 1 H, H-2'b), 0.91 (2 s, 18 H, $(CH_3)_3CSi$), 0.11 (2 s, 12 H, $(CH_3)_2Si$).

To a solution of compound **4** (30 mg, 0.057 mmol) in CH_2Cl_2 (2.0 mL), *n*-Bu_4NBr (9 mg, 0.029 mmol), 2-nitrobenzyl bromide (37 mg, 0.17 mmol) and NaOH solution (1 M, 2.0 mL) were

added. The reaction mixture was stirred vigorously at room temperature for 48 hours in the dark. The organic layer was separated, dried over Na_2SO_4, concentrated *in vacuo,* and the residue was purified by silica gel chromatography to yield 9-[β-D-3',5'-*O*-bis-(*tert*-butyldimethylsilyl)-2'-deoxyribofuranosyl]-6-chloro-7-(2-nitrobenzyloxy)methyl-7-deazapurine **5** (19 mg, 50%) as a viscous oil.

^{1}H NMR (400 MHz, $CDCl_3$): δ 8.63 (s, 1 H, H-2), 8.06 (dd, 1 H, *J* = 8.4 and 1.2 Hz, Ph-H), 7.84 (d, 1 H, *J* = 7.6 Hz, Ph-H), 7.64 (s, 1 H, H-8), 7.62 (m, 1 H, Ph-H), 7.43 (t, 1 H, Ph-H), 6.75 (dd, 1 H, *J* = 7.2 and 6.0 Hz, H-1'), 5.03 (s, 2 H, $PhCH_2$), 4.95 (AB d, 1 H, *J* = 12.0 Hz, 7-CH_2a), 4.88 (AB d, 1 H, *J* = 12.0 Hz, 7-CH_2b), 4.59 (m, 1 H, H-4'), 4.00 (m, 1 H, H-3'), 3.80 (m, 2 H, H-5'a and H-5'b), 2.48 (m, 1 H, H-2'a), 2.37 (m, 1 H, H-2'b), 0.92 (2 s, 18 H, $(CH_3)_3CSi$), 0.11 (s, 6 H, $(CH_3)_2Si$), 0.10 (s, 6 H, $(CH_3)_2Si$).

A solution of *n*-Bu_4NF (17 mg, 0.054 mmol) in THF (1.0 mL) was added to a solution of compound **5** (18 mg, 0.028 mmol) in THF (1.0 mL) at 0°C. The reaction mixture was gradually warmed to room temperature and stirred for two hours. The mixture was concentrated *in vacuo,* dissolved in 1,4-dioxane (2.0 mL), followed by addition of NH_3 in MeOH solution (7 M, 4.0 mL). The mixture was transferred to a sealed tube and stirred at 100°C for 16 hours, then cooled to room temperature, concentrated *in vacuo*, and the residue was purified by silica gel chromatography to yield 7-(2-nitrobenzyloxy)methyl-7-deaza-2'-deoxyadenosine **6** (10 mg, 91%) as a white foam.

^{1}H NMR (400 MHz, DMSO-d_6): δ 8.08 (s, 1 H, H-2), 8.06 (m, 1 H, Ph-H), 7.75 (m, 2 H, Ph-H), 7.58 (m, 1 H, Ph-H), 7.42 (s, 1 H, H-8), 6.64 (bs, 2 H, D_2O exchangeable, 6-NH_2), 6.48 (dd, 1 H, *J* = 2.0 and 6.0 Hz, H-1'), 5.25 (d, 1 H, *J* = 4.0 Hz, D_2O exchangeable, 3'-OH), 5.08 (t, 1 H, *J* = 5.6 Hz, D_2O exchangeable, 5'-OH), 4.90 (s, 2 H, $PhCH_2$), 4.75 (AB dd, 2 H, 7-CH_2), 4.33 (m, 1 H, H-3'), 3.81 (m, 1 H, H-4'), 3.54 (m, 2 H, H-5'a and H-5'b), 2.47 (m, 1 H, H-2'a), 2.15 (m, 1 H, H-2'b).

Compound **6** (6 mg, 0.014mmol) was phosphorylated with $POCl_3$ (2.6 µL, 0.028 mmol) and proton sponge (6 mg, 0.028 mmol) in trimethylphosphate (0.25 mL) at minus 40°C for four hours under a nitrogen atmosphere. A solution of bis-tri-*n*-butylammonium pyrophosphate (66 mg, 0.14 mmol) and tri-*n*-butylamine (28 µL) in anhydrous DMF (0.28 mL) was added. After 30 min of stirring, triethylammonium bicarbonate buffer (1 M, pH 7.5; 1.0 mL) was added. The reaction was stirred at room temperature for one hour and then concentrated *in vacuo*. The

residue was dissolved in water (2.0 mL), filtered, and purified using RP-HPLC (see above) to yield 7-(2-nitro-benzyloxy)methyl-7-deaza-2'-deoxyadenosine-5'-triphosphate **dA.I**.

HRMS (ESI): For the molecular ion $C_{19}H_{23}N_5O_{15}P_3$ $[M-H]^-$, the calculated mass was 654.0403, and the observed mass was 654.0397.

7-[1-(2-nitrophenyl)-2-methyl-propyloxy]methyl-7-deaza-2'-deoxyadenosine-5'-triphosphate

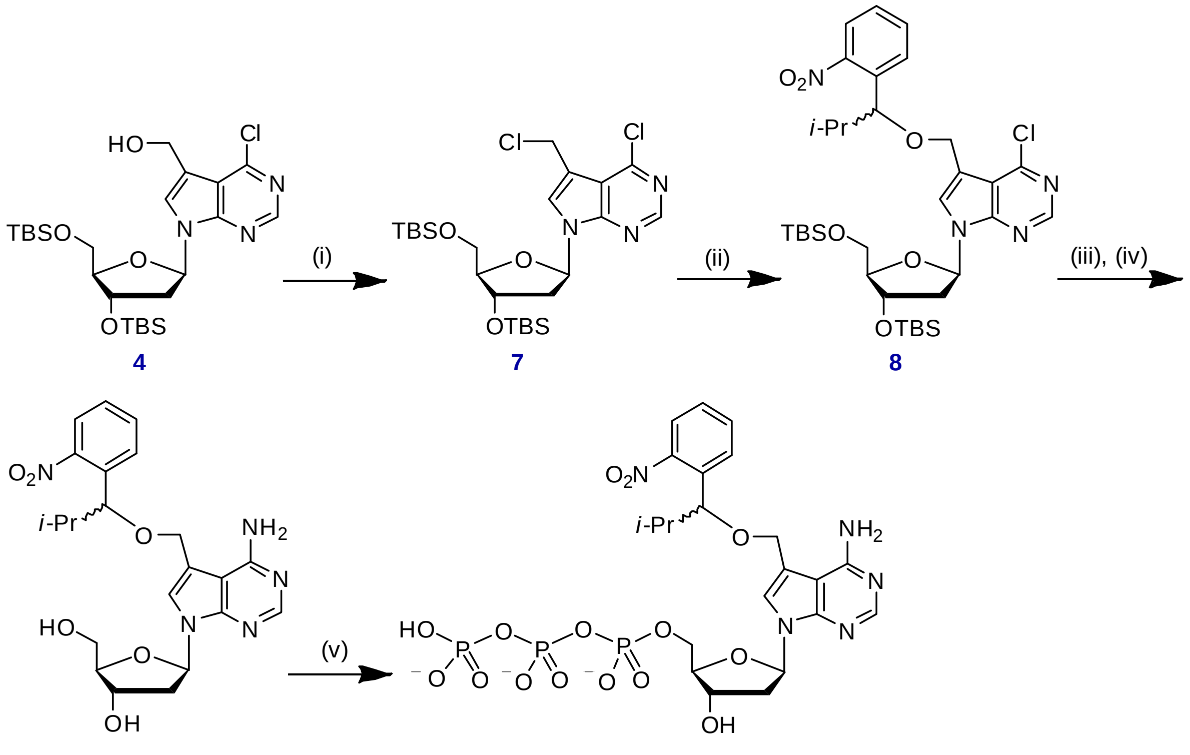

Scheme S7. Synthesis of 7-[1-(2-nitrophenyl)-2-methyl-propyloxy]methyl-7-deaza-2'-deoxyadenosine-5'-triphosphate. *Reagents and conditions*: (*i*) TsCl, DMAP, CH_2Cl_2, room temperature, 39%; (*ii*) racemic (*R*/*S*)-1-(2-nitrophenyl)-2-methyl-propanol, neat, 105°C, 54%; (*iii*) *n*-Bu_4NF, THF, 0°C to room temperature; (*iv*) NH_3, 1,4-dioxane/MeOH, 100°C, 76% for two steps; (*v*) $POCl_3$, $(MeO)_3PO$, minus 40°C to 0°C; $(n\text{-}Bu_3NH)_2H_2P_2O_7$, *n*-$Bu_3N$, DMF; 1 M $HNEt_3HCO_3$.

To a solution of compound **4** (0.26 g, 0.49 mmol) in anhydrous CH_2Cl_2 (12 mL), 4-dimethylaminopyridine (DMAP; 0.15 g, 1.2 mmol) and tosyl chloride (0.11 g, 0.58 mmol) were added. The reaction mixture was stirred at room temperature for 18 hours and then concentrated *in vacuo*. The residue was purified by silica gel chromatography to yield 9-[β-D-3',5'-*O*-bis-(*tert*-butyldimethylsilyl)-2'-deoxyribofuranosyl]-6-chloro-7-chloromethyl-7-deazapurine **7** (0.103 g, 39%) as a viscous oil.

^{1}H NMR (400 MHz, $CDCl_3$): δ 8.64 (s, 1 H, H-2), 7.72 (s, 1 H, H-8), 6.73 (t, 1 H, *J* = 6.8 Hz, H-1'), 4.95 (AB d, *J* = 12.4 Hz, 7-CH_{2a}), 4.91 (AB d, *J* = 12.0 Hz, 7-CH_{2b}), 4.58 (m, 1 H, H-3'), 4.00 (m, 1 H, H-4'), 3.82 (m, 2 H, H-5'a and H-5'b), 2.41 (m, 2 H, H-2'a and H-2'b), 0.95 (s, 9 H, $(CH_3)_3CSi$), 0.93 (s, 9 H, $(CH_3)_3CSi$), 0.12 (s, 6 H, $(CH_3)_2Si$), 0.11 (s, 6 H, $(CH_3)_2Si$).

Compound **7** (54 mg, 0.10 mmol) and racemic (*R*/*S*)-1-(2-nitrophenyl)-2-methyl-propanol (191 mg, 0.98 mmol) were dissolved in anhydrous CH_2Cl_2 (10 mL). The solvent was removed *in vacuo*, and the residue was heated for one hour under a nitrogen atmosphere, then dissolved in minimum amount of ethyl acetate and purified by silica gel chromatography to yield 9-[β-D-3',5'-*O*-bis-(*tert*-butyldimethylsilyl)-2'-deoxyribofuranosyl]-6-chloro-7-[1-(2-nitro-phenyl)-2-methyl-propyloxy]methyl-7-deazapurine **8** (38 mg, 54%) as a 1:1 mixture of two diastereomers.

^{1}H NMR (400 MHz, $CDCl_3$) for diastereomers: δ 8.60 and 8.59 (2 s, 1 H, H-2), 7.83 (m, 1 H, Ph-H), 7.79 (m, 1 H, Ph-H), 7.56 (m, 1 H, Ph-H), 7.48 and 7.47 (2 s, 1 H, H-8), 7.38 (m, 1 H, Ph-H), 6.70 (m, 1 H, H-1'), 4.81 (m, 1 H, Ph-CH), 4.70 (m, 1H, 7-CH_2a), 4.58 (m, 2 H, 7-CH_2b and H-3'), 3.99 (m, 1 H, H-4'), 3.78 (m, 2 H, H-5'a and H-5'b), 2.48 (m, 1 H, H-2'a), 2.35 (m, 1 H, H-2'b), 1.96 (m, 1 H, CH), 0.98 and 0.96 (2 d, 3 H, CH_3), 0.93 (2 s, 9 H, $(CH_3)_3CSi$), 0.89 (2 s, 9 H, $(CH_3)_3CSi$), 0.82 and 0.78 (2 d, 3 H, CH_3), 0.12 (2 s, 6 H, $(CH_3)_2Si$), 0.08 and 0.07 (2 s, 3 H, $(CH_3)_2Si$), 0.06 and 0.05 (2 s, 3 H, $(CH_3)_2Si$).

A solution of *n*-Bu_4NF (44 mg, 0.14 mmol) in THF (2.0 mL) was added to a solution of compound **8** (38 mg, 0.05 mmol) in THF (2.0 mL) at 0°C. The reaction was gradually warmed to room temperature and stirred for two hours. The mixture was concentrated *in vacuo,* dissolved in 1,4-dioxane (4.0 mL), followed by addition of NH_3 in MeOH solution (7 M, 8.0 mL). The mixture was transferred to a sealed tube, stirred at 100°C for 24 hours, cooled to room temperature, and then concentrated *in vacuo*. The residue was purified by silica gel chromatography to yield 7-[1-(2-nitrophenyl)-2-methyl-propyloxy]methyl-7-deaza-2'-deoxyadenosine **9** (19 mg, 76%) as a 1:1 mixture of two diastereomers.

^{1}H NMR (400 MHz, DMSO-d_6) for diastereomers: δ 8.06 and 8.04 (2 s, 1 H, H-2), 7.90 (m, 1 H, Ph-H), 7.67 (m, 2 H, Ph-H), 7.56 (m, 2 H, Ph-H), 7.19 and 7.16 (2 s, 1 H, H-8), 6.63 (bs, 2 H, D_2O exchangeable, 6-NH_2), 6.39 (m, 1 H, H-1'), 5.23 (m, 1 H, D_2O exchangeable, 3'-OH), 5.00 (m, 1 H, D_2O exchangeable, 5'-OH), 4.72 (2 d, 1 H, Ph-CH), 4.45 (s, 2 H, 7-CH_2), 4.30 (m, 1 H, H-3'), 3.77 (m, 1 H, H-4'), 3.49 (m, 2 H, H-5'a and H-5'b), 2.40 (m, 1 H, H-2'a), 2.12 (m, 1 H, H-2'b), 1.94 (m, 1 H, CH), 0.87 (m, 3 H, CH_3), 0.74 (m, 3 H, CH_3).

Compound **9** (19 mg, 0.041 mmol) was phosphorylated with $POCl_3$ (16 µL, 0.16 mmol) and proton sponge (18 mg, 0.082 mmol) in trimethylphosphate (0.4 mL) at minus 40°C for five hours under a nitrogen atmosphere. A solution of bis-tri-*n*-butylammonium pyrophosphate (97 mg, 0.20 mmol) and tri-*n*-butylamine (40 µL) in anhydrous DMF (0.40 mL) was added. After 30 min of stirring, triethylammonium bicarbonate buffer (1 M, pH 7.5; 10 mL) was added. The reaction was stirred at room temperature for one hour and then concentrated *in vacuo*. The residue was dissolved in water (5.0 mL), filtered, and purified by anion exchange chromatography. The fractions containing triphosphate were combined and lyophilized to yield 7-[1-(2-nitrophenyl)-2-methyl-propyloxy]methyl-7-deaza-2'-deoxyadenosine-5'-triphosphate **dA.III.a** as a 1:1 mixture of two diastereomers, which were separated using RP-HPLC to yield the single diastereomers **dA.III.a ds1** and **dA.III.a ds2**.[1]

HRMS (ESI): For the molecular ion $C_{22}H_{29}N_5O_{15}P_3$ [M-H]$^-$, the calculated mass was 696.0873, and the observed mass was 696.0864.

[1] In all cases, diastereomer 1 (ds1) eluted faster than diastereomer 2 (ds2) by RP-HPLC.

7-[1-(4-Methoxy-2-nitrophenyl)-2-methyl-propyloxy]methyl-7-deaza-2'-deoxyadenosine-5'-triphosphate

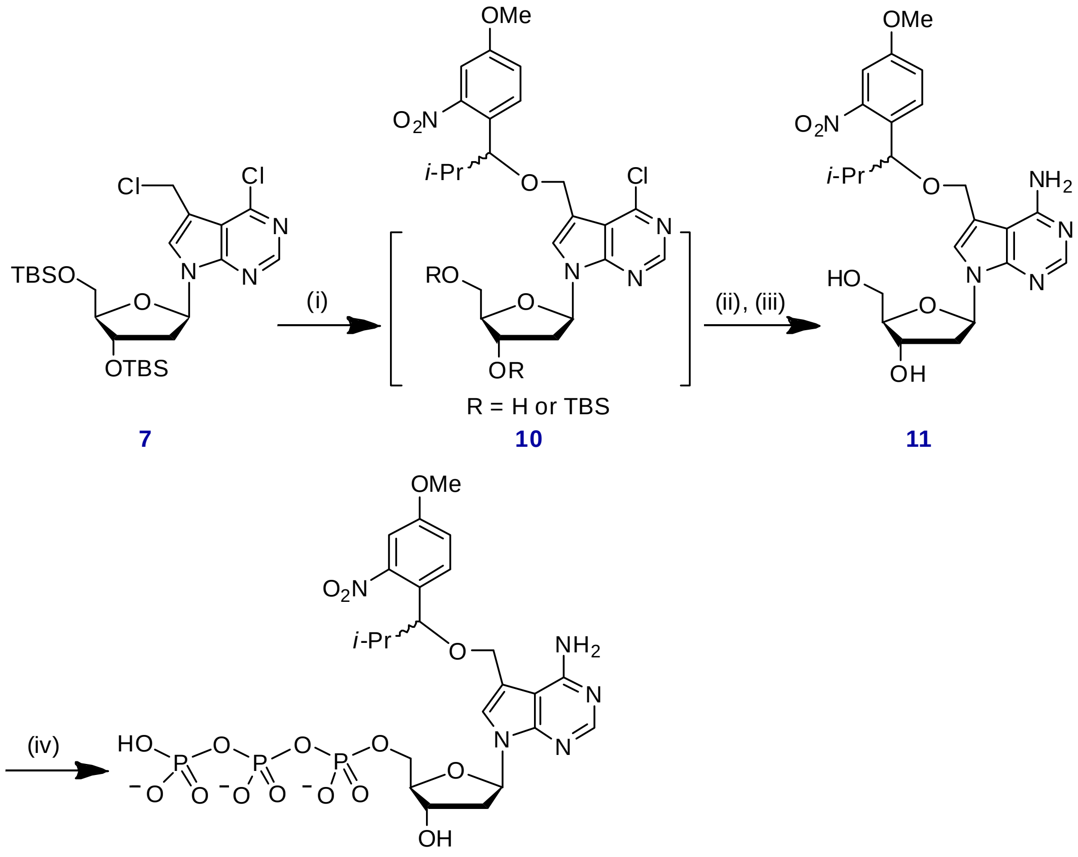

Scheme S8. Synthesis of 7-[1-(4-methoxy-2-nitrophenyl)-2-methyl-propyloxy]methyl-7-deaza-2'-deoxyadenosine-5'-triphosphate. *Reagents and conditions*: (*i*) racemic (*R*/*S*)-1-(4-methoxy-2-nitrophenyl)-2-methyl-1-propanol, 108°C; (*ii*) *n*-Bu_4NF, THF, 0°C to room temperature; (*iii*) NH_3, 1,4-dioxane/MeOH, 100°C, 32% for three steps; (*iv*) $POCl_3$, $(MeO)_3PO$, 0°C; $(n\text{-}Bu_3NH)_2H_2P_2O_7$, *n*-$Bu_3N$, DMF; 1 M $HNEt_3HCO_3$.

Compound **7** (103 mg, 0.19 mmol) and racemic (*R*/*S*)-1-(4-methoxy-2-nitrophenyl)-2-methyl-1-propanol (428 mg, 1.9 mmol) were dissolved in anhydrous CH_2Cl_2 (3.0 mL). The solvent was removed *in vacuo*, and the residue was heated at 108°C for 30 min under a nitrogen atmosphere, cooled to room temperature, dissolved in minimum amount of ethyl acetate, and purified by silica gel chromatography to yield 6-chloro-7-[1-(4-methoxy-2-nitrophenyl)-2-methyl-propyloxy]methyl-7-deazapurine 2'-deoxyribonucleosides **10**. The sample was dissolved in THF (8.0 mL), cooled to 0°C, and then added to a solution of *n*-Bu_4NF (68 mg, 0.22 mmol) in THF (2.0 mL). The reaction was gradually warmed to room temperature

and stirred for 30 min. The mixture was concentrated *in vacuo,* dissolved in 1,4-dioxane (8.0 mL), followed by addition of NH_3 in MeOH (7 N, 24 mL). The mixture was transferred to a sealed tube and stirred at 100°C for 16 hours, then cooled to room temperature, and concentrated *in vacuo*. The residue was purified by silica gel chromatography to yield 7-[1-(4-methoxy-2-nitrophenyl)-2-methyl-propyloxy]methyl-7-deaza-2'-deoxyadenosine **11** (30 mg, 32% for three steps) as a 1:1 mixture of two diastereomers.

^{1}H NMR (400 MHz, DMSO-d_6) for diastereomers: δ 8.06 and 8.05 (2 s, 1 H, H-2), 7.57 and 7.54 (2 d, 1 H, *J* = 8.8 Hz, Ph-H), 7.47 and 7.44 (2 d, 1 H, *J* = 2.6 Hz, Ph-H), 7.33 and 7.27 (2 dd, *J* = 8.8 and 2.6 Hz, 1 H, Ph-H), 7.18 and 7.15 (2 s, 1 H, H-8), 6.63 (bs, 2 H, D_2O exchangeable, 6-NH_2), 6.43 (m, 1 H, H-1'), 5.24 (m, 1 H, D_2O exchangeable, 3'-OH), 5.03 (m, 1 H, D_2O exchangeable, 5'-OH), 4.55 (m, 2 H, Ph-CH, 7-CH_2a), 4.30 (m, 2 H, 7-CH_2b and H-3'), 3.86 and 3.84 (2 s, 3 H, MeO), 3.78 (m, 1 H, H-4'), 3.48 (m, 2 H, H-5'), 2.45 (m, 1 H, H-2'a), 2.12 (m, 1 H, H-2'b), 1.93 (m, 1 H, $CH(CH_3)_2$), 0.88 (m, 3 H, CH_3), 0.74 and 0.71 (2 d, *J* = 6.8 Hz, 3 H, CH_3).

Compound **11** (28 mg, 0.06 mmol) was phosphorylated with $POCl_3$ (11 µL, 0.12 mmol) and proton sponge (25 mg, 0.12 mmol) in trimethylphosphate (0.35 mL) at 0°C for two hours under a nitrogen atmosphere. A solution of bis-tri-*n*-butylammonium pyrophosphate (237 mg, 0.50 mmol) and tri-*n*-butylamine (100 µL) in anhydrous DMF (1.0 mL) was added. After 10 min of stirring, triethylammonium bicarbonate buffer (1 M, pH 7.5; 10 mL) was added. The reaction was stirred at room temperature for one hour and then concentrated *in vacuo*. The residue was dissolved in 20% aqueous acetonitrile (10 mL), filtered, and purified by anion exchange chromatography. The fractions containing triphosphate were combined and lyophilized to yield 7-[1-(4-methoxy-2-nitrophenyl)-2-methyl-propyloxy]methyl-7-deaza-2'-deoxyadenosine-5'-triphosphate **dA.III.b** as a 1:1 mixture of two diastereomers, which were separated using RP-HPLC to yield the single diastereomers **dA.III.b ds1** and **dA.III.b ds2**.

HRMS (ESI): For the molecular ion $C_{23}H_{31}N_5O_{16}P_3$ $[M-H]^-$, the calculated mass was 726.0979, and the observed mass was 726.0984.

7-[1-(2,6-Dinitrophenyl)-2-methyl-propyloxy]methyl-7-deaza-2'-deoxyadenosine-5'-triphosphate

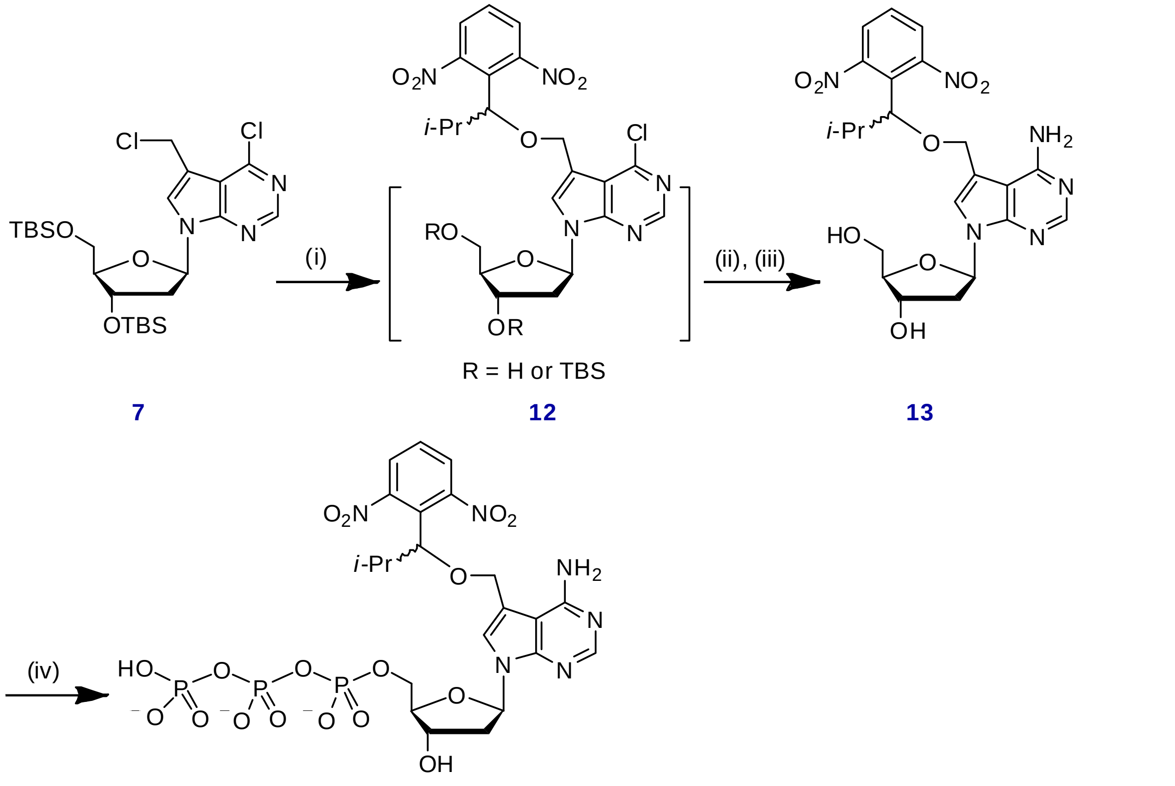

dA.III.c

Scheme S9. Synthesis of 7-[1-(2,6-dinitrophenyl)-2-methyl-propyloxy]methyl-7-deaza-2'-deoxy-adenosine-5'-triphosphate. *Reagents and conditions*: (*i*) racemic (*R*/*S*)-1-(2,6-dinitrophenyl)-2-methyl-1-propanol, 108°C; (*ii*) *n*-Bu_4NF, THF, 0°C to room temperature; (*iii*) NH_3, 1,4-dioxane/MeOH, 100°C, 38% for three steps; (*iv*) $POCl_3$, $(MeO)_3PO$, 0°C; $(n\text{-}Bu_3NH)_2H_2P_2O_7$, *n*-$Bu_3N$, DMF; 1 M $HNEt_3HCO_3$.

Compound **7** (109 mg, 0.20 mmol) and racemic (*R*/*S*)-1-(2,6-dinitrophenyl)-2-methyl-1-propanol (448 mg, 1.9 mmol) were dissolved in anhydrous CH_2Cl_2 (10 mL). The solvent was removed *in vacuo*, and the residue was heated at 108°C for 30 min under a nitrogen atmosphere, then dissolved in minimum amount of ethyl acetate and purified by silica gel chromatography to yield 6-chloro-7-[1-(2,6-dinitrophenyl)-2-methyl-propyloxy]methyl-7-deazapurine-2'-deoxyribo-nucleosides **12**. The sample was dissolved in THF (5.0 mL), cooled to 0°C, and then added a solution of *n*-Bu_4NF (31 mg, 0.10 mmol) in THF (2.0 mL). The reaction was gradually warmed to room temperature and stirred for two hours. The mixture was concentrated *in vacuo,* dissolved in 1,4-dioxane (4.0 mL), followed by addition of NH_3 in MeOH (7 N, 18 mL). The mixture was transferred to a sealed tube, stirred at 100°C for 36 hours, cooled to room temperature, and then concentrated *in vacuo*. The residue was purified by silica gel chromatography to yield

7-[1-(2,6-dinitrophenyl)-2-methyl-propyloxy]methyl-7-deaza-2’-deoxyadenosine **13** (38 mg, 38% for three steps) as a 1:1 mixture of two diastereomers.

^{1}H NMR (400 MHz, DMSO-d_6) for diastereomers: δ 8.17 (m, 1 H, Ph-H), 8.07 and 8.06 (2 s, 1 H, H-2), 7.85 (m, 1 H, Ph-H), 7.69 (m, 1 H, Ph-H), 7.20 and 7.18 (2 s, 1 H, H-8), 6.57 (bs, 2 H, D_2O exchangeable, 6-NH_2), 6.46 (m, 1 H, H-1’), 5.26 (d, *J* = 3.6 Hz, 1 H, D_2O exchangeable, 3’-OH), 5.01 (m, 1 H, D_2O exchangeable, 5’-OH), 4.60 (m, 2 H, Ph-CH and 7-CH_2a), 4.29 (m, 1 H, 7-CH_2b), 4.13 (m, 1 H, H-3’), 3.80 (m, 1 H, H-4’), 3.51 (m, 2 H, H-5’a and H-5’b), 2.49 (m, 1 H, $CH(CH_3)_3$), 2.16 (m, 2 H, H-2’a and H-2’b), 0.91 (m, 3 H, CH_3), 0.65 (m, 3 H, CH_3).

ToF-MS (ESI): For the molecular ion $C_{22}H_{27}N_6O_8$ $[M+H]^+$, the calculated mass was 503.1890, and the observed mass was 503.2029.

Compound **13** (30 mg, 0.06 mmol) was phosphorylated with $POCl_3$ (17 µL, 0.18 mmol) and proton sponge (26 mg, 0.12 mmol) in trimethylphosphate (0.4 mL) at 0°C for four hours under a nitrogen atmosphere. A solution of bis-tri-*n*-butylammonium pyrophosphate (285 mg, 0.6 mmol) and tri-*n*-butylamine (120 µL) in anhydrous DMF (1.2 mL) was added. After 30 min of stirring, triethylammonium bicarbonate buffer (1 M, pH 7.5; 10 mL) was added. The reaction was stirred for one hour at room temperature and then concentrated *in vacuo*. The residue was dissolved in 20% aqueous acetonitrile (10 mL), filtered, and purified by anion exchange chromatography. The fractions containing triphosphate were combined and lyophilized to yield 7-[1-(2,6-dinitrophenyl)-2-methyl-propyloxy]methyl-7-deaza-2’-deoxyadenosine-5’-triphosphate **dA.III.c** as a 1:1 mixture of two diastereomers which were separated using RP-HPLC to yield the single diastereomers **dA.III.c ds1** and **dA.III.c ds2**.

HRMS (ESI): For the molecular ion $C_{22}H_{28}N_6O_{17}P_3$ $[M-H]^-$, the calculated mass was 741.0724, and the observed mass was 741.0731.

7-[(S)-1-(2-Nitrophenyl)-2,2-dimethyl-propyloxy]methyl-7-deaza-2’-deoxyadenosine-5’-triphosphate

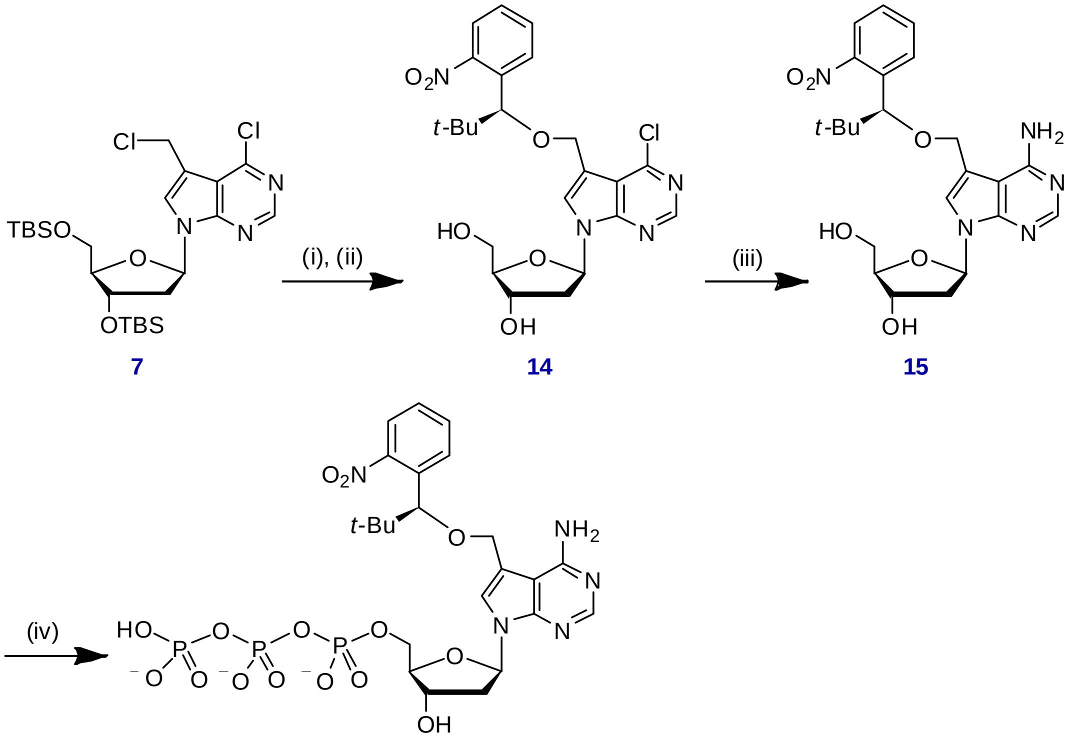

dA.V

Scheme S10. Synthesis of 7-[(*S*)-1-(2-nitrophenyl)-2,2-dimethyl-propyloxy]methyl-7-deaza-2’-deoxyadenosine-5’-triphosphate. *Reagents and conditions*: (*i*) (*S*)-1-(2-nitrophenyl)-2,2-dimethyl-1-propanol, 110°C; (*ii*) *n*-Bu_4NF, THF, room temperature; 75% for two steps; (*iii*) NH_3, 1,4-dioxane/MeOH, 100°C, 93%; (*iv*) $POCl_3$, $(MeO)_3PO$, 0°C; $(n\text{-}Bu_3NH)_2H_2P_2O_7$, *n*-$Bu_3N$, DMF; 1 M $HNEt_3HCO_3$.

Compound **7** (130 mg, 0.24 mmol) and (*S*)-1-(2-nitrophenyl)-2,2-dimethyl-1-propanol (290 mg, 1.4 mmol) were heated at 110°C for 45 min under a nitrogen atmosphere. The reaction mixture was cooled to room temperature and dissolved in THF (10 mL) followed by addition of *n*-Bu_4NF (189 mg, 0.60 mmol). The mixture was stirred at room temperature for two hours and then concentrated *in vacuo*. The residue was dissolved in CH_2Cl_2 (20 mL) and washed with brine (30 mL), and the aqueous phase was extracted with CH_2Cl_2 (20 mL) two times. The combined organic phase was dried over Na_2SO_4, concentrated *in vacuo*, and the residue was purified by silica gel chromatography to yield 6-chloro-9-[β-D-2’-deoxyribofuranosyl]-7-[(*S*)-1-(2-nitrophenyl)-2,2-dimethyl-propyloxy]methyl-7-deazapurine **14** (90 mg, 75%).

^{1}H NMR (400 MHz, $CDCl_3$): δ 8.51 (s, 1 H, H-2), 7.68 (m, 2 H, Ph-H), 7.45 (t, 1 H, *J* = 7.2 Hz, Ph-H), 7.38 (s, 1 H, H-8), 7.29 (t, 1 H, *J* = 7.2 Hz, Ph-H), 6.39 (dd, 1 H, *J* = 6.0 and 8.0 Hz, H-1'), 4.97 (s, 1 H, Ph-CH), 4.70 (m, 3 H, 7-CH_2 and H-3'), 4.16 (m, 1 H, H-4'), 3.83 (m, 2 H, H-5'), 2.80 (m, 1 H, H-2'a), 2.35 (m, 1 H, H-2'b), 0.82 (s, 9 H, $C(CH_3)_3$).

Compound **14** (90 mg, 0.18 mmol) was dissolved in 1,4-dioxane (8.0 mL) followed by addition of NH_3 in MeOH (7 N, 16 mL). The mixture was transferred to a sealed tube and stirred at 100°C for 24 hours, cooled to room temperature, and then concentrated *in vacuo*. The residue was purified by silica gel chromatography to yield 7-[(*S*)-1-(2-nitrophenyl)-2,2-dimethyl-propyloxy]methyl-7-deaza-2'-deoxyadenosine **15** (80 mg, 93%).

^{1}H NMR (400 MHz, DMSO-d_6): δ 8.09 (s, 1 H, H-2), 7.91 (dd, 1 H, *J* = 1.2 and 8.0 Hz, Ph-H), 7.71 (m, 2 H, Ph-H), 7.58 (m, 1 H, Ph-H), 7.24 (s, 1 H, H-8), 6.68 (bs, 2 H, D_2O exchangeable, 6-NH_2), 6.46 (dd, 1 H, *J* = 6.0 and 8.0 Hz, H-1'), 5.27 (d, 1 H, D_2O exchangeable, 3'-OH), 5.06 (t, 1 H, D_2O exchangeable, 5'-OH), 4.87 (s, 1 H, Ph-CH), 4.65 (d, 1 H, *J* = 12.8 Hz, 7-CH_2a), 4.49 (m, 1 H, H-3'), 4.36 (d, 1 H, 7-CH_2b), 3.80 (m, 1 H, H-4'), 3.49 (m, 2 H, H-5'), 2.45 (m, 1 H, H-2'a), 2.17 (m, 1 H, H-2'b), 0.75 (s, 9 H, $C(CH_3)_3$).

Compound **15** (25 mg, 0.053 mmol) was phosphorylated with $POCl_3$ (22 µL, 0.24 mmol) and proton sponge (23 mg, 0.11 mmol) in trimethylphosphate (0.35 mL) at 0°C for 4.5 hours under a nitrogen atmosphere. A solution of bis-tri-*n*-butylammonium pyrophosphate (237 mg, 0.50 mmol) and tri-*n*-butylamine (100 µL) in anhydrous DMF (1.0 mL) was added. After 10 min of stirring, triethylammonium bicarbonate buffer (1 M, pH 7.5; 10 mL) was added. The reaction was stirred at room temperature for one hour and then concentrated *in vacuo*. The residue was dissolved in 20% aqueous acetonitrile (20 mL), filtered, and purified by anion exchange chromatography. The fractions containing triphosphate were combined and lyophilized to yield 7-[(*S*)-1-(2-nitrophenyl)-2,2-dimethyl-propyloxy]methyl-7-deaza-2'-deoxyadenosine-5'-triphosphate **dA.V**, which was further purified using RP-HPLC.

HRMS (ESI): For the molecular ion $C_{23}H_{31}N_5O_{15}P_3$ $[M-H]^-$, the calculated mass was 710.1029, and the observed mass was 710.1032.

7-[(S)-1-(5-Methoxy-2-nitrophenyl)-2,2-dimethyl-propyloxy]methyl-7-deaza-2'-deoxyadenosine-5'-triphosphate

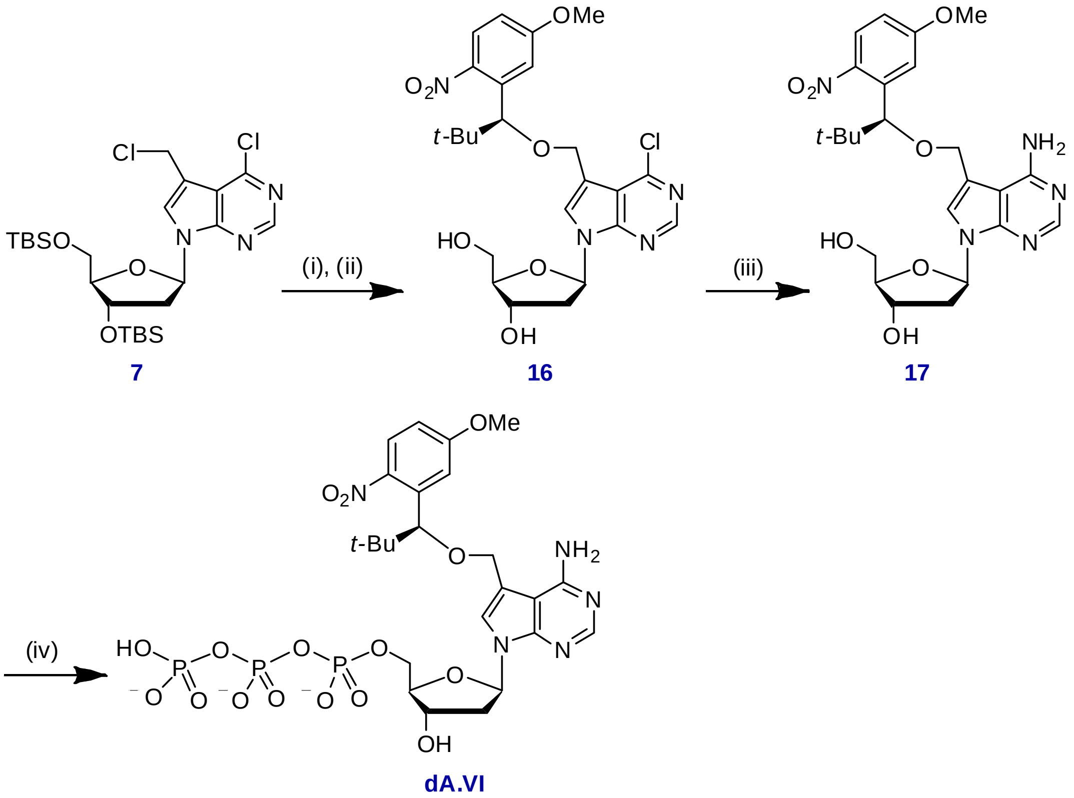

Scheme S11. Synthesis of 7-[(*S*)-1-(5-methoxy-2-nitrophenyl)-2,2-dimethyl-propyloxy]methyl-7-deaza-2'-deoxyadenosine-5'-triphosphate. *Reagents and conditions*: (*i*) (*S*)-1-(5-methoxy-2-nitrophenyl)-2,2-dimethyl-1-propanol, 110°C; (*ii*) *n*-Bu_4NF, THF, room temperature; 78% for two steps; (*iii*) NH_3, 1,4-dioxane/MeOH, 100°C, 74%; (*iv*) $POCl_3$, $(MeO)_3PO$, 0°C; $(n\text{-}Bu_3NH)_2H_2P_2O_7$, *n*-$Bu_3N$, DMF; 1 M $HNEt_3HCO_3$.

Compound **7** (165 mg, 0.30 mmol) and (*S*)-1-(5-methoxy-2-nitrophenyl)-2,2-dimethyl-1-propanol (330 mg, 1.4 mmol) were heated at 110°C for 45 min under a nitrogen atmosphere. The reaction mixture was cooled to room temperature and dissolved in THF (10 mL), followed by addition of *n*-Bu_4NF (236 mg, 0.75 mmol). The mixture was stirred at room temperature for two hours and then concentrated *in vacuo*. The residue was dissolved in CH_2Cl_2 (40 mL) and washed with brine (50 mL), and the aqueous phase was extracted with CH_2Cl_2 (40 mL) two times. The combined organic phase was dried over Na_2SO_4, concentrated *in vacuo*, and the residue was purified by silica gel chromatography to yield 6-chloro-9-[β-D-2'-deoxyribofuranosyl]-7-[(*S*)-1-(5-methoxy-2-nitrophenyl)-2,2-dimethyl-propyloxy]methyl-7-deazapurine **16** (122 mg, 78%).

^{1}H NMR (400 MHz, $CDCl_3$): δ 8.55 (s, 1 H, H-2), 7.79 (d, 1 H, *J* = 9.2 Hz, Ph-H), 7.35 (s, 1 H, H-8), 7.15 (d, 1 H, *J* = 3.2 Hz, Ph-H), 6.68 (dd, 1 H, *J* = 3.2 and 9.2 Hz, Ph-H), 6.33 (dd, 1 H, *J* = 5.6 and 8.8 Hz, H-1'), 5.26 (s, 1 H, Ph-CH), 4.85 (d, 1 H, *J* = 8.8 Hz, 7-CH_2a), 4.75 (m, 1 H, H-3'), 4.70 (d, 1 H, *J* = 8.8 Hz, 7-CH_2b), 4.13 (m, 1 H, H-4'), 3.95 (m, 1 H, H-5'a), 3.83 (s, 3 H, OCH_3), 3.78 (m, 1 H, H-5'b), 2.86 (m, 1 H, H-2'a), 2.30 (m, 1 H, H-2'b), 0.83 (s, 9 H, $C(CH_3)_3$).

Compound **16** (120 mg, 0.23 mmol) was dissolved in 1,4-dioxane (10 mL) followed by addition of NH_3 in MeOH (7 N, 10 mL). The mixture was transferred to a sealed tube and stirred at 100°C for 24 hours, then cooled to room temperature, concentrated *in vacuo*, and the residue was purified by silica gel chromatography to yield 7-[(*S*)-1-(5-methoxy-2-nitrophenyl)-2,2-dimethyl-propyloxy]methyl-7-deaza-2'-deoxyadenosine **17** (87 mg, 74%).

^{1}H NMR (400 MHz, DMSO-d_6): δ 8.06 (s, 1 H, H-2), 7.97 (d, 1 H, *J* = 9.2 Hz, Ph-H), 7.22 (s, 1 H, H-8), 7.08 (d, 1 H, *J* = 2.8 Hz, Ph-H), 7.05 (dd, 1 H, *J* = 2.8 and 9.2 Hz, Ph-H), 6.66 (bs, 2 H, D_2O exchangeable, 6-NH_2), 6.42 (dd, 1 H, *J* = 6.0 and 8.0 Hz, H-1'), 5.25 (d, 1 H, D_2O exchangeable, 3'-OH), 5.15 (s, 1 H, Ph-CH), 5.03 (t, 1 H, D_2O exchangeable, 5'-OH), 4.64 (d, 1 H, *J* = 12.8 Hz, 7-CH_2a), 4.43 (d, 1 H, *J* = 12.8 Hz, 7-CH_2b), 4.30 (m, 1 H, H-3'), 3.84 (s, 3 H, OCH_3), 3.77 (m, 1 H, H-4'), 3.45 (m, 2 H, H-5'), 2.43 (m, 1 H, H-2'a), 2.14 (m, 1 H, H-2'b), 0.75 (s, 9 H, $C(CH_3)_3$).

Compound **17** (21 mg, 0.042 mmol) was phosphorylated with $POCl_3$ (40 µL, 0.43 mmol) and proton sponge (18 mg, 0.084 mmol) in trimethylphosphate (0.35 mL) at 0°C for 7.5 hours under a nitrogen atmosphere. A solution of bis-tri-*n*-butylammonium pyrophosphate (237 mg, 0.50 mmol) and tri-*n*-butylamine (100 µL) in anhydrous DMF (1.0 mL) was added. After 10 min of stirring, triethylammonium bicarbonate buffer (0.1 M, pH 7.5; 10 mL) was added. The reaction was stirred at room temperature for one hour and then concentrated *in vacuo*. The residue was dissolved in 20% aqueous acetonitrile (20 mL), filtered, and purified by anion exchange chromatography. The fractions containing triphosphate were combined and lyophilized to yield 7-[(*S*)-1-(5-methoxy-2-nitrophenyl)-2,2-dimethyl-propyloxy]methyl-7-deaza-2'-deoxyadenosine-5'-triphosphate **dA.VI**, which was further purified using RP-HPLC.

HRMS (ESI): For the molecular ion $C_{24}H_{33}N_5O_{16}P_3$ $[M-H]^-$, the calculated mass was 740.1135, and the observed mass was 740.1156.

VI. Synthesis of 7-HOMe-7-deaza-2’-deoxyguanosine triphosphate analogs

7-(2-nitrobenzyloxy)methyl-7-deaza-2’-deoxyguanosine-5’-triphosphate

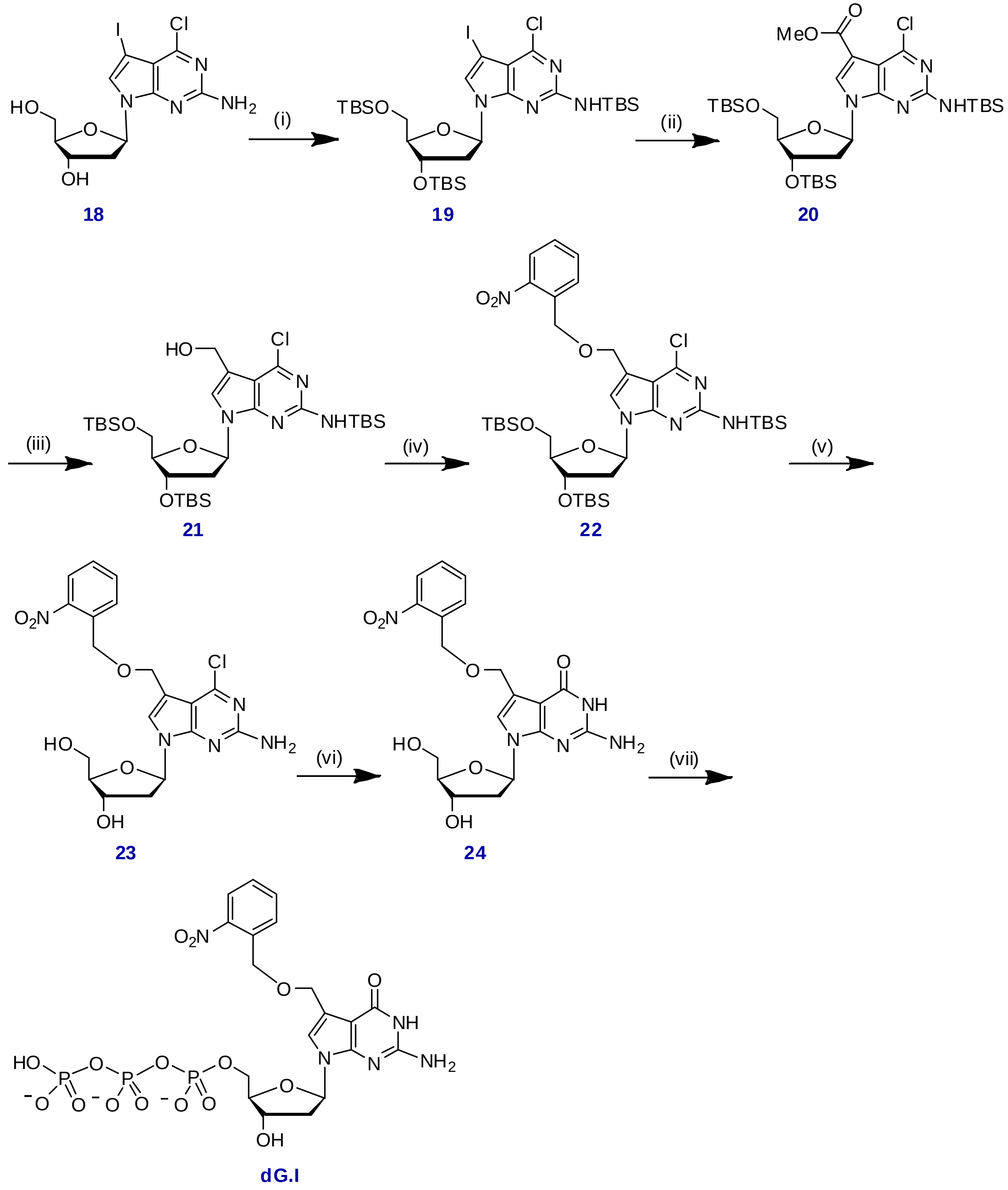

Scheme S12. Synthesis of 7-(2-nitrobenzyloxy)methyl-7-deaza-2’-deoxyguanosine-5’-triphosphate. *Reagents and conditions*: (*i*) TBSCl, imidiazole, DMF, room temperature, 60%; (*ii*) CO, $PdCl_2[PhCN]_2$, MeOH/1,4-dioxane, 50°C, 91%; (*iii*) $LiBH_4$, MeOH, THF, reflux, 54%; (*iv*) 2-nitrobenzyl bromide,

n-Bu_4NBr, CH_2Cl_2/aq. NaOH, room temperature, 48%; (*v*) *n*-Bu_4NF, THF, 0°C to room temperature, 95%; (*vi*) DABCO, H_2O, reflux, 30%; (*vii*) $POCl_3$, proton sponge, $(MeO)_3PO$, 0^oC; (*n*-$Bu_3NH)_2H_2P_2O_7$, *n*-Bu_3N, DMF; 1 M $HNEt_3HCO_3$.

Compound **18**[5] (1.35 g, 3.29 mmol) was evaporated from anhydrous pyridine (3.0 mL) three times and then dissolved in anhydrous DMF (6.0 mL). *tert*-Butyldimethylsilyl chloride (5.95 g, 39.5 mmol) and imidazole (5.37 g, 78.9 mmol) were added, and the mixture was stirred at 50°C for 48 hours with additional *tert*-butyldimethylsilyl chloride (2.97 g, 19.7 mmol) and imidazole (2.69 g, 39.4 mmol) being added every six hours. The reaction mixture was concentrated *in vacuo* and purified by silica gel chromatography to yield 9-[β-D-3',5'-*O*-bis-(*tert*-butyldimethylsilyl)-2'-deoxyribofuranosyl]-2-(*tert*-butyldimethylsilyl)amino-6-chloro-7-iodo-7-deazapurine **19** (1.48 g, 60% yield) as a white foam.

^1H NMR (400 MHz, $CDCl_3$): δ 7.35 (s, 1 H, H-8), 6.53 (t, 1 H, *J* = 6.0 Hz, H-1'), 4.70 (s, 1 H, 2-NH), 4.47 (m, 1 H, H-3'), 3.97 (m, 1 H, H-4'), 3.78 (m, 2 H, H-5'a and H-5'b), 2.23 (m, 2 H, H-2'a and H-2'b), 0.98 (s, 9 H, $(CH_3)_3CSi$), 0.95 (s, 9 H, $(CH_3)_3CSi$), 0.90 (s, 9 H, $(CH_3)_3CSi$), 0.29 (2 s, 6 H, $(CH_3)_2Si$), 0.13 (2 s, 6 H, $CH_3)_2Si$), 0.09 (s, 6 H, $CH_3)_2Si$).

A solution of compound **19** (720 mg, 0.96 mmol) was dissolved in anhydrous 1,4-dioxane (30 mL). Anhydrous MeOH (30 mL) and triethylamine (0.58 mL) were added, and the mixture was stirred for 10 min under a CO atmosphere, followed by addition of bis(benzonitrile)dichloropalladium(II) (20 mg, 0.05 mmol). The reaction was stirred at 58^oC for 24 hours under a CO atmosphere, and then concentrated *in vacuo.* The residue was purified by silica gel chromatography to yield 9-[β-D-3',5'-*O*-bis-(*tert*-butyldimethylsilyl)-2'-deoxyribofuranosyl]-2-(*tert*-butyldimethylsilyl)amino-6-chloro-7-methoxycarbonyl-7-deazapurine **20** (600 mg, 91%) as a viscous oil.

^1H NMR (400 MHz, $CDCl_3$): δ 7.92 (s, 1 H, H-8), 6.57 (dd, 1 H, *J* = 8.0 and 6.0 Hz, H-1'), 4.78 (s, 1 H, 2-NH), 4.49 (m, 1 H, H-3'), 4.02 (m, 1 H, H-4'), 3.85 (s, 3 H, CH_3), 3.81 (m, 2 H, H-5'a and H-5'b), 2.25 (m, 2 H, H-2'a and H-2'b), 0.98 (s, 9 H, $(CH_3)_3CSi$), 0.93 (s, 9 H, $(CH_3)_3CSi$), 0.92 (s, 9 H, $(CH_3)_3CSi$), 0.31 (s, 6 H, $(CH_3)_2Si$), 0.13 (2 s, 6 H, $(CH_3)_2Si$), 0.11 (s, 6 H, $(CH_3)_2Si$).

To a solution of compound **20** (1.11 g, 1.63 mmol) in anhydrous THF (56 mL), lithium borohydride (143 mg, 6.5 mmol) was added, followed by MeOH (0.94 mL). The reaction mixture was heated to reflux for one hour. Upon cooling to room temperature, the reaction mixture was diluted with CH_2Cl_2 (700 mL) and quenched with water (70 mL). The organic phase

was separated, dried over Na_2SO_4, and concentrated *in* vacuo. The residue was purified by silica gel chromatography to yield 9-[β-D-3',5'-*O*-bis-(*tert*-butyldimethylsilyl)-2'-deoxy-ribofuranosyl]-2-(*tert*-butyldimethylsilyl)amino-6-chloro-7-hydroxymethyl-7-deazapurine **21** (0.58 g, 54%) as a viscous oil.

^{1}H NMR (400 MHz, $CDCl_3$): δ 7.16 (s, 1 H, H-8), 6.56 (t, 1 H, *J* = 6.4 Hz, H-1'), 4.79 (AB d, *J* = 13.6 Hz, 7-CH_2a), 4.75 (AB d, *J* = 13.6 Hz, 7-CH_2b), 4.70 (s, 1 H, 2-NH), 4.50 (m, 1 H, H-3'), 3.96 (m, 1 H, H-4'), 3.76 (m, 2 H, H-5'a and H-5'b), 2.23 (m, 2 H, H-2'a and H-2'b), 0.98 (s, 9 H, $(CH_3)_3CSi$), 0.94 (s, 9 H, $(CH_3)_3CSi$), 0.92 (s, 9 H, $(CH_3)_3CSi$), 0.30 (s, 3 H, $(CH_3)_2Si$), 0.29 (s, 3 H, $(CH_3)_2Si$), 0.11 (s, 6 H, $(CH_3)_2Si$) , 0.10 (s, 6 H, $(CH_3)_2Si$).

To a solution of compound **21** (150 mg, 0.23 mmol) in CH_2Cl_2 (3.0 mL), *n*-Bu_4NBr (37 mg, 0.12 mmol), 2-nitrobenzyl bromide (148 mg, 0.68 mmol) and NaOH solution (1 M, 3.0 mL) were added. The reaction mixture was stirred vigorously at room temperature for two days in the dark. The organic phase was separated, dried over Na_2SO_4, concentrated *in vacuo,* and purified by silica gel chromatography to yield 9-[β-D-3',5'-*O*-bis-(*tert*-butyldimethylsilyl)-2'-deoxyribofuranosyl]-2-(*tert*-butyldimethylsilyl)amino-6-chloro-7-(2-nitrobenzyloxy)methyl-7-deazapurine **22** (87 mg, 48%) as a viscous oil.

^{1}H NMR (400 MHz, $CDCl_3$): δ 8.06 (dd, 1H, *J* = 8.0 and 1.2 Hz, Ph-H), 7.87 (d, 1 H, *J* = 7.2 Hz, Ph-H), 7.61 (dt, 1 H, *J* = 7.6 and 1.2 Hz, Ph-H), 7.43 (m, 1 H, Ph-H), 7.20 (s, 1 H, H-8), 6.56 (dd, 1 H, *J* = 7.6 and 6.0 Hz, H-1'), 4.99 (s, 2 H, $PhCH_2$), 4.83 (AB d, 1 H, *J* = 11.4 Hz, 7-CH_2a), 4.75 (AB d, 1 H, *J* = 11.4 Hz, 7-CH_2b), 4.67 (s, 1 H, 2-NH), 4.50 (m, 1 H, H-3'), 3.96 (m, 1 H, H-4'), 3.77 (m, 2 H, H-5'a and H-5'b), 2.25 (m, 2 H, H-2'a and H-2'b), 0.98 (s, 9 H, $(CH_3)_3CSi$), 0.92 (s, 18 H, $(CH_3)_3CSi$), 0.30 (s, 3 H, $(CH_3)_2Si$), 0.29 (s, 3 H, $(CH_3)_2Si$), 0.09 (m, 12 H, $(CH_3)_2Si$).

A solution of *n*-Bu_4NF (123 mg, 0.39 mmol) in THF (2.0 mL) was added dropwise to a solution of compound **22** (105 mg, 0.13 mmol) in THF (3.0 mL) at 0°C. The reaction mixture was stirred at 0°C for one hour and then at room temperature for two hours. The mixture was concentrated *in vacuo* and purified by silica gel chromatography to yield 2-amino-6-chloro-9-[β-D-2'-deoxy-ribofuranosyl]-7-(2-nitrobenzyloxy)methyl-7-deazapurine **23** (57 mg, 95%) as a yellow foam.

^{1}H NMR (400 MHz, DMSO-d6): δ, 8.02 (m, 1 H, Ph-H), 7.74 (m, 2 H, Ph-H), 7.55 (m, 1 H, Ph-H), 7.41 (s, 1 H, H-8), 6.73 (s, 2 H, D_2O exchangeable, NH_2), 6.41 (dd, 1 H, *J* = 8.4 and 6.0 Hz, H-1'), 5.26 (d, 1 H, D_2O exchangeable, 3'-OH), 4.91 (t, 1 H, D_2O exchangeable, 5'-OH), 4.88 (s, 2 H, Ph-CH_2), 4.66 (dd, 2 H, *J* = 11.6 Hz, 7-CH_2), 4.31 (m, 1 H, H-3'), 3.78 (m, 1 H, H-4'), 3.50 (m, 2 H, H-5'), 2.38 (m, 1 H, H-2'a), 2.15 (m, 1 H, H-2'b).

A mixture of **23** (38 mg, 0.085 mmol) and 1,4-diazabicyclo[2.2.2]octane (11 mg, 0.1 mmol) in water (4.0 mL) was heated to reflux for four hours under a nitrogen atmosphere. Water was removed *in vacuo*, and the residue was evaporated from MeOH (3.0 mL) three times, and purified by silica gel chromatography to yield 7-(2-nitrobenzyloxy)methyl-7-deaza-2'-deoxyguanosine **24** (11 mg, 30%).

^{1}H NMR (400 MHz, DMSO-d6): δ 10.4 (s, 1 H, D_2O exchangeable, N-H), 8.03 (dd, 1 H, *J* = 8.4 and 0.8 Hz, Ph-H), 7.83 (d, 1 H, *J* = 7.6 Hz, Ph-H), 7.73 (m, 1 H, Ph-H), 7.55 (m, 1 H, Ph-H), 6.92 (s, 1 H, H-8), 6.28 (m, 1 H, H-1'), 6.26 (bs, 2 H, D_2O exchangeable, NH_2), 5.21 (d, 1 H, D_2O exchangeable, 3'-OH), 4.89 (t, 1 H, D_2O exchangeable, 5'-OH), 4.88 (s, 2 H, Ph-CH_2), 4.60 (dd, 2 H, 7-CH_2), 4.28 (m, 1 H, H-3'), 3.74 (m, 1 H, H-4'), 3.48 (m, 2 H, H-5'), 2.32 (m, 1 H, H-2'a), 2.08 (m, 1 H, H-2'b).

Compound **24** (11 mg, 0.025 mmol) was phosphorylated with $POCl_3$ (15 µL, 0.05 mmol) and proton sponge (11 mg, 0.05 mmol) in trimethylphosphate (0.3 mL) at 0°C for two hours under a nitrogen atmosphere. A solution of bis-tri-*n*-butylammonium pyrophosphate (118 mg, 0.25 mmol) and tri-*n*-butylamine (50 µL) in anhydrous DMF (0.5 mL) was added. After 30 min of stirring, triethylammonium bicarbonate buffer (1 M, pH 7.5; 5.0 mL) was added. The reaction was stirred at room temperature for one hour and then concentrated *in vacuo*. The residue was dissolved in water (10 mL), filtered, and purified by anion exchange chromatography. The fractions containing triphosphate were combined and lyophilized to give 7-(2-nitrobenzyloxy)methyl-7-deaza-2'-deoxyguanosine-5'-triphosphate **dG.I**, which was further purified using RP-HPLC.

HRMS (ESI): For the molecular ion $C_{19}H_{23}N_5O_{16}P_3$ $[M-H]^-$, the calculated mass was 670.0353, and the observed mass was 670.0344.

7-[1-(2-Nitrophenyl)-2,2-dimethyl-propyloxy]methyl-7-deaza-2'-deoxyguanosine-5'-triphosphate

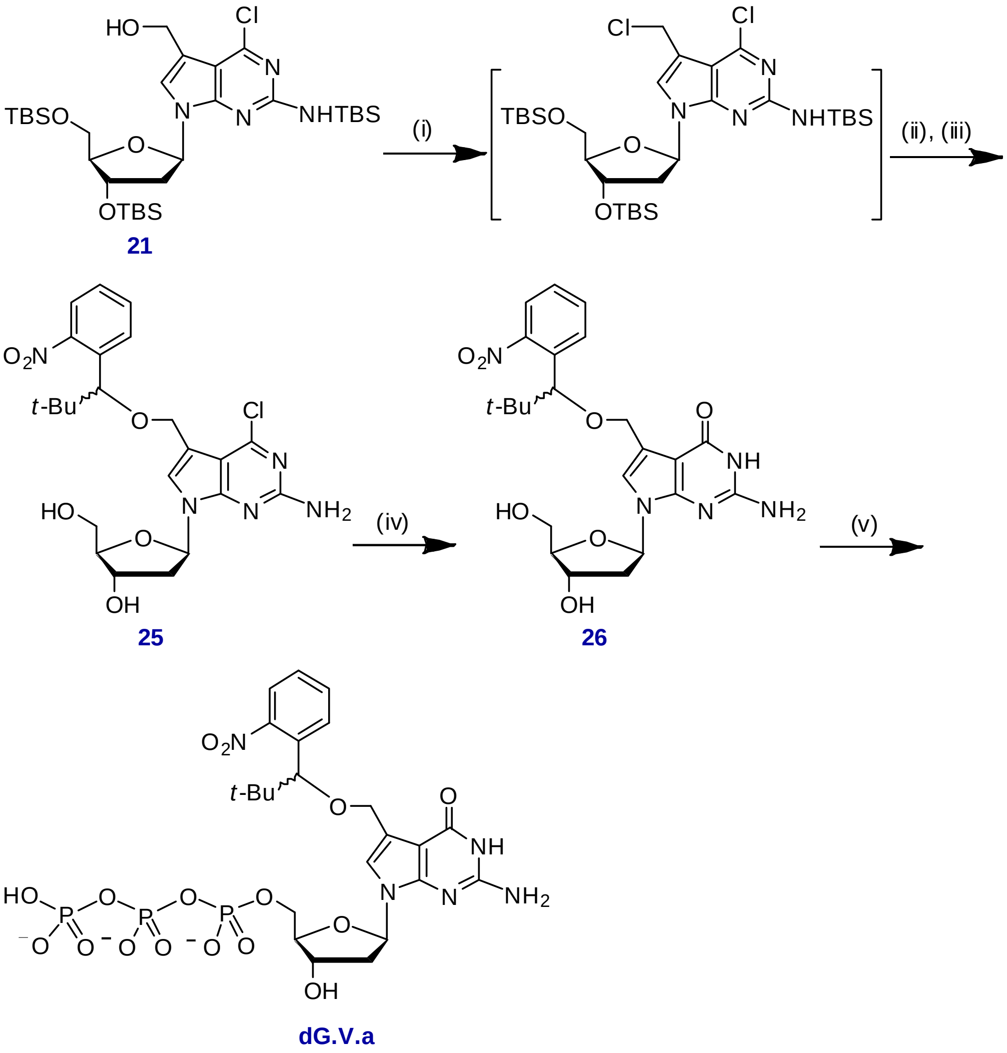

Scheme S13. Synthesis of 7-[1-(2-nitrophenyl)-2,2-dimethyl-propyloxy]methyl-7-deaza-2'-deoxy-guanosine-5'-triphosphate. *Reagents and conditions*: (*i*) MsCl, DMAP, CH_2Cl_2, 0°C; (*ii*) racemic (*R*/*S*)-1-(2-nitrophenyl)-2,2-dimethyl-1-propanol, 115°C; (*iii*) *n*-Bu_4NF, THF, room temperature, 26% for three steps; (*iv*) syn-pyridine-2-aldoxime, 1,1,3,3-tetramethyl guanidine, 1,4-dioxane/DMF, 70°C, 70%; (*v*) $POCl_3$, proton sponge, $(MeO)_3PO$, 0°C; $(n\text{-}Bu_3NH)_2H_2P_2O_7$, *n*-$Bu_3N$, DMF; 1 M $HNEt_3HCO_3$.

DMAP (148 mg, 1.2 mmol) and MsCl (71 µL, 0.9 mmol) were added to a solution of compound **21** (200 mg, 0.30 mmol) in anhydrous CH_2Cl_2 (5.0 mL) at 0°C under a nitrogen atmosphere. The reaction was stirred at 0°C for 10 min and diluted with CH_2Cl_2 (15 mL). The solution was applied on a short silica gel plug (2 × 3 cm) and was eluted quickly with hexane/ethyl acetate/triethylamine solvent system (volume ratio: 80/20/0.5). The eluent was concentrated *in vacuo*, and the residue was mixed with racemic (*R*/*S*)-1-(2-nitrophenyl)-2,2-dimethyl-

1-propanol (500 mg, 2.4 mmol). The mixture was heated at 115°C for 45 min under a nitrogen atmosphere, cooled to room temperature and then dissolved in THF (10 mL) followed by addition of *n*-Bu_4NF (283 mg, 0.90 mmol). The mixture was stirred at room temperature for four hours and then concentrated *in vacuo*. The residue was dissolved in CH_2Cl_2 (25 mL) and washed with brine (25 mL), and the aqueous phase was extracted with CH_2Cl_2 (25 mL) two times. The combined organic phase was dried over Na_2SO_4, concentrated *in vacuo*, and the residue was purified by silica gel chromatography to yield 2-amino-6-chloro-9-[β-D-2'-deoxyribofuranosyl]-7-[1-(2-nitrophenyl)-2,2-dimethyl-propyloxy] methyl-7-deazapurine **25** (40 mg, 26% for three steps) as a 1:1 mixture of two diastereomers.

To a solution of compound **25** (40 mg, 0.08 mmol) in 1,4-dioxane (1.0 mL) and DMF (2.0 mL), syn-pyrimidine-2-aldoxime (180 mg, 1.5 mmol) and 1,1,3,3-tetramethyl guanidine (211 µL, 1.68 mmol) were added. The mixture was heated at 70°C overnight under a nitrogen atmosphere. The reaction mixture was diluted with CH_2Cl_2 (20 mL) and washed sequentially with acetic acid solution (0.1 M, 30 mL), saturated $NaHCO_3$ solution (30 mL), and brine (30 mL). The organic phase was dried over Na_2SO_4, concentrated *in vacuo*, and the residue was purified by silica gel chromatography to yield 7-[1-(2-nitrophenyl)-2,2-dimethyl-propyloxy]methyl-7-deaza-2'-deoxyguanosine **26** (27 mg, 70%) as a 1:1 mixture of two diastereomers.

^{1}H NMR (400 MHz, MeOH-d_4) for diastereomers: δ 7.79 (m, 1 H, Ph-H), 7.73 (m, 1 H, Ph-H), 7.56 (m, 1 H, Ph-H), 7.39 (m, 1 H, Ph-H), 6.87 and 6.86 (2 s, 1 H, H-8), 6.30 (m, 1 H, H-1'), 4.99 and 4.97 (2 s, 1 H, Ph-CH), 4.63-4.36 (m, 3 H, 7-CH_2 and H-3'), 3.91 (m, 1 H, H-4'), 3.69 (m, 2 H, H-5'), 2.48 (m, 1 H, H-2'a), 2.20 (m, 1 H, H-2'b), 0.79 and 0.77 (2 s, 9 H, $(CH_3)_3$).

Compound **26** (25 mg, 0.05 mmol) was phosphorylated with $POCl_3$ (20 µL, 0.21 mmol) and proton sponge (21 mg, 0.1 mmol) in trimethylphosphate (0.35 mL) at 0°C for 3.5 hours under a nitrogen atmosphere. A solution of bis-tri-*n*-butylammonium pyrophosphate (237 mg, 0.50 mmol) and tri-*n*-butylamine (100 µL) in anhydrous DMF (1.0 mL) was added. After 10 min of stirring, triethylammonium bicarbonate buffer (0.1 M, pH 7.5; 10 mL) was added. The reaction was stirred at room temperature for one hour and then concentrated *in vacuo*. The residue was dissolved in 20% aqueous acetonitrile (20 mL), filtered, and purified by anion exchange chromatography. The fractions containing triphosphate were combined and lyophilized to give 7-[1-(2-nitrophenyl)-2,2-dimethyl-propyloxy]methyl-7-deaza-2'-deoxy-

guanosine-5'-triphosphate **dG.V.a** as a 1:1 mixture of two diastereomers, which were separated using RP-HPLC to yield the single diastereomers **dG.V.a ds1** and **dG.V.a ds2**.

HRMS (ESI): For the molecular ion $C_{23}H_{31}N_5O_{16}P_3$ $[M-H]^-$, the calculated mass was 726.0979, and the observed mass was 726.0992.

7-[(S)-1-(2-Nitrophenyl)-2,2-dimethyl-propyloxy]methyl-7-deaza-2'-deoxyguanosine-5'-triphosphate

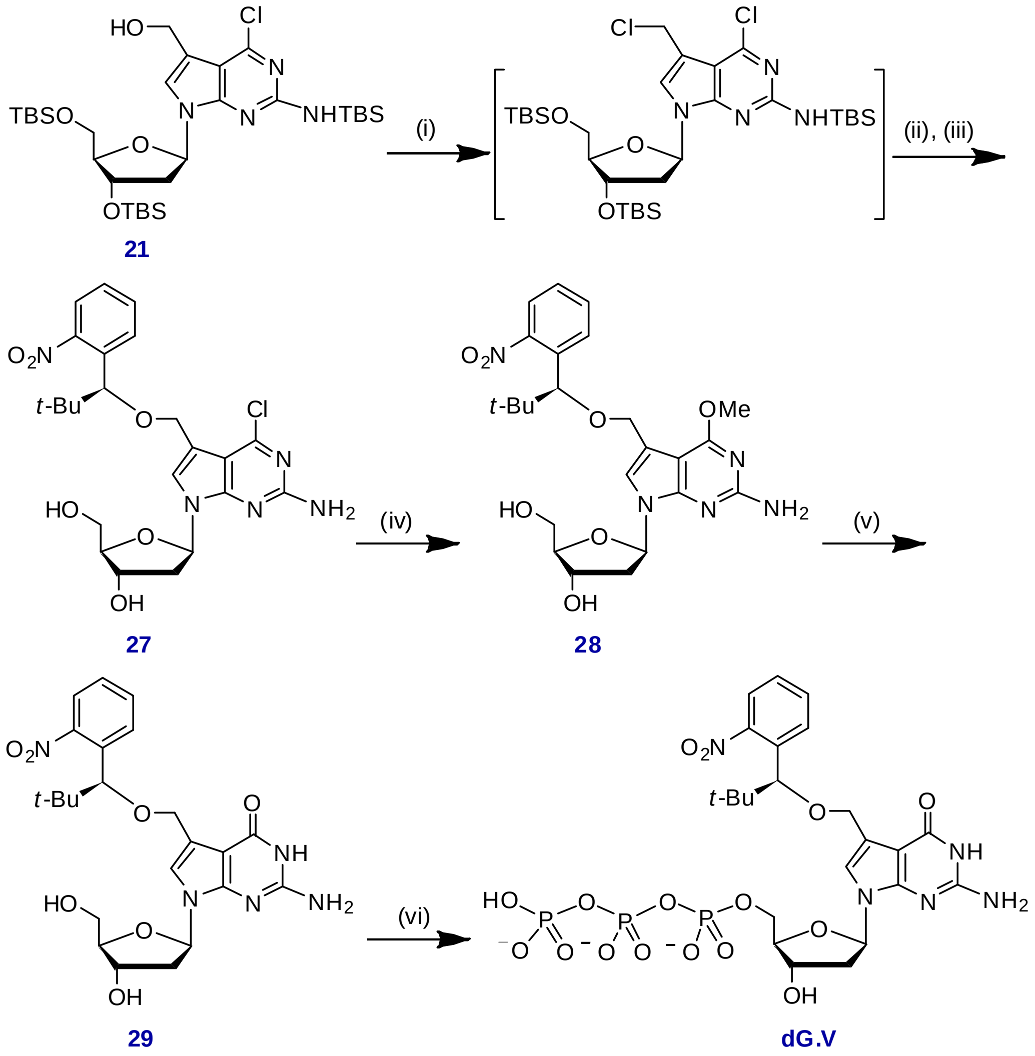

Scheme S14. Synthesis of 7-[(*S*)-1-(2-nitrophenyl)-2,2-dimethyl-propyloxy]methyl-7-deaza-2'-deoxy-guanosine-5'-triphosphate. *Reagents and conditions*: (*i*) MsCl, DMAP, CH_2Cl_2, 0°C; (*ii*) (*S*)-1-(2-nitrophenyl)-2,2-dimethyl-1-propanol, 115°C; (*iii*) *n*-Bu_4NF, THF, room temperature, 35% for three steps; (*iv*) NaOMe, MeOH, reflux, 74%; (*v*) 1,4-dioxane, 2 M NaOH, reflux, 33%; (*vi*) $POCl_3$, proton sponge, $(MeO)_3PO$, 0°C; $(n\text{-}Bu_3NH)_2H_2P_2O_7$, *n*-$Bu_3N$, DMF; 1 M $HNEt_3HCO_3$.

DMAP (224 mg, 1.8 mmol) and MsCl (107 µL, 1.4 mmol) were added to a solution of compound **21** (300 mg, 0.46 mmol) in anhydrous CH_2Cl_2 (10 mL) at 0°C under a nitrogen atmosphere. The reaction was stirred at 0°C for 10 min and diluted with CH_2Cl_2 (20 mL). The solution was applied on a short silica gel plug (2 × 3 cm) and was eluted quickly with hexane/ethyl acetate/triethylamine solvent system (volume ratio 80/20/0.5). The eluent was concentrated *in vacuo*, and residue was mixed with (*S*)-1-(2-nitrophenyl)-2,2-dimethyl-1-propanol (520 mg, 2.5 mmol). The mixture was heated at 115°C for 45 min under a nitrogen atmosphere, cooled to room temperature and dissolved in THF (20 mL) followed by addition of *n*-Bu_4NF (491 mg, 1.6 mmol). The mixture was stirred at room temperature for four hours and then concentrated *in vacuo*. The residue was dissolved in CH_2Cl_2 (20 mL) and washed with brine (30 mL), and the aqueous phase was extracted with CH_2Cl_2 (20 mL) two times. The combined organic phase was dried over Na_2SO_4, concentrated *in vacuo*, and the residue was purified by silica gel chromatography to yield 2-amino-6-chloro-9-[β-D-2'-deoxyribofuranosyl]-7-[(*S*)-1-(2-nitrophenyl)-2,2-dimethyl-propyloxy]methyl-7-deazapurine **27** (81 mg, 35% for three steps).

^{1}H NMR (400 MHz, MeOH-d_4): δ 7.79 (m, 2 H, Ph-H), 7.60 (dt, 1 H, *J* = 1.2 and 8.0 Hz, Ph-H), 7.46 (dt, 1 H, *J* = 1.2 and 8.0 Hz, Ph-H), 7.27 (s, 1 H, H-8), 6.47 (dd, 1 H, *J* = 6.4 and 8.0 Hz, H-1'), 4.98 (s, 1 H, Ph-CH), 4.71 (d, 1 H, *J* = 12.4 Hz, 7-CH_2 a), 4.50 (m, 1 H, H-3'), 4.47 (d, 1 H, *J* = 12.4 Hz, 7-CH_2 b), 3.96 (m, 1 H, H-4'), 3.73 (m, 2 H, H-5'), 2.59 (m, 1 H, H-2'a), 2.30 (m, 1 H, H-2'b), 0.80 (s, 9 H, $(CH_3)_3$).

Compound **27** (104 mg, 0.21 mmol) was dissolved in a solution of sodium methoxide in MeOH (0.5 M, 10 mL), and the mixture was heated to reflux for one hour under a nitrogen atmosphere. The reaction mixture was cooled to room temperature, neutralized with acetic acid, and then concentrated *in vacuo*. The residue was purified by silica gel chromatography to yield 2-amino-6-methoxy-9-[β-D-2'-deoxyribofuranosyl]-7-[(*S*)-1-(2-nitrophenyl)-2,2-dimethyl-propyloxy]methyl-7-deazapurine **28** (75 mg, 74%).

^{1}H NMR (400 MHz, $CDCl_3$): δ 7.74 (m, 2 H, Ph-H), 7.52 (t, 1 H, *J* = 8.0 Hz, Ph-H), 7.36 (t, 1 H, *J* = 8.0 Hz, Ph-H), 6.71 (s, 1 H, H-8), 6.47 (dd, 1 H, *J* = 5.6 and 9.6 Hz, H-1'), 5.04 (s, 1 H, Ph-CH), 4.71 (m, 1 H, H-3'), 4.47 (dd, 2 H, *J* = 12 Hz, 7-CH_2), 4.15 (m, 1 H, H-4'), 3.94 (s, 3 H, OCH_3), 3.76 (m, 2 H, H-5'), 3.01 (m, 1 H, H-2'a), 2.19 (m, 1 H, H-2'b), 0.82 (s, 9 H, $(CH_3)_3$).

Compound **28** (70 mg, 0.14 mmol) was dissolved in 1,4-dioxane (6.0 mL) followed by addition of an aqueous solution of sodium hydroxide (2 M, 12 mL). The mixture was heated to reflux for

four days under a nitrogen atmosphere, cooled to room temperature, neutralized with dilute hydrochloric acid (1 M), and concentrated *in vacuo*. The residue was evaporated from MeOH (5.0 mL) three times and then purified by silica gel chromatography to yield 7-[(*S*)-1-(2-nitrophenyl)-2,2-dimethyl-propyloxy]methyl-7-deaza-2'-deoxyguanosine **29** (22 mg, 33%). Starting material **28** (42 mg, 60%) was also recovered from the reaction.

^{1}H NMR (400 MHz, $CDCl_3$): δ 11.02 (br s, 1 H, NH), 7.69 (m, 2 H, Ph-H), 7.52 (t, 1 H, *J* = 7.2 Hz, Ph-H), 7.33 (t, 1 H, *J* = 7.2 Hz, Ph-H), 6.66 (s, 1 H, H-8), 6.13 (t, 1 H, *J* = 6.8 Hz, H-1'), 6.03 (br s, 2 H, 6-NH_2), 4.92 (s, 1 H, Ph-CH), 4.77 (m, 1 H, H-3'), 4.57 (d, 1 H, *J* = 12.8 Hz, 7-CH_2 a), 4.12 (m, 1 H, H-4'), 3.05 (d, 1 H, *J* = 12.8 Hz, 7-CH_2 b), 3.75 (m, 2 H, H-5'), 2.87 (m, 1 H, H-2'a), 2.29 (m, 1 H, H-2'b), 0.76 (s, 9 H, $(CH_3)_3$).

Compound **29** (16 mg, 0.033 mmol) was phosphorylated with $POCl_3$ (17 µL, 0.18 mmol) and proton sponge (14 mg, 0.066 mmol) in trimethylphosphate (0.35 mL) at 0°C for four hours under a nitrogen atmosphere. A solution of bis-tri-*n*-butylammonium pyrophosphate (237 mg, 0.50 mmol) and tri-*n*-butylamine (100 µL) in anhydrous DMF (1.0 mL) was added. After 10 min of stirring, triethylammonium bicarbonate buffer (0.1 M, pH 7.5; 10 mL) was added. The reaction was stirred at room temperature for one hour and then concentrated *in vacuo*. The residue was dissolved in 20% aqueous acetonitrile (20 mL), filtered, and purified by anion exchange chromatography. The fractions containing triphosphate were combined and lyophilized to give 7-[(*S*)-1-(2-nitrophenyl)-2,2-dimethyl-propyloxy]methyl-7-deaza-2'-deoxyguanosine-5'-triphosphate **dG.V**, which was further purified using RP-HPLC conditions. The retention time of **dG.V** was identical to that of **dG.V.a ds2** by RP-HPLC analysis using the same condition (data not shown).

HRMS (ESI): For the molecular ion $C_{23}H_{31}N_5O_{16}P_3$ [M-H]$^-$, the calculated mass was 726.0979, and the observed mass was 726.0986.

7-[1-(4-Methoxy-2-nitrophenyl)-2,2-dimethyl-propyloxy]methyl-7-deaza-2’-deoxyguanosine-5’-triphosphate

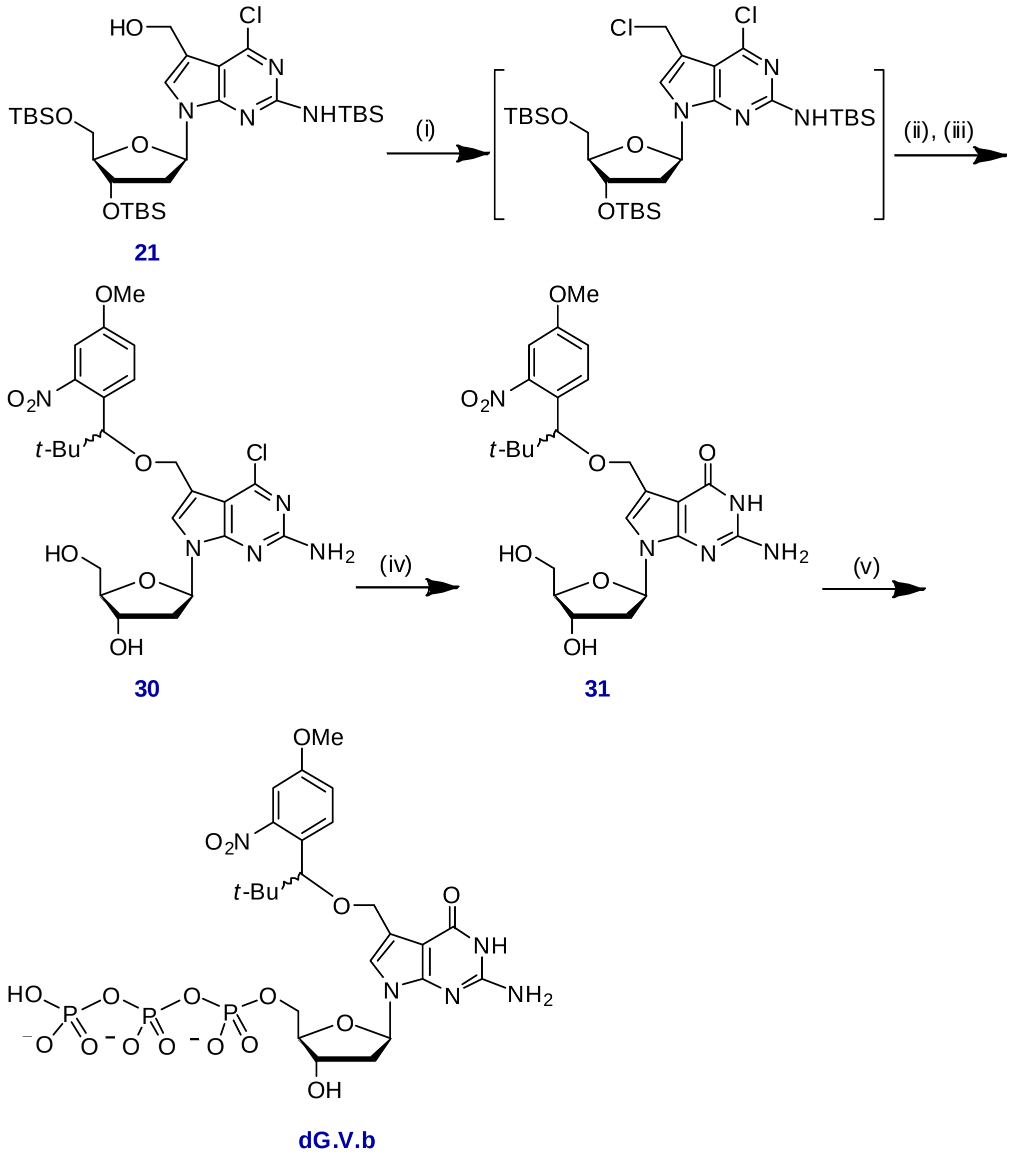

Scheme S15. Synthesis of 7-[1-(4-methoxy-2-nitrophenyl)-2,2-dimethyl-propyloxy]methyl-7-deaza-2’-deoxyguanosine-5’-triphosphate. *Reagents and conditions*: (*i*) MsCl, DMAP, CH_2Cl_2, 0°C; (*ii*) racemic (*R*/*S*)-1-(4-methoxy-2-nitrophenyl)-2,2-dimethyl-1-propanol, 115°C; (*iii*) *n*-Bu_4NF, THF, room temperature, 21% for three steps; (*iv*) syn-pyridine-2-aldoxime, 1,1,3,3-tetramethyl guanidine, 1,4-dioxane/DMF, 70°C, 59%; (*v*) $POCl_3$, proton sponge, $(MeO)_3PO$, 0°C; $(n\text{-}Bu_3NH)_2H_2P_2O_7$, *n*-$Bu_3N$, DMF; 1 M $HNEt_3HCO_3$.

DMAP (346 mg, 2.8 mmol) and MsCl (165 µL, 2.1 mmol) were added to a solution of compound **21** (470 mg, 0.72 mmol) in anhydrous CH_2Cl_2 (5.0 mL) at 0°C under a nitrogen atmosphere. The reaction was stirred at 0°C for 10 min and diluted with CH_2Cl_2 (20 mL). The solution was applied

on a short silica gel plug (2 × 3 cm) and was eluted quickly with hexane/ethyl acetate/triethylamine solvent system (volume ratio 80/20/0.5). The eluent was concentrated *in vacuo*, and the residue was mixed with racemic (*R*/*S*)-1-(4-methoxy-2-nitrophenyl)-2,2-dimethyl-1-propanol (1.6 g, 6.69 mmol). The mixture was heated to 115°C for 45 min under a nitrogen atmosphere, cooled to room temperature, and dissolved in THF (20 mL) followed by addition of *n*-Bu_4NF (788 mg, 2.5 mmol). The mixture was stirred at room temperature for four hours and then concentrated *in vacuo*. The residue was dissolved in CH_2Cl_2 (20 mL) and washed with brine (30 mL), and the aqueous phase was extracted with CH_2Cl_2 (20 mL) two times. The combined organic phase was dried over Na_2SO_4, concentrated *in vacuo*, and the residue was purified by silica gel chromatography to yield 2-amino-6-chloro-9-[β-D-2'-deoxyribofuranosyl]-7-[(*S*)-1-(4-methoxy-2-nitrophenyl)-2,2-dimethyl-propyloxy]-methyl-7-deazapurine **30** (80 mg, 21% for three steps) as a 1:1 mixture of two diastereomers.

To a solution of compound **30** (80 mg, 0.15 mmol) in 1,4-dioxane (1.0 mL) and DMF (2.0 mL), syn-pyrimidine-2-aldoxime (366 mg, 3.0 mmol) and 1,1,3,3-tetramethyl guanidine (414 µL, 3.3 mmol) were added, and the mixture was heated at 70°C overnight under a nitrogen atmosphere. The reaction mixture was diluted with CH_2Cl_2 (20 mL) and washed sequentially with acetic acid (0.1 M, 30 mL), saturated $NaHCO_3$ solution (30 mL), and brine (30 mL). The organic phase was dried over Na_2SO_4, concentrated *in vacuo*, and the residue was purified by silica gel chromatography to yield 7-[(*S*)-1-(4-methoxy-2-nitrophenyl)-2,2-dimethyl-propyloxy]methyl-7-deaza-2'-deoxy-guanosine **31** (45 mg, 59%) as a 1:1 mixture of two diastereomers.

^1H NMR (400 MHz, DMSO-d_6) for diastereomers: δ 10.31 (br s, 1 H, D_2O exchangable, NH), 7.63 and 7.62 (2 d, 1 H, *J* = 2,8 Hz, Ph-H), 7.41 and 7.40 (2 d, 1 H, *J* = 2,8 Hz, Ph-H), 7.27 (m, 1 H, Ph-H), 6.98 and 6.96 (2 s, 1 H, H-8), 6.28 (m, 1 H, H-1'), 6.22 (br s, 2 H, D_2O exchangable, NH_2), 5.22 (d, 1 H, D_2O exchangable, 3'-OH), 4.88 (t, 1 H, D_2O exchangable, 5'-OH), 4.73 and 4.71 (2 s, 1 H, Ph-CH), 4.47-4.24 (m, 3 H, 7-CH_2 and H-3'), 3.85 and 3.83 (2 s, 3 H, OCH_3), 3.74 (m, 1 H, H-4'), 3.48 (m, 2 H, H-5'), 2.28 (m, 1 H, H-2'a), 2.06 (m, 1 H, H-2'b), 0.80 and 0.78 (2 s, 9 H, $(CH_3)_3$).

Compound **31** (25 mg, 0.048 mmol) was phosphorylated with $POCl_3$ (15 µL, 0.18 mmol) and proton sponge (21 mg, 0.10 mmol) in trimethylphosphate (0.35 mL) at 0°C for 3.5 hours under a

nitrogen atmosphere. A solution of bis-tri-*n*-butylammonium pyrophosphate (237 mg, 0.50 mmol) and tri-*n*-butylamine (100 µL) in anhydrous DMF (1.0 mL) was added. After 10 min of stirring, triethylammonium bicarbonate buffer (0.1 M, pH 7.5; 10 mL) was added. The reaction was stirred at room temperature for one hour and then concentrated *in vacuo*. The residue was dissolved in 20% aqueous acetonitrile (20 mL), filtered, and purified by anion exchange chromatography. The fractions containing triphosphate were combined and lyophilized to give 7-[1-(4-methoxy-2-nitrophenyl)-2,2-dimethyl-propyloxy]methyl-7-deaza-2'-deoxyguanosine-5'-triphosphate **dG.V.b** as a 1:1 mixture of two diastereomers, which were separated using RP-HPLC to yield the single diastereomers **dG.V.b ds1** and **dG.V.b ds2**.

HRMS (ESI): For the molecular ion $C_{24}H_{33}N_5O_{17}P_3$ $[M-H]^-$, the calculated mass was 756.1084, and the observed mass was 756.1101.

7-[1-(5-Methoxy-2-nitrophenyl)-2,2-dimethyl-propyloxy]methyl-7-deaza-2'-deoxyguanosine-5'-triphosphate

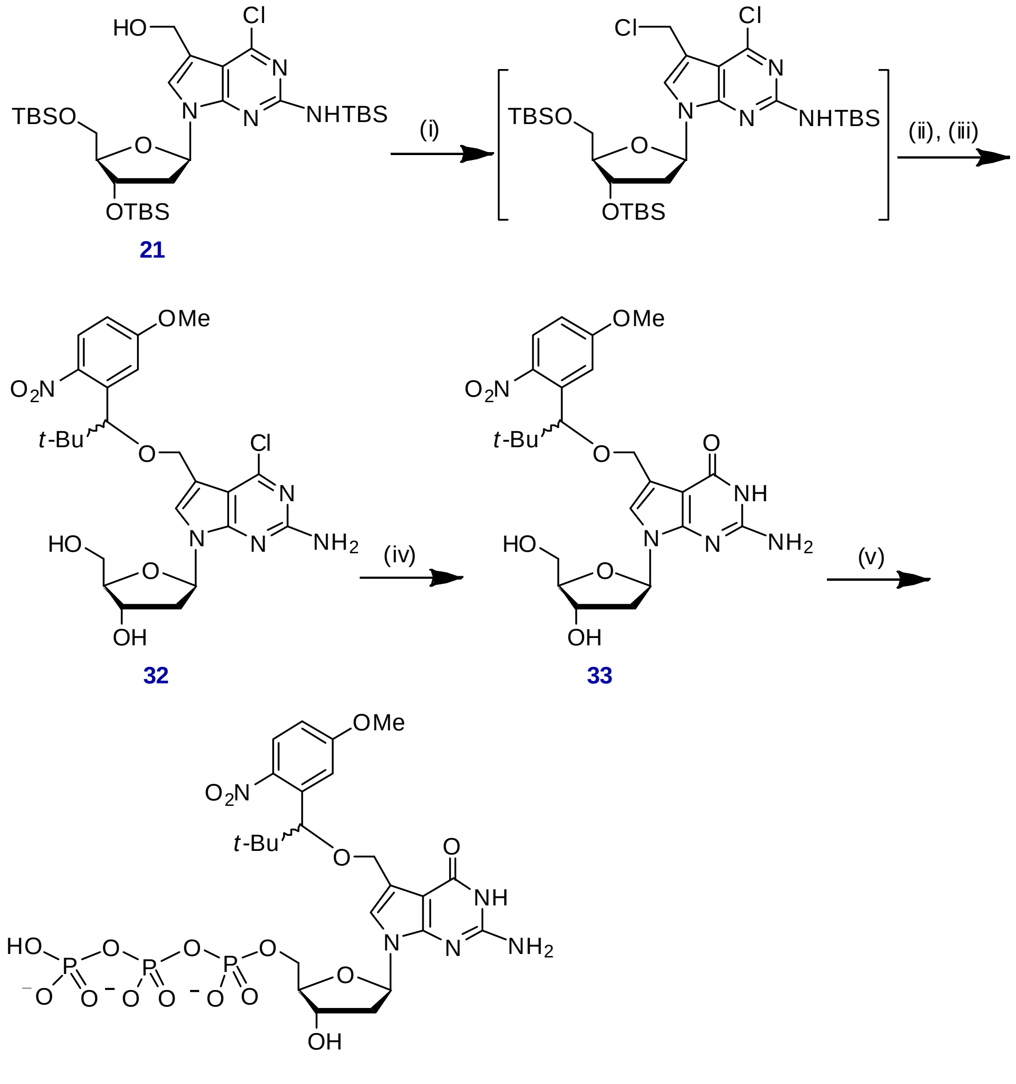

Scheme S16. Synthesis of 7-[1-(5-methoxy-2-nitrophenyl)-2,2-dimethyl-propyloxy]methyl-7-deaza-2'-deoxyguanosine-5'-triphosphate. *Reagents and conditions*: (*i*) MsCl, DMAP, CH_2Cl_2, 0°C; (*ii*) racemic (*R*/*S*)-1-(5-methoxy-2-nitrophenyl)-2,2-dimethyl-1-propanol, 115°C; (*iii*) *n*-Bu_4NF, THF, room temperature, 24% for three steps; (*iv*) syn-pyridine-2-aldoxime, 1,1,3,3-tetramethyl guanidine, 1,4-dioxane/DMF, 70°C, 57%; (*v*) $POCl_3$, proton sponge, $(MeO)_3PO$, 0°C; $(n\text{-}Bu_3NH)_2H_2P_2O_7$, *n*-$Bu_3N$, DMF; 1 M $HNEt_3HCO_3$.

DMAP (302 mg, 2.5 mmol) and MsCl (145 µL, 1.9 mmol) were added to a solution of compound **21** (410 mg, 0.62 mmol) in anhydrous CH_2Cl_2 (5.0 mL) at 0°C under a nitrogen atmosphere. The reaction was stirred at 0°C for 10 min and diluted with CH_2Cl_2 (20 mL). The solution was applied on a short silica gel plug (2 × 3 cm) and was eluted quickly with a hexane/ethyl

acetate/triethylamine solvent system (volume ratio: 80/20/0.5). The eluent was concentrated *in vacuo*, and residue was mixed with racemic (*R*/*S*)-1-(5-methoxy-2-nitrophenyl)-2,2-dimethyl-1-propanol (800 mg, 2.2 mmol). The mixture was heated at 115°C for 45 min under a nitrogen atmosphere, cooled to room temperature, and dissolved in THF (10 mL) followed by addition of *n*-Bu_4NF (683 mg, 3.3 mmol). The mixture was stirred at room temperature for four hours and then concentrated *in vacuo*. The residue was dissolved in CH_2Cl_2 (20 mL) and washed with brine (30 mL), and the aqueous phase was extracted with CH_2Cl_2 (20 mL) two times. The combined organic phase was dried over Na_2SO_4, concentrated *in vacuo*, and the residue was purified by silica gel chromatography to yield 2-amino-6-chloro-9-[β-D-2'-deoxyribofuranosyl]-7-[1-(5-methoxy-2-nitrophenyl)-2,2-dimethyl-propyloxy]methyl-7-deazapurine **32** (80 mg, 24% for three steps) as a 1:1 mixture of two diastereomers.

^{1}H NMR (400 MHz, $CDCl_3$) for diastereomers: δ 7.86 and 7.83 (2 d, 1 H, *J* = 8.8 Hz, Ph-H), 7.19 and 7.17 (2 d, 1 H, *J* = 2.8 Hz, Ph-H), 6.91 and 6.90 (2 s, 1 H, H-8), 6.80 and 6.75 (2 dd, 1 H, *J* = .8 and 8.8 Hz, Ph-H), 6.17 (m, 1 H, H-1'), 5.23 and 5.21 (2 s, 1 H, Ph-CH), 5.01 and 5.00 (2 br s, 2 H, NH_2), 4.73 (m, 1 H, H-3'), 4.65-4.49 (m, 2 H, 7-CH_2), 4.14 (m, 1 H, H-4'), 3.84 (m, 5 H, H-5' and OCH_3), 2.78 (m, 1 H, H-2'a), 2.33 (m, 1 H, H-2'b), 0.82 and 0.81 (2 s, 9 H, $(CH_3)_3$).

To a solution of compound **32** (80 mg, 0.15 mmol) in 1,4-dioxane (1.0 mL) and DMF (2.0 mL), syn-pyrimidine-2-aldoxime (360 mg, 3.0 mmol) and 1,1,3,3-tetramethyl guanidine (414 µL, 3.3 mmol) were added, and the mixture was heated at 70°C overnight under a nitrogen atmosphere. The reaction mixture was diluted with CH_2Cl_2 (20 mL) and washed sequentially with acetic acid (0.1 M, 30 mL), saturated $NaHCO_3$ solution (30 mL), and brine (30 mL). The organic phase was dried over Na_2SO_4, concentrated *in vacuo*, and the residue was purified by silica gel chromatography to yield 7-[1-(5-methoxy-2-nitrophenyl)-2,2-dimethyl-propyloxy]methyl-7-deaza-2'-deoxyguanosine **33** (43 mg, 57%) as a 1:1 mixture of two diastereomers.

^{1}H NMR (400 MHz, DMSO-d_6) for diastereomers: δ 10.34 (br s, 1 H, D_2O exchangable, NH), 7.92 and 7.89 (2 d, 1 H, *J* = 8,8 Hz, Ph-H), 7.15 (m, 1 H, Ph-H), 6.95 (m, 1 H, Ph-H), 6.82 and 6.81 (2 s, 1 H, H-8), 6.22 (m, 3 H, 2 H D_2O exchangable, H-1' and NH_2), 5.19 (d, 1 H, D_2O exchangable, 3'-OH), 5.12 and 5.10 (2 s, 1 H, Ph-CH), 4.84 (t, 1 H, D_2O exchangable, 5'-OH), 4.47-4.31 (m, 2 H, 7-CH_2), 4.24 (m, 1 H, H-3'), 3.85 and 3.83 (2 s, 3 H, OCH_3), 3.71 (m, 1 H, H-4'), 3.44 (m, 2 H, H-5'), 2.24 (m, 1 H, H-2'a), 2.01 (m, 1 H, H-2'b), 0.76 and 0.74 (2 s, 9 H, $(CH_3)_3$).

Compound **33** (20 mg, 0.04 mmol) was phosphorylated with $POCl_3$ (25 µL, 0.27 mmol) and proton sponge (16 mg, 0.08 mmol) in trimethylphosphate (0.30 mL) at 0°C for six hours under a nitrogen atmosphere. A solution of bis-tri-*n*-butylammonium pyrophosphate (237 mg, 0.50 mmol) and tri-*n*-butylamine (100 µL) in anhydrous DMF (1.0 mL) was added. After 10 min of stirring, triethylammonium bicarbonate buffer (0.1 M, pH 7.5; 10 mL) was added. The reaction was stirred at room temperature for one hour and then concentrated *in vacuo*. The residue was dissolved in 20% aqueous acetonitrile (20 mL), filtered, and purified by anion exchange chromatography. The fractions containing triphosphate were combined and lyophilized to give 7-[1-(5-methoxy-2-nitrophenyl)-2,2-dimethyl-propyloxy]methyl-7-deaza-2'-deoxyguanosine-5'-triphosphate **dG.V.c** as a 1:1 mixture of two diastereomers, which were separated using RP-HPLC to yield the single diastereomers **dG.V.c ds1** and **dG.V.c ds2**.

HRMS (ESI): For the molecular ion $C_{24}H_{33}N_5O_{17}P_3$ $[M-H]^-$, the calculated mass was 756.1084, and the observed mass was 756.1088.

7-[1-(4,5-Dimethoxy-2-nitrophenyl)-2,2-dimethyl-propyloxy]methyl-7-deaza-2'-deoxyguanosine-5'-triphosphate

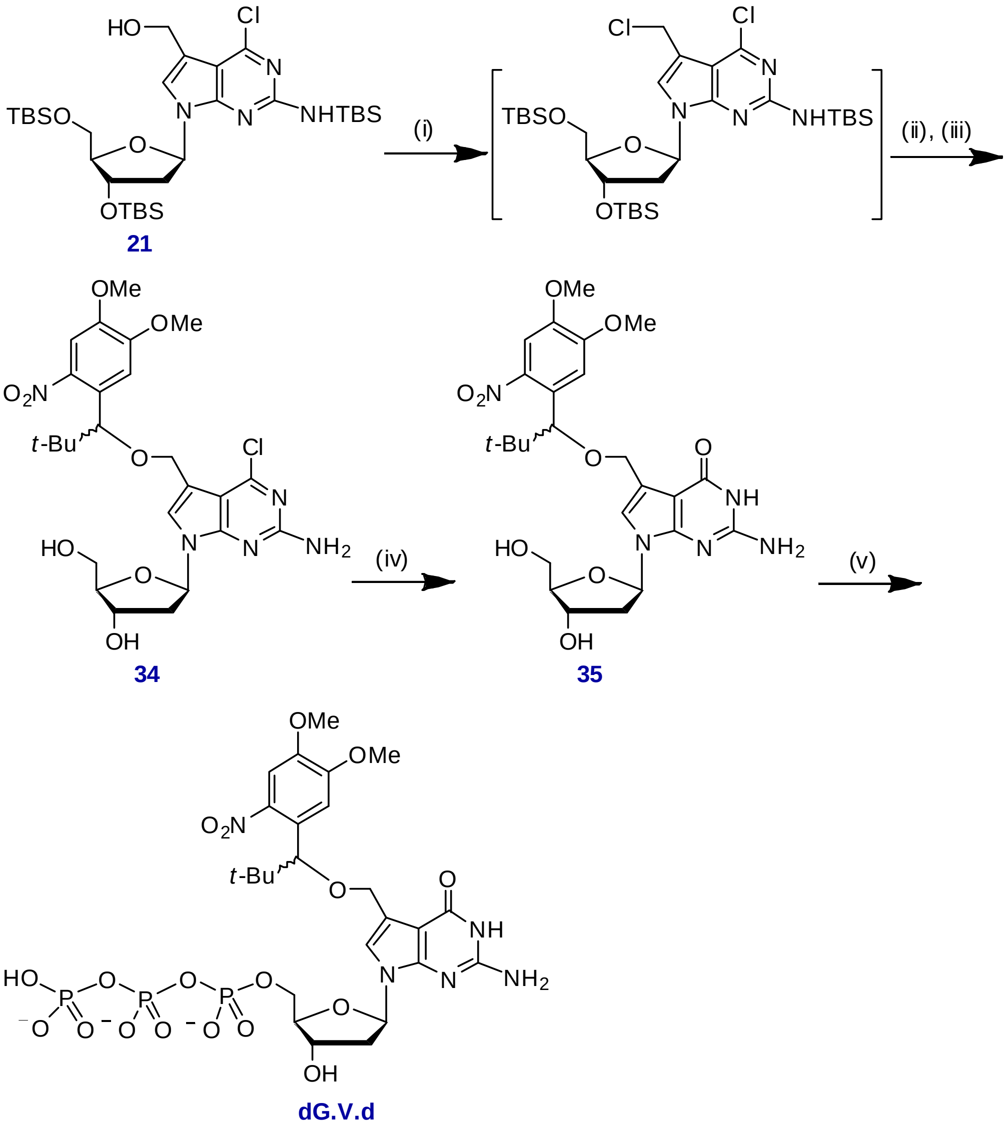

Scheme S17. Synthesis of 7-[1-(4,5-dimethoxy-2-nitrophenyl)-2,2-dimethyl-propyloxy]methyl-7-deaza-2'-deoxyguanosine-5'-triphosphate. *Reagents and conditions;* (*i*) MsCl, DMAP, CH_2Cl_2, 0°C; (*ii*) racemic (*R*/*S*)-1-(4,5-dimethoxy-2-nitrophenyl)-2,2-dimethyl-1-propanol, 115°C; (*iii*) *n*-Bu_4NF, THF, room temperature, 23% for three steps; (*iv*) syn-pyridine-2-aldoxime, 1,1,3,3-tetramethyl guanidine, dioxane/DMF, 70°C, 68%; (*v*) $POCl_3$, proton sponge, $(MeO)_3PO$, 0°C; $(n\text{-}Bu_3NH)_2H_2P_2O_7$, *n*-$Bu_3N$, DMF; 1 M $HNEt_3HCO_3$.

DMAP (273 mg, 2.2 mmol) and MsCl (130 µL, 1.7 mmol) were added to a solution of compound **21** (370 mg, 0.56 mmol) in anhydrous CH_2Cl_2 (5.0 mL) at 0°C under a nitrogen atmosphere. The reaction was stirred at 0°C for 30 min and diluted with CH_2Cl_2 (25 mL). The solution was applied on a short silica gel plug (2 × 3 cm) and was eluted quickly with a hexane/ethyl

acetate/triethylamine solvent system (volume ratio: 80/20/0.5). The eluent was concentrated *in vacuo*, and the residue was mixed with racemic (*R*/*S*)-1-(4,5-dimethoxy-2-nitrophenyl)-2,2-dimethyl-1-propanol (800 mg, 3.0 mmol). The mixture was heated at 115°C for 45 min under a nitrogen atmosphere, cooled to room temperature and dissolved in THF (10 mL) followed by addition of *n*-Bu_4NF (530 mg, 1.7 mmol). The mixture was stirred at room temperature for two hours and then concentrated *in vacuo*. The residue was dissolved in CH_2Cl_2 (40 mL) and washed with brine (50 mL), and the aqueous phase was extracted with CH_2Cl_2 (40 mL) two times. The combined organic phase was dried over Na_2SO_4 and concentrated *in vacuo*, and the residue was purified by silica gel chromatography to yield 2-amino-6-chloro-9-[β-D-2'-deoxyribofuranosyl]-7-[1-(4,5-dimethoxy-2-nitrophenyl)-2,2-dimethyl-propyloxy]methyl-7-deazapurine **34** (70 mg, 23% for three steps) as a 1:1 mixture of two diastereomers.

^{1}H NMR (400 MHz, $CDCl_3$) for diastereomers: δ 7.42 and 7.39 (2 s, 1 H, Ph-H), 7.15 and 7.13 (2 s, 1 H, Ph-H), 6.89 and 6.84 (2 s, 1 H, H-8), 6.12 (m, 1 H, H-1'), 5.22 and 5.16 (2 s, 1 H, Ph-CH), 5.10 and 5.08 (2 bs, 2 H, NH_2), 4.71-4.41 (m, 3 H, H-3'and 7-CH_2), 4.13 (m, 1 H, H-4'), 3.94 (4 s, 7 H, $OCH_3 \times 2$ and H-5'a), 3. 78 (m, 1 H, H-5'b), 2.90 (m, 1 H, H-2'a), 2.25 (m, 1 H, H-2'b), 0.82 and 0.80 (2 s, 9 H, $(CH_3)_3$).

To a solution of compound **34** (65 mg, 0.11 mmol) in 1,4-dioxane (1.0 mL) and DMF (2.0 mL), syn-pyrimidine-2-aldoxime (292 mg, 2.4 mmol) and 1,1,3,3-tetramethyl guanidine (330 µL, 2.6 mmol) were added, and the mixture was heated at 70°C overnight under a nitrogen atmosphere. The reaction mixture was diluted with CH_2Cl_2 (40 mL) and washed sequentially with acetic acid (0.1 M, 50 mL), saturated $NaHCO_3$ solution (50 mL), and brine (50 mL). The organic phase was dried over Na_2SO_4, concentrated *in vacuo*, and the residue was purified by silica gel chromatography to yield 7-[1-(4,5-dimethoxy-2-nitrophenyl)-2,2-dimethyl-propyloxy]methyl-7-deaza-2'-deoxyguanosine **35** (42 mg, 68%) as a 1:1 mixture of two diastereomers.

^{1}H NMR (400 MHz, DMSO-d_6) for diastereomers: δ 10.33 (br s, 1 H, D_2O exchangable, NH), 7.47 and 7.44 (2 s, 1 H, Ph-H), 7.16 and 7.15 (2 s, 1 H, Ph-H), 6.83 and 6.82 (2 s, 1 H, H-8), 6.22 (m, 3 H, 2 H D_2O exchangable, NH_2 and H-1'), 5.18 (br s, 1 H, D_2O exchangable, 3'-OH), 5.06 and 5.04 (2 s, 1 H, Ph-CH), 4.83 (t, 1 H, D_2O exchangable, 5'-OH), 4.44-4.23 (m, 3 H, 7-CH_2 and H-3'),

3.82 (4 s, 6 H, $OCH_3 \times 2$), 3.70 (m, 1 H, H-4’), 3.42 (m, 2 H, H-5’), 2.22 (m, 1 H, H-2’a), 2.01 (m, 1 H, H-2’b), 0.77 and 0.75 (2 s, 9 H, $(CH_3)_3$).

Compound **35** (40 mg, 0.073 mmol) was phosphorylated with $POCl_3$ (14 µL, 0.15 mmol) and proton sponge (31 mg, 0.15 mmol) in trimethylphosphate (0.35 mL) at 0°C for two hours under a nitrogen atmosphere. A solution of bis-tri-*n*-butylammonium pyrophosphate (237 mg, 0.50 mmol) and tri-*n*-butylamine (100 µL) in anhydrous DMF (1.0 mL) was added. After 10 min of stirring, triethylammonium bicarbonate buffer (0.1 M, pH 7.5; 10 mL) was added. The reaction was stirred at room temperature for one hour and then concentrated *in vacuo*. The residue was dissolved in 20% aqueous acetonitrile (20 mL), filtered, and purified by anion exchange chromatography. The fractions containing triphosphate were combined and lyophilized to give 7-[1-(4,5-dimethoxy-2-nitrophenyl)-2,2-dimethyl-propyloxy]methyl-7-deaza-2’-deoxyguanosine-5’-triphosphate **dG.V.d** as a 1:1 mixture of two diastereomers, which were separated using RP-HPLC to yield the single diastereomers **dG.V.d ds1** and **dG.V.d ds2**.

HRMS (ESI): For the molecular ion $C_{25}H_{35}N_5O_{18}P_3$ $[M-H]^-$, the calculated mass was 786.1190, and the observed mass was 786.1206.

7-[(S)-1-(5-Methoxy-2-nitrophenyl)-2,2-dimethyl-propyloxy]methyl-7-deaza-2'-deoxyguanosine-5'-triphosphate

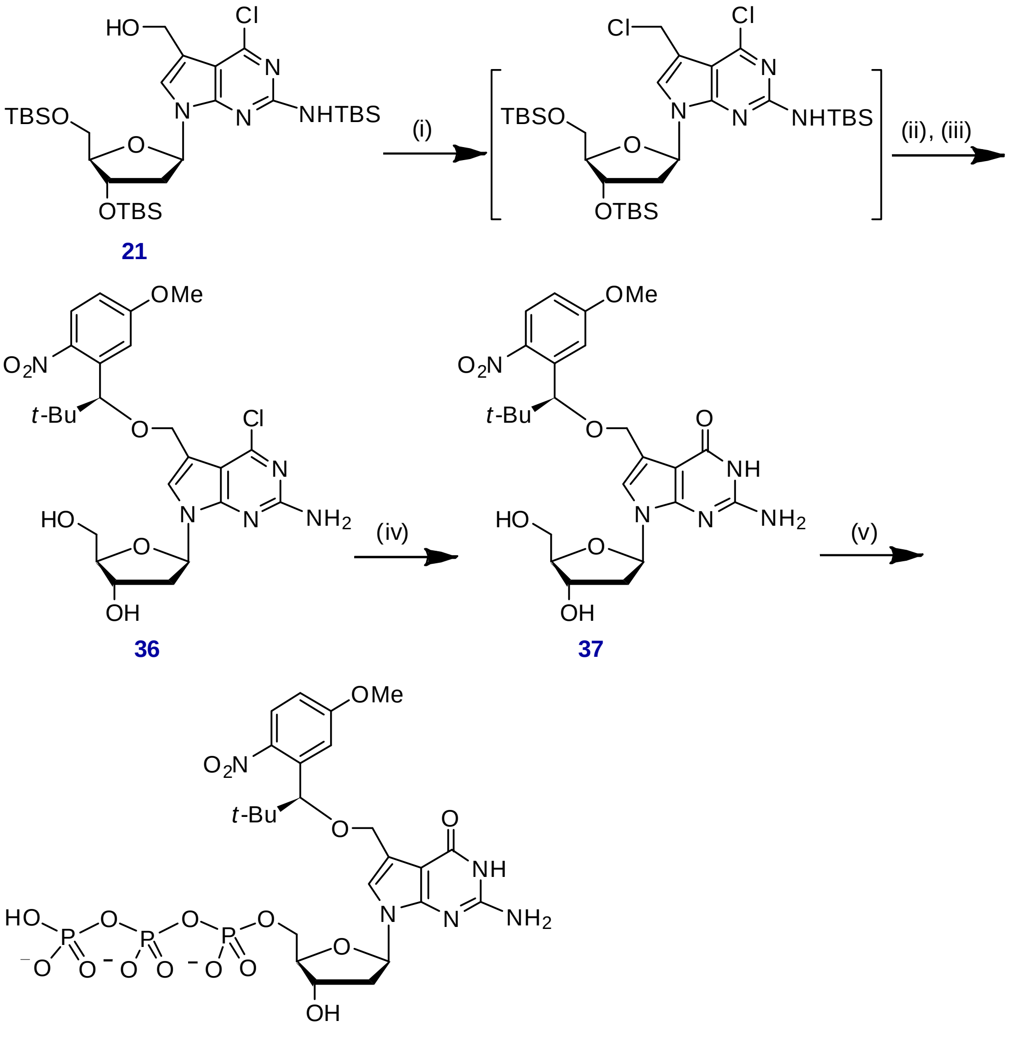

Scheme S18. Synthesis of 7-[(*S*)-1-(5-methoxy-2-nitrophenyl)-2,2-dimethyl-propyloxy]methyl-7-deaza-2'-deoxyguanosine-5'-triphosphate. *Reagents and conditions*: (*i*) MsCl, DMAP, CH_2Cl_2, 0°C; (*ii*) (*S*)-1-(5-methoxy-2-nitrophenyl)-2,2-dimethyl-1-propanol, 115°C; (*iii*) *n*-Bu_4NF, THF, room temperature, 27% for three steps; (*iv*) syn-pyridine-2-aldoxime, 1,1,3,3-tetramethyl guanidine, 1,4-dioxane/DMF, 70°C, 76%; (*v*) $POCl_3$, proton sponge, $(MeO)_3PO$, 0°C; $(n\text{-}Bu_3NH)_2H_2P_2O_7$, *n*-$Bu_3N$, DMF; 1 M $HNEt_3HCO_3$.

DMAP (224 mg, 1.8 mmol) and MsCl (106 µL, 1.4 mmol) were added to a solution of compound **21** (300 mg, 0.46 mmol) in anhydrous CH_2Cl_2 (5.0 mL) at 0°C under a nitrogen atmosphere. The reaction was stirred at 0°C for 10 min and diluted with CH_2Cl_2 (20 mL). The solution was applied on a short silica gel plug (2 × 3 cm) and was eluted quickly with a hexane/ethyl

acetate/triethylamine solvent system (volume ratio: 80/20/0.5). The eluent was concentrated *in vacuo*, and residue was mixed with (*S*)-1-(5-methoxy-2-nitrophenyl)-2,2-dimethyl-1-propanol (500 mg, 2.1 mmol). The mixture was heated at 115°C for 45 min under a nitrogen atmosphere, cooled to room temperature and dissolved in THF (10 mL) followed by addition of *n*-Bu_4NF (507 mg, 1.6 mmol). The mixture was stirred at room temperature for four hours and then concentrated *in vacuo*. The residue was dissolved in CH_2Cl_2 (20 mL) and washed with brine (30 mL), and the aqueous phase was extracted with CH_2Cl_2 (20 mL) two times. The combined organic phase was dried over Na_2SO_4, concentrated *in vacuo*, and the residue was purified by silica gel chromatography to yield 2-amino-6-chloro-9-[β-D-2'-deoxyribofuranosyl]-7-[(*S*)-1-(5-methoxy-2-nitrophenyl)-2,2-dimethyl-propyloxy]-methyl-7 deazapurine **36** (67 mg, 27% for three steps).

^{1}H NMR (400 MHz, $CDCl_3$): δ 7.82 (d, 1 H, *J* = 8.8 Hz, Ph-H), 7.16 (d, 1 H, *J* = 2.8 Hz, Ph-H), 6.90 (s, 1 H, H-8), 6.72 (dd, 1 H, *J* = 8.8 and 2.8 Hz, Ph-H), 6.12 (dd, 1 H, *J* = 9.2 and 6.0 Hz, H-1'), 5.22 (s, 1 H, Ph-CH), 5.15 (br s, 2 H, NH_2), 4.69-4.55 (m, 3 H, H-3'and 7-CH_2), 4.11 (m, 1 H, H-4'), 3.92 (m, 1 H, H-5'a), 3.82 (s, 3 H, OCH_3), 3. 73 (m, 1 H, H-5'b), 2.81 (m, 1 H, H-2'a), 2.21 (m, 1 H, H-2'b), 0.82 (s, 9 H, $(CH_3)_3$).

To a solution of compound **36** (65 mg, 0.12 mmol) in 1,4-dioxane (1.0 mL) and DMF (2.0 mL), syn-pyrimidine-2-aldoxime (292 mg, 2.4 mmol) and 1,1,3,3-tetramethyl guanidine (331 µL, 2.6 mmol) were added, and the mixture was heated at 70°C overnight under a nitrogen atmosphere. The reaction mixture was diluted with CH_2Cl_2 (20 mL) and washed sequentially with acetic acid (0.1 M, 30 mL), saturated $NaHCO_3$ solution (30 mL), and brine (30 mL). The organic phase was dried over Na_2SO_4, concentrated *in vacuo*, and the residue was purified by silica gel chromatography to yield 7-[(*S*)-1-(5-methoxy-2-nitrophenyl)-2,2-dimethyl-propyloxy]methyl-7-deaza-2'-deoxyguanosine **37** (48 mg, 76%).

^{1}H NMR (400 MHz, DMSO-d_6): δ 10.37 (br s, 1 H, D_2O exchangable, NH), 7.95 (d, 1 H, *J* = 9.2 Hz, Ph-H), 7.18 (d, 1 H, *J* = 2.8 Hz, Ph-H), 7.03 (dd, 1 H, *J* = 9.2 and 2.8 Hz, Ph-H), 6.84 (s, 1 H, H-8), 6.23 (m, 3 H, 2 H D_2O exchangable, NH_2 and H-1'), 5.20 (d, 1 H, D_2O exchangable, 3'-OH), 5.13 (s, 1 H, Ph-CH), 4.84 (t, 1 H, D_2O exchangable, 5'-OH), 4.48 (d, 1 H, *J* = 12.0 Hz, 7-CH_2a), 4.32 (d, 1 H, *J* = 12.0 Hz, 7-CH_2b), 4.27 (m, 1 H, H-3'), 3.88 (s, 3 H, OCH_3), 3.73 (m, 1 H, H-4'), 3.46 (m, 2 H, H-5'), 2.30 (m, 1 H, H-2'a), 2.05 (m, 1 H, H-2'b), 0.77 (s, 9 H, $(CH_3)_3$).

Compound **37** (10 mg, 0.02 mmol) was phosphorylated with $POCl_3$ (26 µL, 0.26 mmol) and proton sponge (8 mg, 0.04 mmol) in trimethylphosphate (0.3 mL) at 0°C for 6.5 hours under a nitrogen atmosphere. A solution of bis-tri-*n*-butylammonium pyrophosphate (237 mg, 0.50 mmol) and tri-*n*-butylamine (100 µL) in anhydrous DMF (1.0 mL) was added. After 10 min of stirring, triethylammonium bicarbonate buffer (0.1 M, pH 7.5; 10 mL) was added. The reaction was stirred at room temperature for one hour and then concentrated *in vacuo*. The residue was dissolved in 20% aqueous acetonitrile (10 mL), filtered, and purified by anion exchange chromatography. The fractions containing triphosphate were combined and lyophilized to give 7-[(*S*)-1-(5-methoxy-2-nitrophenyl)-2,2-dimethyl-propyloxy]methyl-7-deaza-2'-deoxyguanosine-5'-triphosphate **dG.VI**, which was further purified using RP-HPLC. The retention time of **dG.VI** was identical to that of **dG.V.c ds2** by RP-HPLC analysis under the same condition (data not shown).

HRMS (ESI): For the molecular ion $C_{24}H_{33}N_5O_{17}P_3$ $[M\text{-}H]^-$, the calculated mass was 756.1084, and the observed mass was 756.1101.

VII. Synthesis of 5-HOMe-2'-deoxyuridine triphosphate analog

5-[(S)-1-(5-Methoxy-2-nitrophenyl)-2,2-dimethyl-propyloxy]methyl-2'-deoxyuridine-5'-triphosphate

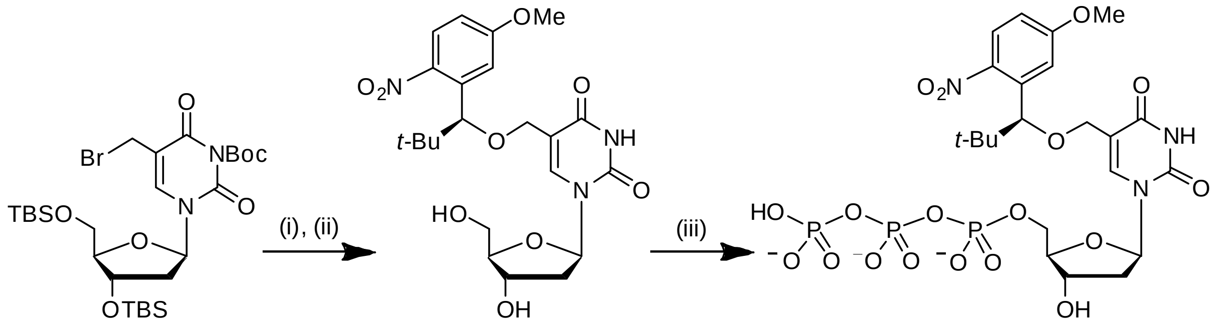

Scheme S19. Synthesis of 5-[(*S*)-1-(5-methoxy-2-nitrophenyl)-2,2-dimethyl-propyloxy]methyl-2'-deoxyuridine-5'-triphosphate. *Reagents and conditions*: (*i*) (*S*)-1-(5-methoxy-2-nitrophenyl)-2,2-dimethyl-1-propanol, 110°C; (*ii*) NH_4F, MeOH, 50°C, 56% for two steps; (*iii*) $POCl_3$, proton sponge, $(MeO)_3PO$, 0°C; $(n\text{-}Bu_3NH)_2H_2P_2O_7$, n-Bu_3N, DMF; 1 M $HNEt_3HCO_3$.

Compound **38**[2] (315 mg, 0.49 mmol) and (*S*)-1-(5-methoxy-2-nitrophenyl)-2,2-dimethyl-1-propanol (490 mg, 2.1 mmol) were heated at 110°C for 45 min under a nitrogen atmosphere.

The mixture was cooled down to room temperature, dissolved in MeOH (10 mL), and followed by addition of NH_4F (400 mg, 11 mmol). The mixture was stirred at 50°C for 12 hours, concentrated *in vacuo*, dissolved in CH_2Cl_2 (50 mL), and washed with brine (50 mL). The organic phase was dried over Na_2SO_4, concentrated *in vacuo*, and the residue was purified by silica gel chromatography to yield 5-[(*S*)-1-(5-methoxy-2-nitrophenyl)-2,2-dimethyl-propyloxy]methyl-2'-deoxyuridine **39** (130 mg, 56%).

^{1}H NMR (400 MHz, $CDCl_3$): δ 9.14 (br s, 1 H, NH), 7.90 (d, 1 H, *J* = 9.2 Hz, Ph-H), 7.67 (s, 1 H, H-6), 7.17 (d, 1 H, *J* = 2.8 Hz, Ph-H), 6.84 (dd, 1 H, *J* = 9.2 and 2.8 Hz, Ph-H), 6.18 (t, 1 H, *J* = 6.4 Hz, H-1'), 5.22 (s, 1 H, Ph-CH), 4.56 (m, 1 H, H-3'), 4.24 (d, 1 H, *J* = 12.4 Hz, 5-CH_2a), 4.15 (d, 1 H, *J* = 12.4 Hz, 5-CH_2b), 4.00 (m, 1 H, H-4'), 3.90 (m, 1 H, H-5'a), 3.88 (s, 3 H, OCH_3), 3.81 (m, 1 H, H-5'b), 2.35 (m, 2 H, H-2), 0.83 (s, 9 H, $C(CH_3)_3$).

Compound **39** (30 mg, 0.065 mmol) was phosphorylated with $POCl_3$ (9 µL, 0.097 mmol) and proton sponge (28 mg, 0.13 mmol) in trimethylphosphate (0.35 mL) at 0°C for one hour under a nitrogen atmosphere. A solution of tri-*n*-butylammonium pyrophosphate (147 mg, 0.32 mmol) and tri-*n*-butylamine (64 µL) in anhydrous DMF (0.64 mL) was added. After 10 min of stirring, triethylammonium bicarbonate buffer (0.1 M, pH 7.5; 10 mL) was added. The reaction was stirred at room temperature for one hour and then concentrated *in vacuo*. The residue was dissolved in 20% aqueous acetonitrile (10 mL), filtered, and purified by anion exchange chromatography. The fractions containing triphosphate were combined and lyophilized to yield 5-[(*S*)-1-(5-methoxy-2-nitrophenyl)-2,2-dimethyl-propyloxy]methyl-2'-deoxyuridine-5'-triphosphate **dU.VI**, which was further purified using RP-HPLC.

HRMS (ESI): For the molecular ion $C_{22}H_{31}N_3O_{18}P_3$ $[M-H]^-$, the calculated mass was 718.0815, and the observed mass was 718.0824.

VIII. Synthesis of 5-HOMe-2’-deoxycytidine triphosphate analogs

5-(2-nitrobenzyloxy)methyl-2'-deoxycytidine-5’-triphosphate

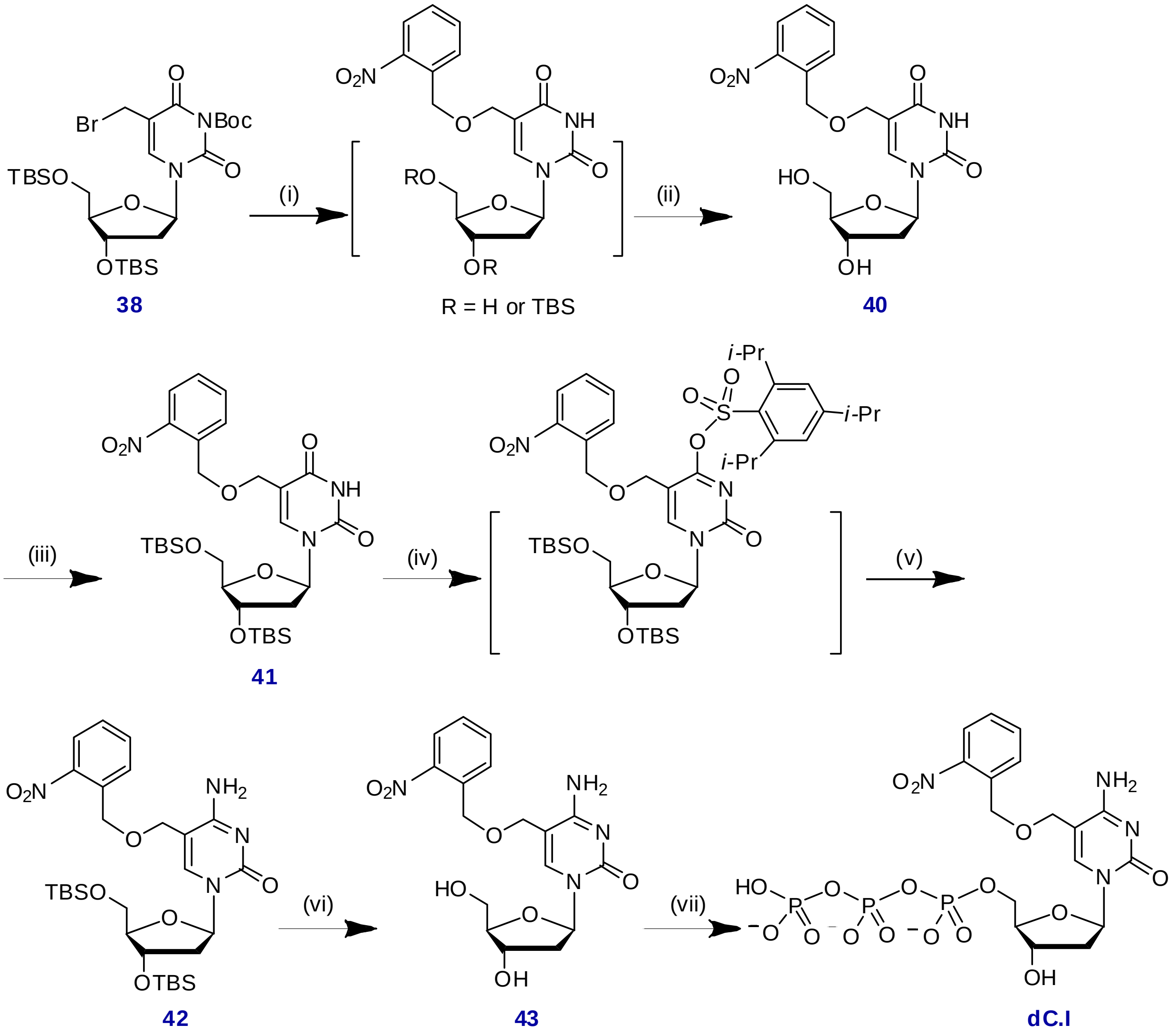

Scheme S20. Synthesis of 5-(2-nitrobenzyloxy)methyl-2'-deoxycytidine-5’-triphosphate. *Reagents and conditions*: (*i*) 2-nitrobenzyl alcohol, 110°C; (*ii*) *n*-Bu_4NF, THF, room temperature, 53% for two steps; (*iii*) TBSCl, imidazole, DMF, room temperature, 80%; (*iv*) 2,4,6-triisopropylbenzenesulfonyl chloride, DMAP, Et_3N, CH_2Cl_2, room temperature; (*v*) NH_3, 1,4-dioxane, 90°C, 69% for two steps; (*vi*) *n*-Bu_4NF, THF, room temperature, 96%; (*vii*) $POCl_3$, proton sponge, $(MeO)_3PO$, 0°C; $(n\text{-}Bu_3NH)_2H_2P_2O_7$, *n*-$Bu_3N$, DMF; 1 M $HNEt_3HCO_3$.

Compound **38** (300 mg, 0.46 mmol) and 2-nitrobenzyl alcohol (500 mg, 3.3 mmol) were heated at 110°C for 45 min under a nitrogen atmosphere. The mixture was cooled to room temperature, dissolved in THF (20 mL) followed by addition of *n*-Bu_4NF (362 mg, 1.2 mmol). The mixture was stirred at room temperature for four hours, concentrated *in vacuo*, and the residue

was purified by silica gel chromatography to yield 5-(2-nitrobenzyloxy)methyl-2'-deoxyuridine **40**[2](95 mg, 53%).

^{1}H NMR (400 MHz, $CDCl_3$): δ 8.45 (br s, 1 H, NH), 8.05 (s, 1 H, H-6), 8.02 (d, *J* = 8.0 Hz, 1 H, Ph-H), 7.80 (d, 1 H, *J* = 8.0 Hz, Ph-H), 7.69 (t, 1 H, *J* = 8.0 Hz, Ph-H), 7.43 (t, 1 H, *J* = 8.0 Hz, Ph-H), 6.21 (t, 1 H, *J* = 6.0 Hz, H-1'), 4.94 (dd, *J* = 14.4 Hz, 2 H, Ph-CH_2), 4.66 (m, 1 H, H-3'), 4.35 (s, 2 H, 5-CH_2), 3.95 (m, 3 H, H-4' and H-5'), 2.42 (m, 1 H, H-2'a), 2.30 (m, 1 H, H-2'b).

To a solution of compound **40**[2] (70 mg, 0.18 mmol) in anhydrous DMF (2.0 mL), TBSCl (60 mg, 0.40 mmol) and imidazole (54 mg, 0.80 mmol) were added. The mixture was stirred at room temperature overnight under a nitrogen atmosphere, concentrated *in vacuo*, dissolved in CH_2Cl_2 (20 mL), and washed with saturated $NaHCO_3$ solution (30 mL). The organic and aqueous phases were separated, and the aqueous phase was extracted with CH_2Cl_2 (20 mL) two times. The combined organic phase was dried with Na_2SO_4, concentrated *in vacuo*, and the residue was purified by silica gel chromatography to yield 3',5'-*O*-bis-(*tert*-butyldimethylsilyl)-5-(2-nitrobenzyloxy)-methyl-2'-deoxyuridine **41** (90 mg, 80%).

^{1}H NMR (400 MHz, $CDCl_3$): δ 8.04 (d, *J* = 8.0 Hz, 1 H, Ph-H), 7.98 (br s, 1 H, NH), 7.80 (d, 1 H, *J* = 8.0 Hz, Ph-H), 7.74 (s, 1 H, H-6), 7.64 (q, 1 H, *J* = 8.0 Hz, Ph-H), 7.44 (t, 1 H, *J* = 8.0 Hz, Ph-H), 6.21 (q, 1 H, *J* = 6.0 Hz, H-1'), 4.95 (s, 2 H, Ph-CH_2), 4.41 (m, 1 H, H-3'), 4.34 (dd, 2 H, *J* = 11.6 Hz, 5-CH_2), 3.96 (m, 1 H, H-4'), 3.79 (m, 2 H, H-5'), 2.29 (m, 1 H, H-2'a), 2.05 (m, 1 H, H-2'b), 0.89 (2 s, 18 H, $C(CH_3)_3$), 0.08 (4 s, 12 H, CH_3).

2,4,6-Triisopropyl benzenesulfonyl chloride (176 mg, 0.59 mmol) was added to a solution of compound **41** (85 mg, 0.14 mmol), DMAP (19 mg, 0.16 mmol), and triethylamine (0.18 mL, 1.3 mmol) in anhydrous CH_2Cl_2 (5.0 mL). The mixture was stirred at room temperature overnight under a nitrogen atmosphere, concentrated *in vacuo*, and the residue was dissolved in a solution of NH_3 in 1,4-dioxane (0.5 M, 15 mL). The mixture was transferred into a sealed tube and was heated at 90°C overnight. The mixture was cooled to room temperature, concentrated *in vacuo*, dissolved in CH_2Cl_2 (30 mL), and washed with brine (30 mL). The organic and aqueous phases were separated, and the aqueous phase was extracted with CH_2Cl_2 (30 mL) two times. The combined organic phase was dried over Na_2SO_4, concentrated *in vacuo*, and the residue was purified by silica gel column chromatography to yield 3',5'-*O*-bis-(*tert*-butyldimethylsilyl)-5-(2-nitrobenzyloxy)methyl-2'-deoxycytidine **42** (60 mg, 69% for two steps).

^{1}H NMR (400 MHz, $CDCl_3$): δ 8.08 (d, *J* = 8.0 Hz, 1 H, Ph-H), 7.81 (s, 1 H, H-6), 7.65 (m, 2 H, Ph-H), 7.64 (q, 1 H, *J* = 8.0 Hz, Ph-H), 7.49 (m, 1 H, Ph-H), 6.29 (t, 1 H, *J* = 6.4 Hz, H-1'), 5.75 (br s, 1 H, NH_2), 4.85 (dd, 2 H, *J* = 13.6 Hz, Ph-CH_2), 4.41 (s, 2 H, 5-CH_2), 4.34 (m, 1 H, H-3'), 3.95 (m, 1 H, H-4'), 3.89 (dd, 1 H, *J* = 2.8 Hz, H-5'a), 3.76 (dd, 1 H, *J* = 2.8 Hz, H-5'b), 2.46 (m, 1 H, H-2'a), 1.98 (m, 1 H, H-2'b), 0.92 and 0.89 (2 s, 18 H, $C(CH_3)_3$), 0.11-0.08 (4 s, 12 H, CH_3).

To a solution of compound **42** (55 mg, 0.09 mmol) in THF (10 mL), *n*-Bu_4NF (63 mg, 0.20 mmol) was added. The mixture was stirred at room temperature for four hours and concentrated *in vacuo*, and the residue was purified by silica gel column chromatography to yield 5-(2-nitrobenzyloxy)methyl-2'-deoxycytidine **43** (34 mg, 96%).

^{1}H NMR (400 MHz, DMSO-d_6): δ 8.05 (d, *J* = 8.0 Hz, 1 H, Ph-H), 7.89 (s, 1 H, H-6), 7.74 (m, 2 H, Ph-H), 7.55 (m, 1 H, Ph-H), 7.39 (br s, 1 H, D_2O exchangeable, NH_2), 6.74 (br s, 1 H, D_2O exchangeable, NH_2), 6.12 (t, 1 H, *J* = 6.4 Hz, H-1'), 5.21 (br s, 1 H, D_2O exchangeable, 3'-OH), 4.99 (br s, 1 H, D_2O exchangeable, 5'-OH), 4.81 (s, 2 H, Ph-CH_2), 4.30 (dd, 2 H, *J* = 11.6 Hz, 5-CH_2), 4.20 (m, 1 H, H-3'), 3.76 (m, 1 H, H-4'), 3.55 (m, 2 H, H-5'), 2.11 (m, 1 H, H-2'a), 1.95 (m, 1 H, H-2'b).

Compound **43** (32 mg, 0.081 mmol) was phosphorylated with $POCl_3$ (30 µL, 0.32 mmol) and proton sponge (35 mg, 0.16 mmol) in trimethylphosphate (0.35 mL) at $0^{o}C$ for three hours under a nitrogen atmosphere. A solution of tri-*n*-butylammonium pyrophosphate (237 mg, 0.50 mmol) and tri-*n*-butylamine (100 µL) in anhydrous DMF (1.0 mL) was added. After 10 min of stirring, triethylammonium bicarbonate buffer (0.1 M, pH 7.5; 10 mL) was added. The reaction was stirred at room temperature for one hour and then concentrated *in vacuo*. The residue was dissolved in 20% aqueous acetonitrile (20 mL), filtered, and purified by anion exchange chromatography. The fractions containing triphosphate were combined and lyophilized to give 5-(2-nitrobenzyloxy)methyl-2'-deoxycytidine-5'-triphosphate **dC.I**, which was further purified using RP-HPLC.

HRMS (ESI): For the molecular ion $C_{17}H_{22}N_4O_{16}P_3$ $[M-H]^-$, the calculated mass was 631.0244, and the observed mass was 631.0258.

5-[(S)-1-(2-nitrophenyl)-2,2-dimethyl-propyloxy]methyl-2'-deoxycytidine-5'-triphosphate

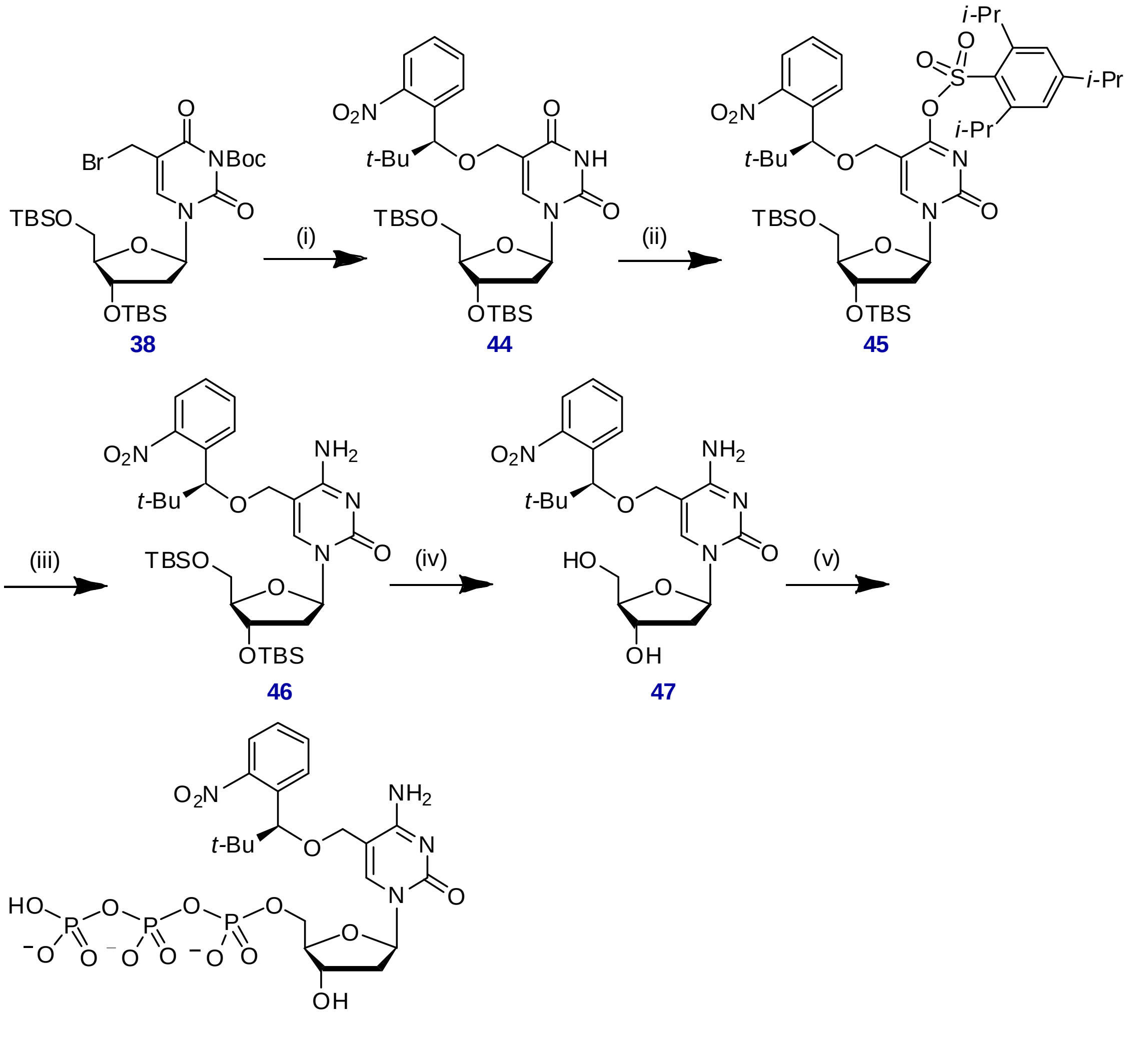

Scheme S21. Synthesis of 5-[(*S*)-1-(2-nitrophenyl)-2,2-dimethyl-propyloxy]methyl-2'-deoxycytidine-5'-triphosphate. *Reagents and conditions*: (i) (*S*)-1-(2-nitrophenyl)-2,2-dimethyl-1-propanol, 110°C, 21%; (ii) 2,4,6-triisopropylbenzenesulfonyl chloride, DMAP, Et_3N, CH_2Cl_2, room temperature, 31%; (iii) NH_3, 1,4-dioxane, 90°C, 91%; (iv) *n*-Bu_4NF, THF, room temperature, 82%; (v) $POCl_3$, proton sponge, $(MeO)_3PO$, 0°C; (*n*-$Bu_3NH)_2H_2P_2O_7$, *n*-Bu_3N, DMF; 1 M $HNEt_3HCO_3$.

Compound **38**[2] (520 mg, 0.80 mmol) and (*S*)-1-(2-nitrophenyl)-2,2-dimethyl-1-propanol (580 mg, 2.8 mmol) were heated at 110°C for one hour under a nitrogen atmosphere. The mixture was cooled down to room temperature, dissolved in a minimum amount of ethyl acetate, and purified by silica gel chromatography to yield 3',5'-*O*-bis-(*tert*-butylsimethylsilyl)-5-[(*S*)-1-(2-nitrophenyl)-2,2-dimethyl-propyloxy]methyl-2'-deoxyuridine **44** (115 mg, 21%). (3' or 5')-*O*-(*tert*-butylsimethylsilyl)-5-[(*S*)-1-(2-nitrophenyl)-2,2-dimethyl-propyloxy]methyl-

2'-deoxyuridine (78 mg, 17%) and 5-[(*S*)-1-(2-nitrophenyl)-2,2-dimethyl-propyloxy]methyl-2'-deoxyuridine (16 mg, 4%) was also obtained from the reaction.

^{1}H NMR (400 MHz, $CDCl_3$): δ 8.97 (s, 1 H, NH), 7.76 (d, 2 H, *J* = 8.0 Hz, Ph-H), 7.60 (m, 2 H, Ph-H and H-6), 7.41 (s, 1 H, Ph-H), 6.29 (dd, 1 H, *J* = 6.0 and 7.6 Hz, H-1'), 4.97 (s 1 H, Ph-CH), 4.42 (m, 1 H, H-3'), 4.28 (AB d, 1 H, *J* = 12.0 Hz, 5-CH_2a), 4.06 (AB d, 1 H, *J* = 12.0 Hz, 5-CH_2b), 3.92 (m, 1 H, H-4'), 3.76 (m, 2 H, H-5'), 2.30 (m, 1 H, H-2'a), 2.05 (m, 1 H, H-2'b), 0.95 (s, 9 H, $(CH_3)_3$CSi), 0.90 (s, 9 H, $(CH_3)_3$CSi), 0.83 (s, 9 H, $(CH_3)_3$C), 0.12 (s, 3 H, CH_3Si), 0.09 (s, 3 H, CH_3Si), 0.07 (s, 3 H, CH_3Si), 0.06 (s, 3 H, CH_3Si).

2,4,6-Triisopropyl benzenesulfonyl chloride (61 mg, 0.20 mmol) was added to a solution of compound **44** (110 mg, 0.16 mmol), DMAP (20 mg, 0.17 mmol), and triethylamine (63 µL, 0.45 mmol) in anhydrous CH_2Cl_2 (3.0 mL). The mixture was stirred at room temperature for 36 hours under a nitrogen atmosphere, concentrated *in vacuo*, and the residue was purified by silica gel column chromatography to give 3',5'-*O*-bis-(*tert*-butylsimethylsilyl)-5-[(*S*)-1-(2-nitrophenyl)-2,2-dimethyl-propyloxy]methyl-*O*^{4}-(2,4,6-triisopropylbenzenesulfonyl)-2'-deoxyuridine **45** (47 mg, 31%).

^{1}H NMR (500 MHz, $CDCl_3$): δ 8.08 (s, 1 H, H-6), 7.80 (dd, 1 H, *J* = 1.2 and 8.0 Hz, Ph-H), 7.78 (dd, 1 H, *J* = 1.6 and 8.0 Hz, Ph-H), 7.67 (m, 1 H, Ph-H), 7.46 (m, 1 H, Ph-H), 7.20 (s, 2 H, Ph-H), 6.09 (t, 1 H, *J* = 6.4 Hz, H-1'), 4.98 (s, 1 H, Ph-CH), 4.35 (m, 1 H, H-3'), 4.25 (AB d, 1 H, *J* = 11.6 Hz, 5-CH_2a), 4.11 (AB d, 1 H, *J* = 11.6 Hz, 5-CH_2b), 3.97 (m, 1 H, H-4'), 3.79 (dd, 1 H, *J* = 3.6 and 11.6 Hz, H-5'a), 3.74 (dd, 1 H, *J* = 11.6 and 3.6 Hz, H-5'b), 2.90 (m, 1 H, CH), 2.50 (m, 2 H, H-2'), 1.98 (m, 2 H, CH), 1.31 - 1.22 (m, 18 H, $(CH_3)_2$CH x 3), 0.88 (2 s, 18 H, $(CH_3)_3$CSi x 2), 0.87 (s, 9 H, $(CH_3)_3$C), 0.07 (s, 6 H, $(CH_3)_2$Si), 0.06 (s, 6 H, $(CH_3)_2$Si).

A solution of NH_3 in 1,4-dioxane (0.5 M, 2.0 mL) was added to a solution of compound **45** (47 mg, 0.05 mmol) in anhydrous 1,4-dioxane (2.0 mL). The mixture was transferred into a sealed tube and was heated at 90°C for ten hours. The mixture was cooled to room temperature, concentrated *in vacuo* and the residue was purified by silica gel column chromatography to yield 3',5'-O-bis-(*tert*-butyldimethylsilyl)-5-[(*S*)-1-(2-nitrophenyl)-2,2-dimethyl-propyloxy]-methyl-2'-deoxycytidine **46** (31 mg, 91%).

^{1}H NMR (400 MHz, $CDCl_3$): δ 7.67 (m, 3 H, Ph-H), 7.53 (s, 1 H, H-6), 7.45 (m, 1 H, Ph-H), 6.30 (t, 1 H, *J* = 6.6 Hz, H-1'), 5.72 (br s, 2 H, NH_2), 4.88 (s, 1 H, Ph-CH), 4.32 (m, 1 H, H-3'), 4.28 (AB d, 1 H, *J* = 12.8 Hz, 5-CH_2a), 4.08 (AB d, 1 H, *J* = 12.8 Hz, 5-CH_2b), 3.87 (m, 1 H, H-4'), 3.74 (dd, 1 H, *J* = 3.6 and 14.8 Hz, H-5'a), 3.66 (dd, 1 H, *J* = 3.6 and 11.3 Hz, H-5'b), 2.41 (m, 1 H, H-2'a), 2.03

(m, 1 H, H-2’b), 0.90 (s, 9 H, $(CH_3)_3CSi$), 0.87 (s, 9 H, $(CH_3)_3CSi$), 0.83 (s, 9 H, $C(CH_3)_3$), 0.09 (2 s, 6 H, $(CH_3)_2Si$), 0.06 (2 s, 6 H, $(CH_3)_2Si$).

A solution of *n*-Bu_4NF (28 mg, 0.09 mmol) in THF (1.0 mL) was added to a solution of compound **46** (20 mg, 0.03 mmol) in THF (2.0 mL). The mixture was stirred at room temperature for 30 min and concentrated *in vacuo*, and the residue was purified by silica gel column chromatography to yield 5-[(*S*)-1-(2-nitrophenyl)-2,2-dimethyl-propyloxy]methyl-2'-deoxycytidine **47** (11 mg, 82%).

^{1}H NMR (400 MHz, CD_3OD): δ 7.87 (s, 1 H, H-6), 7.82 (dd, 1 H, *J* = 1.2 and 8.4 Hz, Ph-H), 7.76 (dd, 1 H, *J* = 1.6 and 8.0 Hz, Ph-H), 7.68 (m, 1 H, Ph-H), 7.51 (m, 1 H, Ph-H), 6.23 (t, 1 H, *J* = 6.6 Hz, H-1’), 4.94 (s, 1 H, Ph-CH), 4.44 (AB d, 1 H, *J* = 13.2 Hz, 5-CH_2a), 4.34 (m, 1 H, H-3’), 4.11 (AB d, 1 H, *J* = 13.2 Hz, 5-CH_2b), 3.88 (m, 1 H, H-4’), 3.71 (dd, 1 H, *J* = 3.2 and 12.0 Hz, H-5’a), 3.63 (dd, 1 H, *J* = 4.0 and 12.0 Hz, H-5’b), 2.35 (m, 1 H, H-2’a), 2.14 (m, 1 H, H-2’b), 0.80 (s, 9 H, $C(CH_3)_3$).

Compound **47** (11 mg, 0.025 mmol) was phosphorylated with $POCl_3$ (7 µL, 0.075 mmol) and proton sponge (11 mg, 0.05 mmol) in trimethylphosphate (0.3 mL) at 0°C for three hours under a nitrogen atmosphere. A solution of tri-*n*-butylammonium pyrophosphate (59 mg, 0.125 mmol) and tri-*n*-butylamine (30 µL) in anhydrous DMF (0.25 mL) was added. After 5 min of stirring, triethylammonium bicarbonate buffer (1 M, pH 7.5; 5.0 mL) was added. The reaction was stirred at room temperature for one hour and then lyophilized to dryness. The residue was dissolved in water (5.0 mL), filtered, and purified by anion exchange chromatography. The fractions containing triphosphate were combined and lyophilized to give 5-[(*S*)-1-(2-nitrophenyl)-2,2-dimethyl-propyloxy]methyl-2'-deoxycytidine-5’-triphosphate **dC.V**, which was further purified using by RP-HPLC.

HRMS (ESI): For the molecular ion $C_{21}H_{30}N_4O_{16}P_3$ [M-H]$^-$, the calculated mass was 687.0870, and the observed mass was 687.0873.

5-[(S)-1-(5-Methoxy-2-nitrophenyl)-2,2-dimethyl-propyloxy]methyl-2'-deoxycytidine-5'-triphosphate

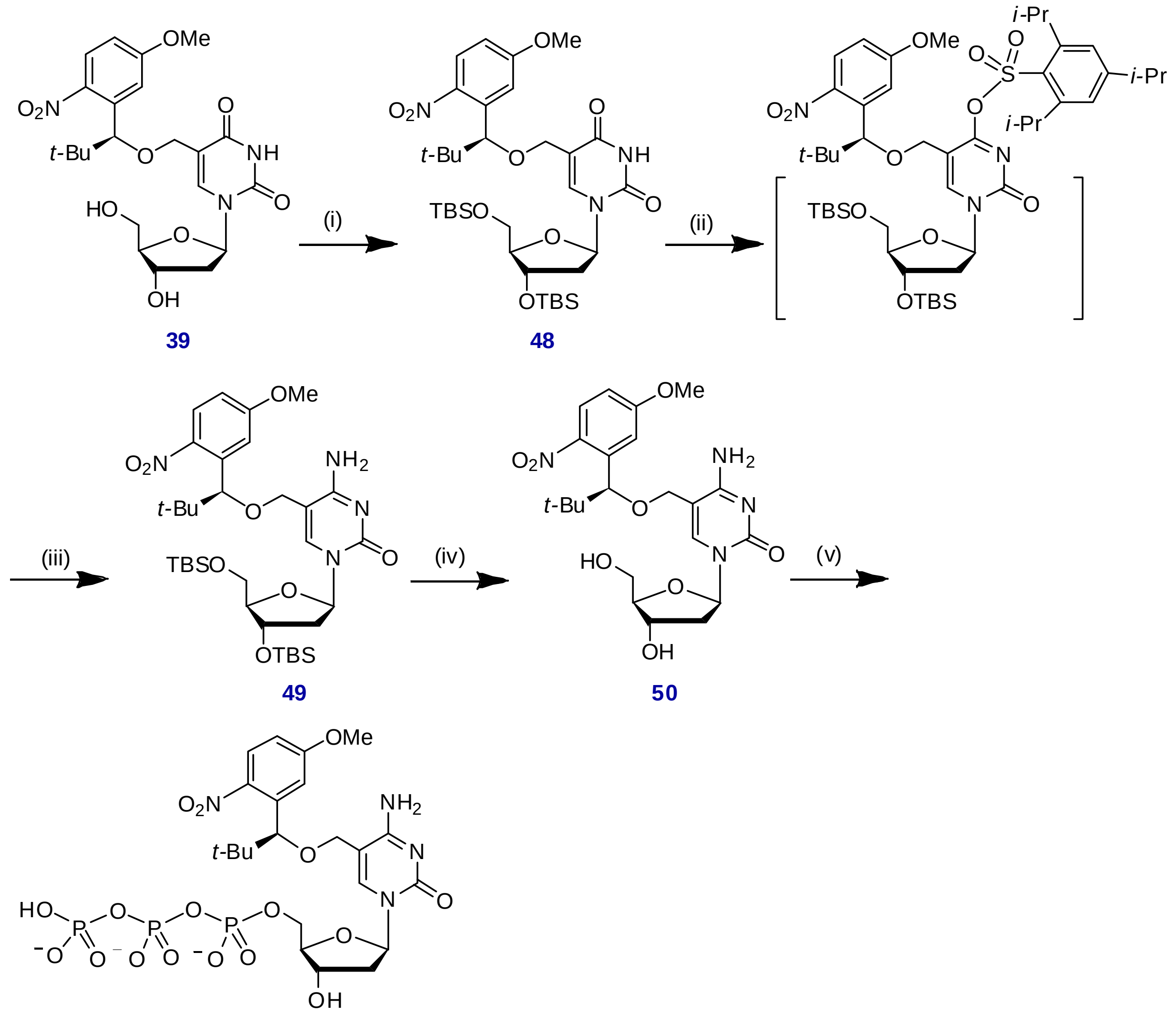

dC.VI

Scheme S22. Synthesis of 5-[(*S*)-1-(5-methoxy-2-nitrophenyl)-2,2-dimethyl-propyloxy]methyl-2'-deoxycytidine-5'-triphosphate. *Reagents and conditions*: (*i*) TBSCl, imidazole, DMF, room temperature, 70%; (*ii*) 2,4,6-triisopropylbenzenesulfonyl chloride, DMAP, Et_3N, CH_2Cl_2, room temperature; (*iii*) NH_3, 1,4-dioxane, 90°C, 65% for two steps; (*iv*) *n*-Bu_4NF, THF, room temperature, 82%; (*v*) $POCl_3$, proton sponge, $(MeO)_3PO$, 0°C; $(n\text{-}Bu_3NH)_2H_2P_2O_7$, *n*-$Bu_3N$, DMF; 1 M $HNEt_3HCO_3$.

To a solution of compound **39** (235 mg, 0.49 mmol) in anhydrous DMF (3.0 mL), TBSCl (320 mg, 0.8 mmol) and imidazole (109 mg, 1.6 mmol) were added. The mixture was stirred at room temperature for six hours, concentrated *in vacuo*, dissolved in CH_2Cl_2 (20 mL), and washed with saturated $NaHCO_3$ solution (50 mL). The organic and aqueous phases were separated, and the aqueous phase was extracted with CH_2Cl_2 (30 mL) three times. The combined organic phase

was dried with Na_2SO_4, concentrated *in vacuo*, and the residue was purified by silica gel chromatography to yield 3',5'-*O*-bis-(*tert*-butyldimethylsilyl)-5-[(*S*)-1-(5-methoxy-2-nitrophenyl)-2,2-dimethyl-propyloxy]methyl-2'-deoxyuridine **48** (245 mg, 70%).

^{1}H NMR (400 MHz, CDCl$_3$): δ 8.00 (br s, 1 H, NH), 7.88 (d, *J* = 9.2 Hz, 1 H, Ph-H), 7.60 (s, 1 H, H-6), 7.22 (d, 1 H, *J* = 2.8 Hz, Ph-H), 6.84 (dd, 1 H, *J* = 2.8 and 8.0 Hz, Ph-H), 6.25 (dd, 1 H, *J* = 5.6 and 8.0 Hz, H-1'), 5.23 (s, 1 H, Ph-CH), 4.40 (m, 1 H, H-3'), 4.26(d, 1 H, *J* = 12 Hz, 5-CH_2a), 4.11 (d, 1 H, *J* = 12 Hz,5-CH_2b), 3.89 (m, 4 H, OCH_3 and H-4'), 3.78 (m, 2 H, H-5'), 2.27 (m, 1 H, H-2'a), 2.04 (m, 1 H, H-2'b), 0.90 and 88 (2 s, 18 H, $SiC(CH_3)_3$), 0.84 (s, 9 H, $C(CH_3)_3$), 0.08 (3 s, 12 H, CH_3).

2,4,6-Triisopropyl benzenesulfonyl chloride (363 mg, 1.2 mmol) was added to a solution of compound **48** (170 mg, 0.24 mmol), DMAP (32 mg, 0.26 mmol), and triethylamine (0.34 mL, 2.4 mmol) in anhydrous CH_2Cl_2 (8.0 mL). The mixture was stirred at room temperature overnight under a nitrogen atmosphere, concentrated *in vacuo*, and the residue was dissolved in a solution of NH_3 in 1,4-dioxane (0.5 M, 20 mL). The mixture was transferred into a sealed tube and was heated at 90°C overnight. The mixture was cooled to room temperature, concentrated *in vacuo*, dissolved in CH_2Cl_2 (20 mL), and washed with brine (50 mL). The organic and aqueous phases were separated, and the aqueous phase was extracted with CH_2Cl_2 (30 mL) three times. The combined organic phase was dried over Na_2SO_4, concentrated *in vacuo*, and the residue was purified by silica gel column chromatography to yield 3',5'-*O*-bis-(*tert*-butyldimethylsilyl)-5-[(*S*)-1-(5-methoxy-2-nitrophenyl)-2,2-dimethyl-propyloxy]methyl-2'-deoxycytidine **49** (110 mg, 65% for two steps).

^{1}H NMR (400 MHz, DMSO-d$_6$): δ 7.96 (d, *J* = 8.8 Hz, 1 H, Ph-H), 7.50 (br s, 1 H, NH_2), 7.38 (s, 1 H, H-6), 7.08 (dd, 1 H, *J* = 2.8 and 8.8 Hz, Ph-H), 7.04 (d, 1 H, *J* = 2.8 Hz, Ph-H), 6.80 (br s, 1 H, NH_2), 6.13 (t, 1 H, *J* = 6.4 Hz, H-1'), 5.09 (s, 1 H, Ph-CH), 4.31 (m, 1 H, H-3'), 4.25 (d, 1 H, *J* = 12.8 Hz, 5-CH_2a), 4.08 (d, 1 H, *J* = 12.8 Hz, 5-CH_2b), 3.87(s, 3 H, OCH_3), 3.76 (m, 1 H, H-4'), 3.64 (m, 2 H, H-5'), 3.76 (dd, 1 H, *J* = 2.8 Hz, H-5'b), 2.10 (m, 1 H, H-2'a), 2.00 (m, 1 H, H-2'b), 0.87 (s, 9 H, $C(CH_3)_3$), 0.78 and 0.76 (2 s, 18 H, $SiC(CH_3)_3$), 0.07, 0.06, -0.01, and -0.04 (4 s, 12 H, $SiCH_3$).

To a solution of compound **49** (130 mg, 0.18 mmol) in THF (10 mL), *n*-Bu_4NF (141 mg, 0.44 mmol) was added. The mixture was stirred at room temperature for four hours, concentrated *in vacuo*, and the residue was purified by silica gel column chromatography to yield 5-[(*S*)-1-(5-methoxy-2-nitrophenyl)-2,2-dimethyl-propyloxy]methyl-2'-deoxycytidine **50** (72 mg, 82%).

^{1}H NMR (400 MHz, DMSO-d_6): δ 7.99 (d, *J* = 8.0 Hz, 1 H, Ph-H), 7.65 (s, 1 H, H-6), 7.42 (br s, 1 H, D_2O excahangable, NH_2a), 7.06 (m, 2 H, Ph-H), 6.72 (br s, 1 H, D_2O excahangable, NH_2b), 6.11 (t, 1 H, *J* = 6.4 Hz, H-1'), 5.17 (d, 1 H, D_2O excahangable, 3'-OH), 5.12 (s, 1 H, Ph-CH), 4.78 (t, 1 H, D_2O excahangable, 5'-OH), 4.25 (d, 1 H, *J* = 12.4 Hz, 5-CH_2a), 4.15 (m, 1 H, H-3'), 4.05 (d, 1 H, *J* = 12.4 Hz, 5-CH_2b), 3.87(s, 3 H, OCH_3), 3.72 (m, 1 H, H-4'), 3.44 (m, 2 H, H-5'), 3.76 (dd, 1 H, *J* = 2.8 Hz, H-5'b), 2.08 (m, 1 H, H-2'a), 1.95 (m, 1 H, H-2'b), 0.77 (s, 9 H, $C(CH_3)_3$).

Compound **50** (20 mg, 0.043 mmol) was phosphorylated with $POCl_3$ (24 µL, 0.26 mmol) and proton sponge (19 mg, 0.086 mmol) in trimethylphosphate (0.3 mL) at 0°C for six hours under a nitrogen atmosphere. A solution of tri-*n*-butylammonium pyrophosphate (237 mg, 0.50 mmol) and tri-*n*-butylamine (100 µL) in anhydrous DMF (1.0 mL) was added. After 10 min of stirring, triethylammonium bicarbonate buffer (0.1 M, pH 7.5; 10 mL) was added. The reaction was stirred at room temperature for one hour and then concentrated *in vacuo*. The residue was dissolved in 20% aqueous acetonitrile (20 mL), filtered, and purified by anion exchange chromatography. The fractions containing triphosphate were combined and lyophilized to give 5-[(*S*)-1-(5-methoxy-2-nitrophenyl)-2,2-dimethyl-propyloxy]methyl-2'-deoxycytidine-5'-triphosphate **dC.VI**, which was further purified using RP-HPLC.

HRMS (ESI): For the molecular ion $C_{22}H_{32}N_4O_{17}P_3$ $[M-H]^-$, the calculated mass was 719.0975, and the observed mass was 719.0983.

IX. UV photocleavage studies

As described in the main article, the rate of UV photocleavage is dependent on a number of experimental factors including light intensity.[6, 7] To compare the rates of photochemical cleavage between the nucleotide analogs described here, we developed a stringent protocol to deliver a daily light intensity output of 0.70 ± 0.01 W/cm^2 to samples, see below. Our custom-designed UV deprotector has been previously described in Wu et al.,[1] and the protocol implemented is described below.

UV deprotector set-up: The power supply was turned on for about 30 min prior to that of the lamp and recirculation bath as described by the manufacturer. The IR liquid filter was cooled to 9°C. Light intensity was determined using a model PM100 power meter (Thorlabs), a 1000 µm pinhole (Edmund Optics), a modified 0.5 mL Eppendorf tube cut in half, and a 3-axis manual translation stage (Newport), see Figure S3A. The half cut Eppendorf tube was positioned in front of the pinhole and power meter detector head to account for the geometric shape distortion of the light as it passes through a reaction solution. The translation stage was then used to align the tube/pinhole/detector device with the highest intensity from the arc beam.

Intensity adjustment to 0.7W/cm^2: To stabilize its output, the lamp was left on for one hour prior to intensity measurements. Thereafter, the measured power was adjusted by increasing the current from the power supply. In order to achieve intensity (I) of 0.70 W/cm^2, the measured power (P) was adjusted to ~5.5 mW, according to the equation:

$$I = \frac{P}{\pi \times r^2}$$

where r is the radius of pinhole. Power readings are recorded over a five minute period (in one second intervals) and were converted into intensity readings, which ranged over a six week period between 0.68 ± 0.01 and 0.72 ± 0.02 W/cm^2.

Beam alignment with the 0.5 mL tube holder: The modified Eppendorf tube, pin hole and power meter were then removed from the UV deprotector, and the rotating sample holder was installed with the height being 67.18 ± 0.25 mm. The beam was then focused by placing an 0.5 mL Eppendorf tube into the sample holder, the tube of which was modified with an internal alignment card to provide reference lines for volume heights of 10 µL and 20 µL, see Figure S3B.

The reference lines enabled the beam to be centered for a given reaction volume. Beam alignment was further verified by observing the mercury arc image of the lamp produced by the rear reflector. A second alignment card was placed into the rotating sample holder to view the image, which when properly aligned using the reflector would produce an inverted arc image on the arc gap itself. This step ensured that arc hotspots were not superimposed, which could cause overheating while maintaining a power output of ~5.5 mW. The speed of the rotating sample holder was adjusted within a range of 1,200 – 1,350 rpm using a Nova-Strobe DA Plus stroboscope (Monarch Instrument) by adjusting the motor's torque with an adjustment screw.

Photochemical cleavage assays: Nucleotide analogs were incorporated using 10 µL reactions, as described for the PEP assays, at a final concentration of 100 nM.[2] OligoTemplate-2, oligoTemplate-5, and oligoTemplate-4 each hybridized with BODIPY-FL labeled primer-1 were used for C^7-HOMedA, C^7-HOMedG, and HOMedU analogs, respectively. OligoTemplate-8 hybridized with BODIPY-FL labeled primer-3 was used to assay HOMedC analogs. Incorporated reactions were quenched with either 1 mM sodium azide solution; 1 mM sodium azide, 50 mM DTT solution; or reagents C-G (see key in Figure S2), exposed to 365 nm ultraviolet (UV) light for various time points using our UV deprotector, and then placed on ice. Ten µL of stop solution (98% deionized formamide; 10 mM Na_2EDTA, pH 8.0; 25 mg/mL Blue Dextran, MW 2,000,000) was added, and samples were analyzed using an AB model 377 DNA sequencer. Cleavage assays were performed in triplicate to calculate the average DT_{50} value ± 1SD, as described previously.[2]

Table S3 Photochemical cleavage rates of *Lightning Terminator*™ analogs

Nucleotide analog	DT_{50} in 1 mM NaN_3	
	No DTT	50 mM DTT
dA.I	3.6 ± 0.1	3.5 ± 0.1
dA.V	2.1 ± 0.1	2.0 ± 0.2
dA.VI	0.8 ± 0.1[a]	0.8 ± 0.1
dC.I	2.0 ± 0.3	1.6 ± 0.2
dC.V	1.2 ± 0.1	1.0 ± 0.2
dC.VI	0.6 ± 0.1[a]	0.6 ± 0.1
dG.I	9.2 ± 0.3	8.1 ± 0.2
dG.V	3.0 ± 0.1	2.9 ± 0.2
dG.VI	0.8 ± 0.1	0.8 ± 0.1
dU.I	2.1 ± 0.1	1.7 ± 0.1
dU.V	1.4 ± 0.1	1.3 ± 0.1
dU.VI	0.7 ± 0.1[a]	0.7 ± 0.1

[a]Transient product (TP) observed by gel electrophoresis; considered as cleaved product in DT_{50} value.

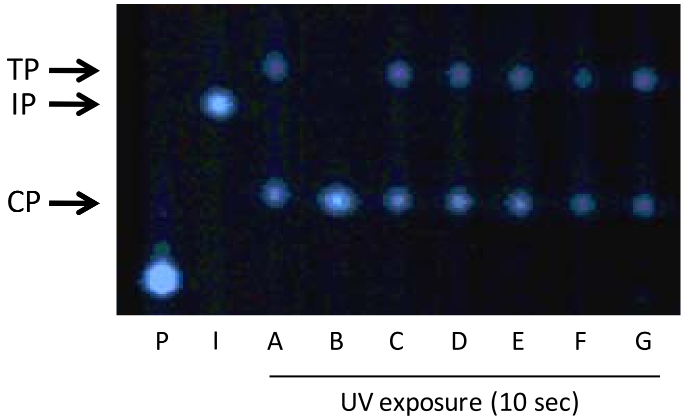

A. 1 mM NaN_3,
B. 1 mM NaN_3, 50 mM DTT
C. 1 mM NaN_3, 10 mM hydrazine
D. 1 mM NaN_3, 10 mM ethanolamine
E. 1 mM NaN_3, 10 mM Tris-HCl, pH 8.0
F. 1 mM NaN_3, 10 mM cysteamine
G. 50mM NaN_3, 20mM Tris-HCl, pH 8.0, 25% acetonitrile, 1 mg/mL BSA

***Figure S2*. DTT eliminates the nitroso intermediate (TP)**. Fluorescent gel image of UV photochemical cleavage experiment of **dU.VI** incorporated by Therminator polymerase. Lanes: “P” (primer) contains Therminator bound to oligoTemplate-4 hybridized with BODIPY-FL labeled primer-1 in 1x ThermoPol buffer, “I” (incorporation) contains that found in lane “P” plus 100 nM **dU.VI**. Reagents A-G listed as final concentrations in the key were added, and samples were exposed to 0.70 W/cm^2 365 nm light for 10 sec. “IP” denotes incorporated product, “CP” denotes cleaved product, and “TP” denotes transient product.

A.

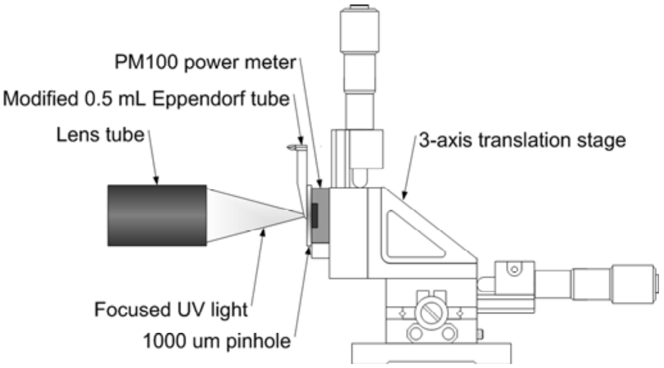

B.

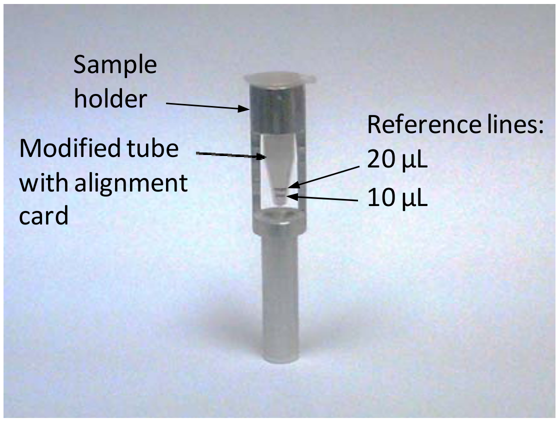

***Figure S3*. Optical set-up for UV photochemical cleavage measurements.** (A) Schematic of the modified 0.5 mL Eppendorf tube cut in half, PM100 power meter, a 1,000 µm pinhole cassette using a 3-axis manual translation stage to align the arc beam. (B) Sample holder and modified 0.5 mL Eppendorf tube with an internal alignment card to align the arc beam to the center of a 10 µL or 20 µL reaction sample.

X. References

[1] W. Wu, B. P. Stupi, V. A. Litosh, D. Mansouri, D. Farley, S. Morris, S. Metzker, M. L. Metzker, *Nucleic Acid Res.* **2007**, *35*, 6339-6349.
[2] V. A. Litosh, W. Wu, B. P. Stupi, J. Wang, S. E. Morris, M. N. Hersh, M. L. Metzker, *Nucleic Acid Res.* **2011**, *39*, e39.
[3] W. B. Smith, O. C. Ho, *J. Org. Chem.* **1990**, *55*, 2543-2545.
[4] J. E. T. Corrie, G. P. Reid, D. R. Trentham, M. B. Hursthouse, M. A. Mazid, *J. Chem. Soc., Perkin Trans. 1* **1992**, 1015-1019.
[5] F. Seela, X. Peng, in *Current Protocols in Nucleic Acid Chemistry* (Eds.: S. L. Beaucage, D. E. Bergstrom, G. D. Glick, R. A. Jones), John Wiley & Sons, Inc., **2005**, pp. 1.10.1-1.10.20.
[6] J. A. McCray, L. Herbette, T. Kihara, D. R. Trentham, *Proc. Natl. Acad. Sci. USA* **1980**, *77*, 7237-7241.
[7] G. H. McGall, A. D. Barone, M. Diggelmann, S. P. A. Fodor, E. Gentalen, N. Ngo, *J. Amer. Chem. Soc.* **1997**, *119*, 5081-5090.
